# Anti-Virulence Strategy of Novel Dehydroabietic Acid Derivatives: Design, Synthesis, and Antibacterial Evaluation

**DOI:** 10.3390/ijms24032897

**Published:** 2023-02-02

**Authors:** Puying Qi, Na Wang, Taihong Zhang, Yumei Feng, Xiang Zhou, Dan Zeng, Jiao Meng, Liwei Liu, Linhong Jin, Song Yang

**Affiliations:** National Key Laboratory of Green Pesticide, Key Laboratory of Green Pesticide and Agricultural Bioengineering, Ministry of Education, Center for R&D of Fine Chemicals of Guizhou University, Guiyang 550025, China

**Keywords:** natural products, pesticide discovery, dehydroabietic acid, antibacterial activity, virulence factors

## Abstract

Anti-virulence strategies are attractive and interesting strategies for controlling bacterial diseases because virulence factors are fundamental to the infection process of numerous serious phytopathogenics. To extend the novel anti-virulence agents, a series of dehydroabietic acid (DAA) derivatives decorated with amino alcohol unit were semi-synthesized based on structural modification of the renewable natural DAA and evaluated for their antibacterial activity against *Xanthomonas oryzae* pv*. oryzae* (*Xoo*), *Xanthomonas axonopodis* pv. *citri* (*Xac*), and *Pseudomonas syringae* pv. *actinidiae* (*Psa*). Compound **2b** showed the most promising antibacterial activity against *Xoo* with an EC_50_ of 2.7 μg mL^−1^. Furthermore, compound **2b** demonstrated remarkable control effectiveness against bacterial leaf blight (BLB) in rice, with values of 48.6% and 61.4% for curative and protective activities. In addition, antibacterial behavior suggested that compound **2b** could suppress various virulence factors, including EPS, biofilm, swimming motility, and flagella. Therefore, the current study provided promising lead compounds for novel bactericides discovery by inhibiting bacterial virulence factors.

## 1. Introduction

Plant diseases are significant threats to crop products worldwide due to the diverse bacterial virulence factors (VFs) promoting pesticide resistance. Rice is the main cash crop worldwide. However, *Xanthomonas oryzae* pv. *oryzae* (*Xoo*), which causes rice bacterial leaf blight (BLB), resulted in 20–60% yield losses during the post-pandemic period [1,2,3]. VFs significantly contribute to the BLB outbreak and are the main cause of pesticide resistance to traditional bactericides.

Instead of existing as planktonic cells, pathogens predominantly survived in the environment containing affluent biofilms, contributing significantly to their pathogenicity in natural environments. Bacterial infections are caused by numerous virulence factors, such as biofilm, extracellular polysaccharide (EPS), swimming motility, flagella, etc. The literature revealed that approximately 80% of bacterial infections involve biofilm that promotes resistance [4,5,6,7]. Meanwhile, EPS is a key element of bacterial biofilm and enhances cell adhesion on the surface of plants [8,9,10]. Additionally, swimming motility during the infection cycle allows bacteria to travel away from the harmful environment and into the plant tissue, which they can quickly colonize [6]. Swimming motion and bacterial flagella are closely related [11,12], as many bacteria use flagellum for swimming motility [10]. It is clear that VFs are important for bacterial pathogens and are sometimes required for infections [13]. Thus, discovering bacterial virulence inhibitors based on natural products is an appealing method for managing persistent bacterial diseases effectively.

**Figure 1:**
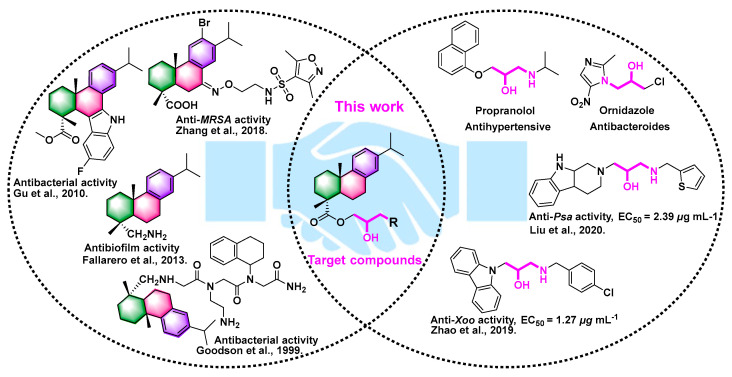
Some commercial and reported bioactive structures with an amino alcohol moiety and dehydroabietic acid and the method for producing target molecules [15,18,19,20,21,24].

The inhibiting bacterial mechanisms of bioactive natural products and their derivatives are well-established [14,15,16,17]. Notably, natural dehydroabietic acid (DAA) and its derivatives exhibit a broad range of biological activities, and it is a significant renewable forestry resource [17]. As shown in Figure 1, some dehydroabietic acid derivatives had outstanding antibacterial and antibiofilm activity [18,19,20,21]. Meanwhile, the amino alcohol unit exists widely in some drugs and antibacterials. Therefore, the above-mentioned compounds with amino alcohol and DAA moieties demonstrated strong antibacterial activities [14,22,23] and were used to develop pesticides to control several plant diseases. In this work, to excavate new anti-virulence agents, a series of amino alcohol-DAA compounds were prepared by adding an amino alcohol moiety to a DAA natural skeleton. The evaluation of the antibacterial mechanism also suggested that the DAA derivative **2b** functioned as a potential virulence factor inhibitor for regulating rice BLB.

## 2. Results and Discussion

### 2.1. Synthesis of DAA Derivatives

According to previous methods [14,22,23,24], a series of DAA derivatives with amino alcohol moiety were exquisitely synthesized using combinatorial chemistry. The design concept of the target compounds is shown in Figure 1. Intermediate 1 was obtained by introducing epoxybromopropane, and target compounds were synthesized through an epoxy ring opening reaction. The detailed experimental protocol for synthesizing compounds was found in the supporting information (The spectra data of title compound was displayed in Figures S1–S56).

### 2.2. Antibacterial Activities Evaluation of Target Compounds

**Figure 2:**
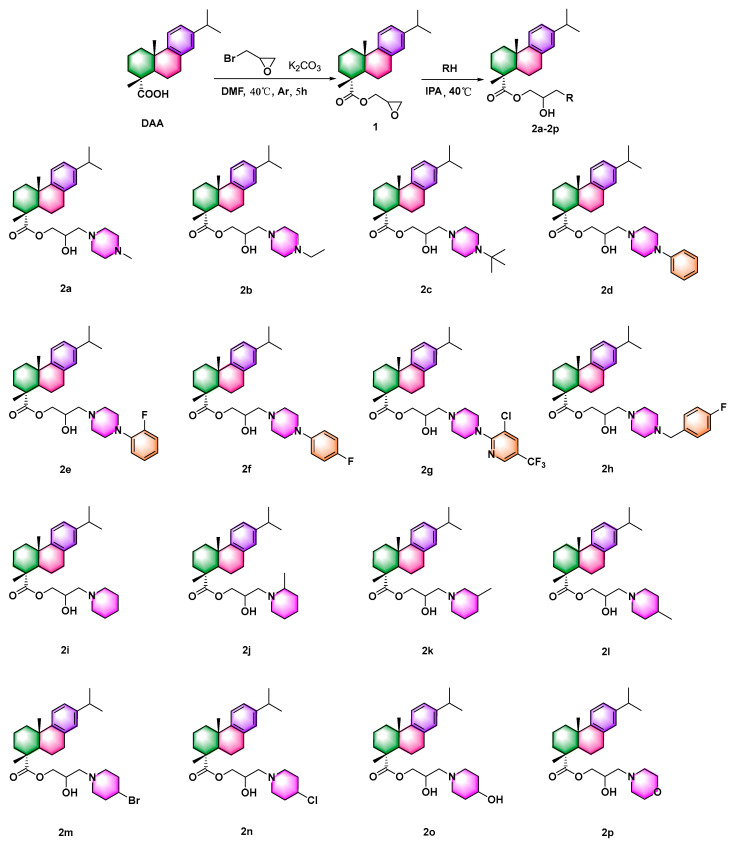
Synthesis route of target molecules **2a**–**2p**.

Some target compounds in Figure 2 and Table 1 exhibited strong antibacterial activity against *Xoo*. The inhibition ratios of compounds **2a**–**2c**, **2i**–**2m**, and **2o** against *Xoo* were 91.7%, 92.3%, 90.7%, 88.2%, 85.6%, 91.4%, 89.6%, 89.7%, and 91.9% at a concentration of 100 μg mL^−1^. Meanwhile, these compounds displayed excellent biological activities against *Xoo* at a dose of 50 μg mL^−1^. However, compounds **2d**, **2e**, **2f**, **2g**, and **2n** almost displayed negligible or no bioactivities at 50 and 100 μg mL^−1^. Interestingly, compared with compound **2h**, when a substituent group in the *N*-heterocyclic portion was lacking, compounds showed medium bioactivities, exampled by **2p**.

Based on the results mentioned above, additional antibacterial activity assays were carried out on *Xanthomonas axonopodis* pv. *citri* (*Xac*) and *Pseudomonas syringae* pv. *actinidiae* (*Psa*). Compounds **2a**, **2b**, **2c**, **2f**, **2i**, **2j**, **2k**, **2l**, and **2o** had strong bioactivities against *Xac* with inhibition ratios of 88.6%, 89.7%, 89.0%, 86.7%, 83.5%, 87.4%, 83.3%, 85.1%, and 89.1% at a concentration of 100 μg mL^−1^ (Table 1). However, as the control molecule, cyclohexanecarboxylic acid aminoalcohol derivatives did not exhibited antibacterial activity (Table S1), suggesting that retional design of title compounds can achieve the outstanding antibacterial activity. Additionally, these compounds demonstrated significant in vitro inhibitory activity against *Xac* at 50 μg mL^−1^. Furthermore, **2h**, **2m**, **2n**, and **2p** exhibited moderate biological activity, while the other target compounds exhibited low inhibitory activity. Nevertheless, all compounds displayed moderate or no antibacterial activity against *Psa* at 100 and 50 μg mL^−1^.

**Figure 3:**
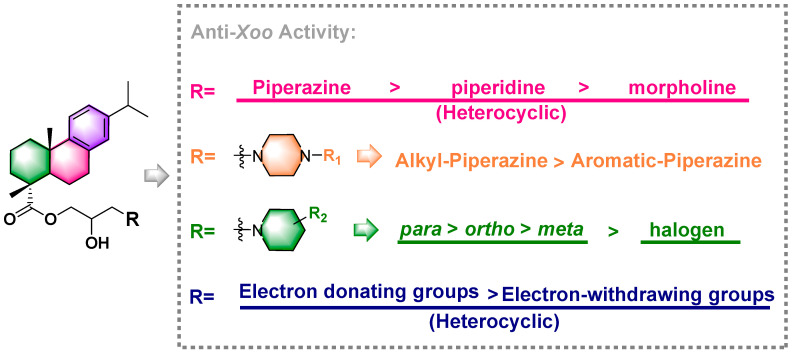
Overall structure-activity relationship analysis of all target compounds.

The effective concentration for 50% of maximal effect (EC_50_) of these compounds against *Xoo* was determined based on the excellent antibacterial activity of several compounds against *Xoo*. Compound **2b** exhibited the greatest inhibitory activity, with a value of 2.70 μg mL^−1^ (Table 2), and its MIC value was 10.8 μg mL^−1^ (Table S4). Furthermore, compounds **2a**, **2c**, **2h**, **2i**, **2j**, **2k**, **2l**, **2m**, and **2n** showed good inhibitory activities ranging from 3.2–7.0 μg mL^−1^. The EC_50_ values for each compound were 7.0, 3.2, 5.8, 3.2, 3.6, 4.1, 3.0, and 5.3 μg mL^−1^. However, compounds **2d** and **2p**, with values of 13.0 and 24.4 μg mL^−1^, had medium biological activity. None of the other compounds displayed antibacterial activity with EC_50_ values >100 μg mL^−1^, except compound **2o**; the EC_50_ of compound **2o** was 5.7 μg mL^−1^. Figure 3 summarizes the structure-activity relationship. The results showed that when three heterocyclic amines substituents were added, the EC_50_ of the target compounds decreased in the following order: piperazine derivatives (*N*-ethylpiperazine title compound with the highest EC_50_, 2.7 μg mL^−1^) > piperidine derivatives (EC_50_, 3.0–5.3 μg mL^−1^) > morpholine derivatives (EC_50_, 24.4 μg mL^−1^). In addition, antibacterial activity tests revealed that adding alkyl substituents to the piperazine ring was beneficial. However, the aromatic groups on the piperazine ring would have poor bioactivity. Furthermore, molecules with the same substituent at different positions of the piperidine ring exhibited different anti-*Xoo* activity, as evidenced by the fact that the EC_50_ of compounds decreased in the order, 4-methyl piperidine derivative (EC_50_ value, 3.0 μg mL^−1^) > 2-methyl piperidine derivative (EC_50_ value, 3.6 μg mL^−1^) > 3-methyl piperidine derivative (EC_50_ value, 4.1 μg mL^−1^). Finally, introducing electron-donating groups were useful, while reduced bioactivity was observed by conducting with electron-withdrawing groups.

### 2.3. Inhibitory Effects of Compound ***2b*** on the Xoo-Biofilm Formation and EPS Production

Biofilm, as one of the important VFs, is a significant and highly conserved structure for the bacterial community that acts as a crucial physical barrier against various complex environmental threats, including pH, temperature changes, host defense, and antibiotics [25,26,27]. Consequently, biofilm may significantly increase bacterial resistance [28,29,30,31] and is responsible for 80% of bacterial infections [4,32]. Additionally, EPS is the main component of the biofilm that promotes adherence to host surfaces [33,34]. However, *Xanthomonas* species such as *Xoo* and *Xanthomonas campestris* pv. *campestris* secreted the EPS known as xanthan gum. Therefore, xanthan gum would be used as a *Xoo* (a kind of *Xanthomonas*) indicator for detecting EPS production. To verify the experimental concentration is suitable, namely, that compound **2b** displayed anti-virulence activity rather than killing activity, the OD_595_ value was assayed (Table S2). Notably, when the dosage was 5.40 μg mL^−1^, compound **2b** did not show any bactericidal activity. Thus, the biofilm formation assay is carried out.

**Figure 4:**
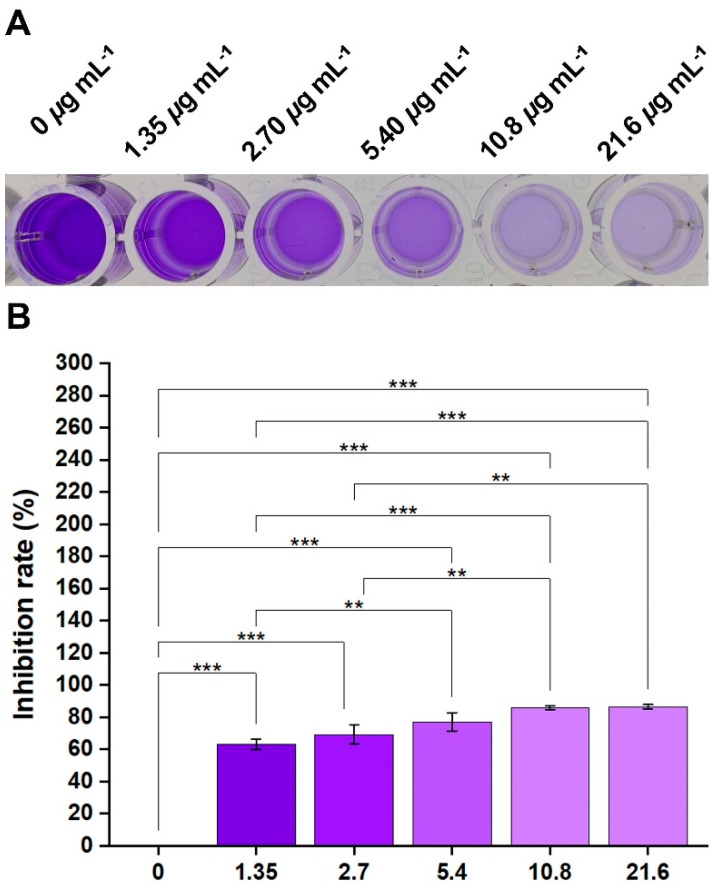
The quantitative assessment of crystal violet revealed the *Xanthomonas oryzae* pv. *oryzae*-biofilm inhibition of compound **2b**. (**A**) Biofilm staining imagins. (**B**) Inhibition rate of biofilm formation. [(**) *p* < 0.01, (***) *p* < 0.001 vs. 0].

**Figure 5:**
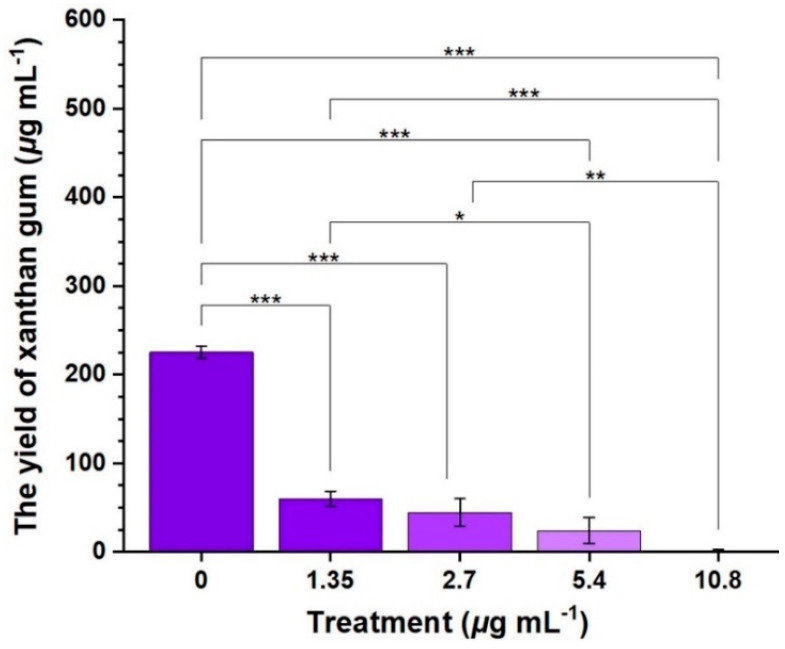
The production of xanthan gum [also known as an extracellular polysaccharide in *Xanthomonas oryzae* pv. *oryzae* (*Xoo*)] in the *Xoo*-biofilm after exposure to compound **2b** [(*) *p* < 0.05, (**) *p* < 0.01, (***) *p* < 0.001 vs. 0].

As shown in Figure 4, compound **2b** demonstrated an outstanding inhibitory effect for *Xoo*-biofilm formation. When concentrations of compound **2b** were 0, 1.35, 2.70, 5.40, 10.8, and 21.6 μg mL^−1^, respectively, the inhibition rates of bacterial biofilm products were 0, 63%, 69%, 77%, 86%, and 87%, respectively. Furthermore, as displayed in Figure 5, the production of xanthan gum was 225.3, 60.3, 44.3, 24.1, and 1.4 μg mL^−1^ after treatment with compound **2b** at dosages of 0, 1.35, 2.70, 5.40, and 10.8 μg mL^−1^, respectively. This suggests that compound **2b** might interfere with the biosynthesis process of xanthan gum. In brief, biofilm formation was constantly reduced with increasing concentrations, while the xanthan gum biosynthesis process decreased with increasing concentrations. Therefore, compound **2b** had the potential to inhibit bacterial biofilm formation.

### 2.4. The Inhibition Effect of Swimming Motility

**Figure 6:**
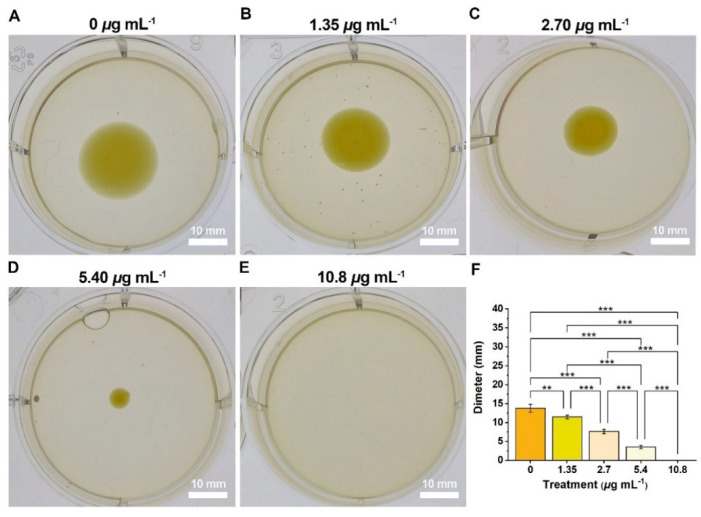
Compound **2b** inhibited the swimming motility of *Xanthomonas oryzae* pv. *oryzae* at concentrations of 0 (**A**), served as blank), 1.35 (**B**), 2.70 (**C**), 5.40 (**D**), and 10.8 (**E**) μg mL^−1^, and the swimming diameters were presented in (**F**). [(**) *p* < 0.01, (***) *p* < 0.001 vs. 0]. Scale bars are 10 mm.

Most phytopathogenic bacteria, including *Ralstonia solanacearum* and *Xanthomonas*, display good swimming motility [35], with bacterial swimming being the fastest mode of motility [12]. Swimming motility enables bacteria to sense environmental changes, avoid harmful environmental stressors, and move toward nutrients, consequently markedly enhancing bacterial fitness [36,37]. Figure 6 showed that swimming diameter decreased with increasing doses. The swimming diameters at concentrations of 0, 1.35, 2.70, 5.40, and 10.8 μg mL^−1^ were 13.8, 11.5, 7.7, 3.6, and 0 mm, respectively; bacterial swimming motility was gradually weakened. Compound **2b** inhibited bacterial motility levels, lowered fitness, and decreased infections.

### 2.5. The Inhibition Effect of Xoo-Flagellum Assembly

The flagellum is the important bacterial organelle responsible for swimming motility [38], comprising the filament, hook, and basal body [38]. The ability of bacterial cells to move toward beneficial environments and escape harmful environmental stressors, and the swimming motility mediated by the bacterial flagellum, play a significant role in the bacterial infection cycle, increasing the probability of cells interacting with hosts’ surfaces [39,40]. The ability was extremely beneficial for enhancing search potency, enabling bacteria to seek advantages and avoid disadvantages. It revealed that a bacterium’s virulence toward its host was significantly influenced by flagellum-mediated swimming motility [41,42]. Swimming motility, chemotaxis, and host cell invasion increased the likelihood of bacteria interacting with host organism surfaces during the infection [13]. Furthermore, the mutation of flagella-related genes resulted in a loss of motility, reduction in bacterial colonization, downregulation of host cell immunity, decrease the virulence, and reduction in pathogenicity [13,42]. Thus, the flagellum, which also contains swimming motility, initial attachment, tissue invasion, and biofilm formation, contributes to bacterial virulence and infection [13,42].

**Figure 7:**
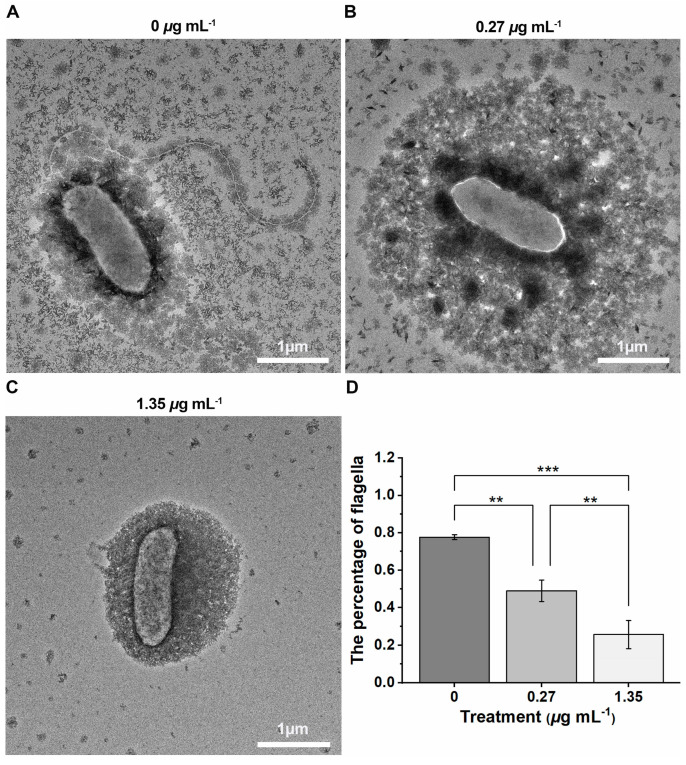
The *Xanthomonas oryzae* pv. *oryzae*-flagella assembly interfered with compound **2b** at doses 0 (**A**), 0.27 (**B**), and 1.35 μg mL^−1^ (**C**), presented in the transmission electron microscope, and the percentage of flagella was showed in (**D**) [(**) *p* < 0.01, (***) *p* < 0.001 vs. 0]. Scale bar = 1 μm.

As shown in Figure 7, the percentage of flagellum assembly was 0.78, 0.49, and 0.26 at doses of 0, 0.27, and 1.35 μg mL^−1^. These findings show compound **2b** strongly interfered with the flagellum assembly process at concentrations 0.27 and 1.35 μg mL^−1^. Moreover, bacterial flagellum assembly interfered similarly at a dose of 0 μg mL^−1^ (served as a control). The primary cause was that some bacteria had mature flagellum while others were at the initiation or growth stage. The assembly and disassembly of the flagellum, a dynamic nanostructure, was coupled with the cell cycle [43]. Therefore, we can infer that the cells in the initiation and growth stages did not complete the assembly of their flagella. These findings suggested compound **2b** might significantly interfere with bacterial flagellum assembly and cause virulence downregulation.

### 2.6. Cell Membrane Morphology Analysis

**Figure 8:**
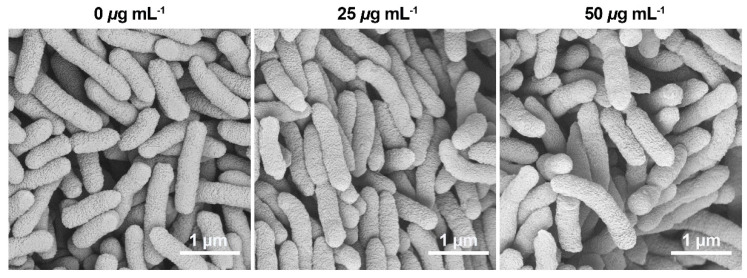
Scanning electron microscope images of the morphology of the *Xanthomonas oryzae* pv. *oryzae* cell membrane induced by compound **2b** at doses 0 (served as blank), 25, and 50 μg mL^−1^ 24 h. Scale bars = 1 μm.

The cell membrane plays a role in nutrient intake, biomacromolecule transportation, and signal transduction, as well as being a considerable barrier to complicate external environmental stresses [31]. Thus, the cell membrane plays a significant role in these physiological and biochemical processes. A loss in bacterial cell membrane integrity results in increased membrane permeability, which impacts cell physiology and metabolism, leading to cytoplasm leakage and cell death [32,44,45,46,47]. Additionally, the membrane reportedly plays a crucial role in preserving cell homeostasis, with a loss of membrane integrity leading to the end of the cellular life cycle [30,48]. Figure 7 indicated that compound **2b** affected flagella assembly but not the morphology of cellular membranes. In addition, the properties of VF inhibitors did not hinder cellular development and proliferation at a low dose of compound **2b**. Therefore, SEM technology was used to analyze the morphology of cell membranes. Figure 8 showed the morphology observation results: bacterial morphology was unchanged after treatment with varying doses of 0, 25, and 50 μg mL^−1^ and showed a smooth surface and rod-shaped structure. As a result, compound **2b** only affected biological processes related to bacterial VFs and did not affect the structure of bacterial cell membranes or interfere with normal cellular growth and proliferation.

### 2.7. Pathogenicity of the Xoo Interacted with Compound ***2b***

Several VFs strongly correlate with the pathogenicity of infections [49]. VFs secreted by different bacteria are crucial for promoting cell colonization and enhancing pathogenicity during infection [50,51]. Pathogens use several VFs to overcome the host’s defense system [52]. Thus, bacterial pathogenicity depends on their ability to secrete numerous VFs [53]. *R. solanacearum* uses various VFs to infect plants and cause a withering phenomenon. EPS, also known as xanthan gum in *Xoo*, lipopolysaccharides, extracellular enzymes (including amylase, endoglucanase, polygalacturonate lyase, and protease), and biofilm are some significant VFs that have been found in many bacteria [54,55]. Additionally, bacterial motility and flagella facilitate cell colonization and adhesion [55]. *Erwinia amylovora* develops many VFs to overcome the plant immune system and facilitate infection [56]. The bacterium *Xanthomonas campestris* pv. *campestris* encodes for type III secretion system-dependent transcription activator-like effectors, among other VFs [54]. Massive VFs may interfere with the host’s vascular system and cause wilting symptoms [57]. Cell and tissue damage caused by *Staphylococcus aureus* and *Nocardia adhesion* and invasion are important pathogenetic factors [26]. A thorough analysis revealed that antibacterial peptides reduced bacterial pathogenicity by inhibiting VFs activity [58]. Disrupting the secretion and assembly of VFs has been associated with several anti-virulence compounds, including those that inhibit biofilm formation, lower EPS production, and interfere with initial bacterial adhesion [59]. The mechanism of VFs in host infection has been gradually explored and excavated thanks to advancements in molecular biology techniques and a comprehensive understanding of VFs. Targeting VFs would be a desirable and practical method to eliminate or reduce bacterial pathogenicity and weaken resistance by interfering with virulence biosynthesis processes as opposed to cell death because VFs are crucial for bacterial infections [6,60,61].

**Figure 9:**
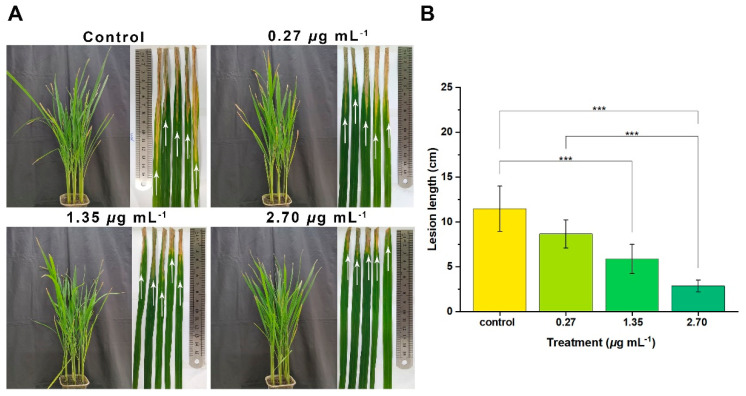
Effects of compound **2b** on the representative *Xanthomonas oryzae* pv. *oryzae*’s (*Xoo*) responses to the rice bacterial leaf blight disease. (**A**) The leaf-clipping method was used to co-incubate rice leaves with the *Xoo* cell suspension after exposure to doses 0 (served as blank), 0.27, 1.35, and 2.70 μg mL^−1^ of compound **2b** for 24 h. (**B**) The length of a lathy lesion of rice leaves was measured in *Xoo* at various doses of compound 2b [(***) *p* < 0.001 vs. 0].

Thus, the analysis of *Xoo*-pathogenicity was performed based on the results of the above-mentioned completed experiments, and compound **2b** significantly disrupted *Xoo*-virulence biological processes. As shown in Figure 9, the *Xoo* cells suspension was co-incubated with compound **2b** for one day at the different doses of 0 (referred to as the control), 0.27, 1.35, and 2.70 μg mL^−1^. The rice plant was then inoculated with the above-mentioned cell suspension using the leaves clipping method. Subsequently, the *Xoo*-inoculated rice plant was cultured for 14 days. Finally, samples from the control treatment showed more lathy lesions with a length of 11.5 cm. The lesion lengths of other treatments, 0.27, 1.35, and 2.70 μg mL^−1^ were 8.7, 5.9, and 2.9 cm, respectively. As a result of interfering with the manufacture of multiple bacterial VFs, compound **2b** demonstrated the ability to suppress *Xoo*-VFs and strongly reduce bacterial pathogenicity. Compound **2b** would be a potent virulence inhibitor to manage rice BLB successfully.

### 2.8. In Vivo Anti-Xoo Effect of Compound ***2b*** Controlling Bacterial Disease at 200 μg mL^−1^

**Figure 10:**
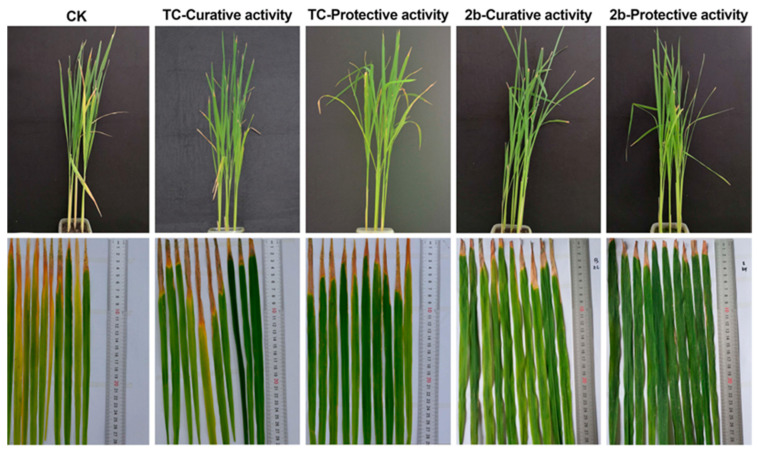
In vivo antibacterial activity of compound **2b** and TC against rice bacterial leaf blight at 0 μg mL^−1^ (served as CK) and 200 μg mL^−1^; **2b**: target compound **2b**; TC: thiodiazole copper; CK: blank control.

Although target compound **2b** demonstrated excellent antibacterial activity and an alluring anti-VFs mechanism, the preservation of the crops was our ultimate goal. Therefore, the antibacterial activity of compound **2b** was investigated in vivo to verify its anti-*Xoo* activity. Compound **2b** demonstrated excellent curative and protective activities, and the control efficiency was 48.6% and 61.4%, respectively (Figure 10 and Table 3). Compound **2b** exhibited significant antibacterial activity in vitro and exceptional control efficiency in vivo.

### 2.9. The Toxicity Evaluation of Compound ***2b*** on Rice Leaves at 0, 200, and 500 μg mL^−1^

**Figure 11:**
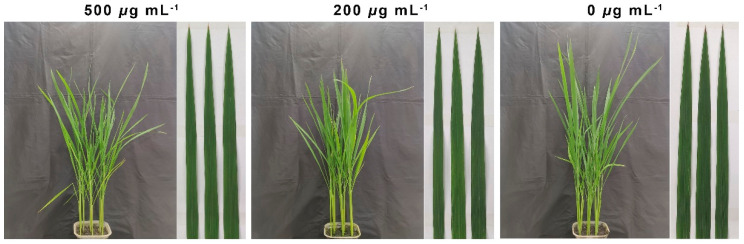
The phytotoxicity assessment of compound **2b** on rice leaves after co-culturing it for seven days at 0, 200, and 500 μg mL^−1^.

Although compound **2b** showed remarkable antibacterial activity in vitro and in vivo, its phytotoxicity for the target crop remained unknown. Therefore, the phytotoxicity of compound **2b** was assessed at doses of 0, 200, and 500 μg mL^−1^. As shown in Figure 11 and Table S3, compound **2b** did not affect the normal growth of rice leaves and did not cause any lesions or necrosis. Furthermore, as found in Figure S57, predicting results suggested that the title compounds exhibited acceptable physicochemical properties. Thus, it would be a highly effective and low-risk green pesticide option.

## 3. Materials and Methods

### 3.1. Instruments and Chemicals

Thin-layer chromatography plates were used to monitor organic reaction processes (Yantai Jiangyou Silica Development Co., Ltd., Silica HSGF254, Shandong, China) [51]. Bruker AG-400 (Switzerland) and JEOLECX-500 (Japan) were used to measure the ^1^H and ^13^C nuclear magnetic resonance spectra of the DAA derivatives using CDCl_3_ (Anhui Zesheng Technology Co., Ltd, Energy-Chemical, China) or DMSO-*d*_6_ (Anhui Zesheng Technology Co., Ltd, Energy-Chemical, China) as the solvent and internal standard, respectively. Related chemical shifts and coupling constants (J) were represented as parts per million and hertz, respectively. A Thermo Scientific Q Exactive UItiMate 3000 instrument was used to determine the High-resolution mass spectrometry (HRMS) of DAA derivatives. FEI Talos, F200C electron microscope (FEI, USA) images were collected using a transmission electron microscope (TEM) at a voltage of 200 kV. The morphology of phytobacteria was investigated using an FEI Nova NanoSEM 450 (FEI, USA) instrument. DAA (purity > 75%) was used as the starting material purchased from Anhui Zesheng Technology Co., Ltd, Energy-Chemical, China.

### 3.2. Antibacterial Activity Evaluation In Vitro and In Vivo

Analyses of the biological activity of the target molecules against the three plant bacteria *Xoo*, *Xac*, and *Psa* were performed in vitro and in vivo [62,63].

### 3.3. Xoo-Biofilm Formation and EPS Production Analysis

A *Xoo*-biofilm formation assay using the crystal violet staining method was performed to assess the antibacterial biofilm function of compound **2b** [14,62,63]. Initially, a 96-well plate with 200 μL of nutrient broth medium was used, and the bacterial cell suspension was adjusted to 0.1 (OD_595nm_). Different doses of compound **2b** were added, and the mixture was incubated at 28 ℃ for 72 h. Following that, 200 μL of medium from each well was aspirated and washed three times with sterile water. Subsequently, bacteria were fixed with 200 μL of Carnoy’s fluid for 30 min and stained with 1% crystal violet staining solution for 15 min. The crystal violet solution was then removed from the 96-well plate, and the residue was dissolved using 95% ethanol. Finally, based on the phenol-sulfuric acid standard curve, the OD_470 nm_ value was measured to determine the inhibitory effect of biofilm formation and EPS production.

### 3.4. Swimming Motility Assay

An examination of bacterial swimming motility was performed to assess the inhibitory effect of compound **2b** on *Xoo* cell motility. Based on previous studies but with a slight modification, *Xoo* cells suspension was adjusted to 0.2 (OD_595nm_), and 2 μL of cell suspension was inoculated in the center of motility plates (0.3% beef extract, 0.5% peptone, 0.1% yeast powder, 1% glucose, 0.5% agar powder, and pH 7.2) with various doses (0, 1.35, 2.70, 5.40, and 10.8 μg mL^−1^) of compound **2b** at 28 ℃ for 72 h. Finally, the swimming diameters for three biological replicates were observed and measured [62].

### 3.5. Morphology Observation of TEM

Target compound **2b** was co-incubated with bacterial cells (OD_595 nm_ = 0.1) in a shaker-incubator at 28 ℃, 180 rpm for 18 h at various doses (0, 0.27, and 1.35 μg mL^−1^) [10,52,53]. *Xoo* cells were then fixed in the copper grids and stained for TEM observation with 1% phosphotungstic acid.

### 3.6. Morphology Observation of Scanning Electron Microscope (SEM)

*Xoo* cells with an initial OD_595 nm_ = 0.1 were co-incubated with compound **2b** for 12 h at different doses (0, 25, and 50 μg mL^−1^) in a shaker incubator (28 ℃, 180 rpm). Subsequently, 2.5% glutaraldehyde was used to fix the *Xoo* cells overnight after they had been collected via centrifugation and resuspension. The glutaraldehyde solution was then removed, and the residue was dehydrated using ethanol at various concentrations (30%, 50%, 70%, 90%, and 100%). Finally, samples were freeze-dried and gold-coated for SEM observation [62,64].

### 3.7. Pathogenicity Assay

A pathogenicity assay assessed the bacterial virulence after 24 h of interaction between *Xoo* cells and compound **2b** at various doses (0, 0.27, 1.35, and 2.70 μg mL^−1^). Subsequently, the *Xoo* cell suspension was adjusted to 0.5 (OD_595 nm_), and three biological replicates of rice leaves were inoculated using the leaf-clipping method [14,62]. The leaf lesion lengths were observed and measured after fourteen days, and the one-way analysis of variance was used to evaluate the lesion length data.

## 4. Conclusions

The rice BLB caused by *Xoo* secreting many bacterial VFs is a sustained global danger to agricultural products. Our completed research indicates that compound **2b** would be a desirable and potent bactericide candidate for preventing rice BLB by specifically targeting *Xoo*-VFs. Initially, DAA was believed to be a forestry resource with significant added value due to its wide range of biological activities. Subsequently, many novel DAA derivatives were ingeniously partially synthesized, and their anti-*Xoo* properties were evaluated in vitro. All biological analyses showed that novel DAA derivatives containing amino alcohol fragments had remarkable anti-virulence properties that functioned by inhibiting a variety of bacterial VFs, including EPS, biofilm, swimming motility, and flagella. In vivo, compound **2b** showed excellent curative and protective properties, with minimal phytotoxicity at 200 and 500 μg mL^−1^. Finally, we made some preliminary speculations about the mechanism by which **2b** inhibited flagella and swimming motility, interfered with EPS secretion and cell adhesion, prevented biofilm formation and bacterial colonization, and decreased bacterial pathogenicity.

## Figures and Tables

**Table 1:** Antibacterial bioactivity of compounds against the phytopathogenic bacteria *Xoo*, *Xac*, and *Psa* in vitro.

Compds	Inhibition Ratio (%)					
	*Xoo*		*Xac*		*Psa*	
	100 μg mL^−1^	50 μg mL^−1^	100 μg mL^−1^	50 μg mL^−1^	100 μg mL^−1^	50 μg mL^−1^
**DAA**	43.9 ± 2.7	38.4 ± 3.4	56.9 ± 3.4	44.1 ± 12.6	39.8 ± 9.5	34.5 ± 10.2
**2a**	91.7 ± 0.8	91.6 ± 0.3	88.6 ± 1.5	87.5 ± 0.4	48.3 ± 6.8	41.2 ± 1.6
**2b**	92.3 ± 0.3	91.0 ± 0.3	89.7 ± 0.2	89.4 ± 0.3	40.6 ± 5.1	38.1 ± 7.5
**2c**	90.7 ± 0.3	89.2 ± 1.4	89.0 ± 1.3	85.9 ± 1.5	54.3 ± 7.5	49.6 ± 6.8
**2d**	0	0	0	0	0	0
**2e**	0	0	0	0	0	0
**2f**	0	0	86.7 ± 0.1	85.9 ± 0.1	17.7 ± 3.1	16.8 ± 7.7
**2g**	10.7 ± 3.8	0	0	0	0	0
**2h**	65.9 ± 0.5	64.2 ± 0.2	41.8 ± 7.0	37.2 ± 5.2	0	0
**2i**	88.2 ± 0.7	79.0 ± 0.8	83.5 ± 1.1	79.0 ± 2.9	53.2 ± 7.1	50.1 ± 5.5
**2j**	85.6 ± 0.4	84.3 ± 0.3	87.4 ± 1.8	84.0 ± 0.5	58.1 ± 0.3	52.6 ± 4.2
**2k**	91.4 ± 0.1	88.9 ± 0.4	83.3 ± 0.8	82.6 ± 0.9	36.0 ± 1.6	7.3 ± 5.0
**2l**	89.6 ± 3.1	87.5 ± 1.2	85.1 ± 2.4	82.1 ± 1.4	55.2 ± 1.5	50.1 ± 0.7
**2m**	89.7 ± 0.6	89.4 ± 1.2	47.2 ± 5.4	44.3 ± 0.7	47.5 ± 9.9	46.6 ± 1.6
**2n**	11.3 ± 2.5	0	48.4 ± 2.7	45.0 ± 1.2	45.9 ± 2.8	40.8 ± 8.5
**2o**	91.9 ± 0.9	91.8 ± 0.3	89.1 ± 1.5	88.5 ± 0.5	51.0 ± 5.8	48.2 ± 5.5
**2p**	62.2 ± 3.5	54.5 ± 1.2	48.7 ± 9.2	40.0 ± 3.9	30.4 ± 1.2	21.8 ± 1.3
**TC**	85.1 ± 5.3	46.8 ± 2.2	56.3 ± 3.2	32.3 ± 2.1	63.1 ± 6.2	33.6 ± 2.2

Thiodiazole copper (TC), Xanthomonas oryzae pv. oryzae (Xoo), Xanthomonas axonopodis pv. citri (Xac), and Pseudomonas syringae pv. actinidiae (Psa).

**Table 2:** EC_50_ of highly bioactive compounds against *Xoo*.

Compds	Regression Equation	R^2^	EC_50_ (μg mL^−1^)	EC_50′_ (μM)
**2a**	y = 2.6697x + 2.7486	0.9182	7.0 ± 0.5	15.3
**2b**	y = 2.0052x + 4.1343	0.9558	2.7 ± 0.3	5.7
**2c**	y = 1.4267x + 4.5965	0.9369	3.2 ± 0.9	6.4
**2d**	y = 2.3605x + 2.3735	0.8867	13.0 ± 1.7	25.1
**2e**			>100	
**2f**			>100	
**2g**			>100	
**2h**	y = 1.0065x + 4.2365	0.9829	5.8 ± 0.7	10.5
**2i**	y = 2.0242x + 3.9693	0.9066	3.2 ± 0.7	7.2
**2j**	y = 1.8918x + 3.9619	0.8840	3.6 ± 0.5	8.1
**2k**	y = 1.9734x + 3.7912	0.9149	4.1 ± 0.8	9.2
**2l**	y = 1.4200x + 4.5751	0.9630	3.0 ± 0.4	6.7
**2m**	y = 1.5424x + 3.8815	0.9288	5.3 ± 0.8	10.2
**2n**			>100	
**2o**	y = 4.5670x + 1.5782	0.9943	5.7 ± 0.5	12.5
**2p**	y = 0.6191x + 4.1400	0.9510	24.4 ± 1.2	55.0
**TC**	y = 5.4033x − 2.3402	0.9621	61.2 ± 5.2	186.6

Thiodiazole copper (TC), *Xanthomonas oryzae* pv. *oryzae* (*Xoo*).

**Table 3:** Compound **2b** and TC at 200 μg mL^−1^ in vivo demonstrated both curative and protective activities against rice bacterial leaf blight under greenhouse conditions.

Treatment	Curative Activity (14 Days after Spraying)			Protection Activity (14 Days after Spraying)		
	**Morbidity (%)**	**Disease Index (%)**	**Control E** **ffi** **ciency (%)**	**Morbidity (%)**	**Disease Index (%)**	**Control E** **ffi** **ciency (%)**
**2b**	100	40.00	48.57	100	30.00	61.43
**TC**	100	57.78	25.72	100	62.22	20.00
**CK**	100	77.78	/	100	77.78	/

**2b**: target compound **2b**; TC: thiodiazole copper; CK: blank control.

## Data Availability

Not applicable.

## Supplementary Materials

The following supporting information can be downloaded at: https://www.mdpi.com/article/10.3390/ijms24032897/s1.

## Abbreviations

Dehydroabietic acid	DAA
*Xanthomonas oryzae* pv. *oryzae*	*Xoo*
*Xanthomonas*. *axonopodis* pv *citri*	*X* *ac*
*Pseudomonas syringae* pv. *actinidiae*	*Psa*
Bacterial leaf blight	BLB
Extracellular polysaccharide	EPS
Virulence factors	VFs
Effective concentration for 50% of maximal effect	EC_50_
Thiodiazole copper	TC
