# Supplementary material for: Anti-Virulence Strategy of Novel Dehydroabietic Acid Derivatives: Design, Synthesis, and Antibacterial Evaluation
Source: Int J Mol Sci. 2023 Feb 2;24(3):2897. doi: 10.3390/ijms24032897 (PMC9917773; doi:10.3390/ijms24032897)
Supplement: Supplementary file 1 [file ijms-24-02897-s001.zip › ijms-2155608-supplementary.pdf]

# Anti-virulence strategy of novel dehydroabietic acid derivatives: Design, synthesis, and antibacterial evaluation

Puying Qi, Na Wang, Taihong Zhang, Yumei Feng, Xiang Zhou *, Dan Zeng, Jiao Meng, Liwei Liu, Linhong Jin, Song Yang *

National Key Laboratory of Green Pesticide, Key Laboratory of Green Pesticide and Agricultural Bioengineering, Ministry of Education, Center for R&D of Fine Chemicals of Guizhou University, Guiyang 550025, China

* Corresponding author.

E-mail: zhoux1534@163.com or xiangzhou@gzu.edu.cn (X.Z.);

# Content

1. Experimental section ........ 3

1.1 *In vitro* antibacterial bioassay (turbidimeter test) ........ 3

1.2 *In vivo* bioassay rice bacterial leaf blight ........ 4

1.3 Synthesis for intermediates and target compounds ........ 4

1.3.1 General synthetic procedure for the intermediate 1 ........ 4

1.3.2 General synthetic procedures for the target compounds 2a-2p ........ 5

1.4 Table S1. Antibacterial bioactivity of cyclohexanecarboxylic acid derivatives against the phytopathogenic bacteria *Xoo* and *Xac* in vitro. ........ 5

1.5 Table S2. The absorbance at 595 nm of compound 2b against bacteria *Xoo* at the concentration of 0 and 5.4 μg $mL^{-1}$. ........ 5

1.6 Table S3. The logS and logP of resulting compounds. ........ 5

1.7 Table S4. The MIC value of compound 2b. ........ 6

2. $^{1}H$ NMR, $^{13}C$ NMR, $^{19}F$ NMR and HRMS Spectra (Figure S1-S56) ........ 6

3. Figure S57. The physicochemical properties of resulting compounds. ........ 47

# 1. Experimental section

## 1.1 *In vitro* antibacterial bioassay (turbidimeter test)

In our study, all the synthesized target compounds were evaluated for their antibacterial activities against *Xoo*, *Psa*, and *Xac* by the turbidimeter test *in vitro*.[1] Dimethylsulfoxide in sterile distilled water served as a blank control, Thiodiazole Copper served as positive controls. Approximately 40 $\mu$L of solvent NB (1.5 g beef extract, 2.5 g peptone, 0.5 g yeast powder, 5.0 g glucose, and 500 mL distilled water; pH = 7.0–7.2) containing *Xoo* (*Psa* or *Xac*), incubated on the phase of logarithmic growth, was added to 5 mL of solvent NB containing different concentrations of the test compounds and positive control, such as 100, 50 $\mu$g/mL (for preliminary bioassays), 20, 10, 5, 2.5, 1.25 $\mu$g mL$^{-1}$ or 10, 5, 2.5, 1.25, 0.625 $\mu$g mL$^{-1}$ (depending on the bioactivity of different compounds, the concentrations were chosen in two times decline trend to make sure the $EC_{50}$ values are inside the concentration ranges tested). The inoculated test tubes were incubated at 28 ± 1 °C and continuously shaken at 180 rpm for 24-48 h until the bacteria were incubated on the logarithmic growth phase. The growth of the cultures was monitored on a microplate reader by measuring the optical density at 595 nm ($OD_{595}$) given by turbidity corrected values = $OD_{bacterial\ wilt} - OD_{no\ bacterial\ wilt}$, and the inhibition rate I was calculated by I = (C − T)/C × 100%. C is the corrected turbidity values of bacterial growth on untreated NB (blank control), and T is the corrected turbidity values of bacterial growth on treated NB. By using the SPSS 20.0 software and the obtained inhibition rates at different concentrations, a regression equation was provided. The results of antibacterial activities (expressed by $EC_{50}$) against *Xoo*, *Psa*, and *Xac* were

calculated from the equation and the value was within the concentration ranges. The experiment was repeated three times.

### 1.2 *In vivo* bioassay rice bacterial leaf blight

*In vivo* antibacterial activities of compound **2b** against rice bacterial leaf blight were performed based on our reported method. Thiodiazole copper (TC) were used as the positive controls, and the rice plants (variety Xiangliangyou 900) grown in the maximum leaf stage were used in this experiment. For the protective activity, different adjuvants were added into a compound **2b** solution at 200 $\mu$g mL$^{-1}$ to get a mixture that included organic silicone, organic fluorine, and orange peel essential oil with a dose of 0.1% (v/v), respectively. Then the mixture was evenly sprayed on the rice leaves; 24 h after spraying, *Xoo* cell solution ($OD_{595}$ = 0.8−1.0) was inoculated on the rice leaves. Control efficiency $I$ (%) = (C - T) / C ×100.

### 1.3 Synthesis for intermediates and target compounds

#### 1.3.1 General synthetic procedure for the intermediate 1

Epibromohydrin (1.2 mmol) and dehydroabietic acid (1.0 mmol) were dissolved in the 5 mL anhydrous DMF solvent, which was added into 15 mL pressure-resistant reaction tube contained $K_2CO_3$ (1.2 mmol) protected by Ar atmosphere. After stirring the mixture at 60 °C for 8 hours, 30 mL ethyl acetate was added into the mixture. The organic layer was washed 3 times by 45mL saturated $NH_4Cl$ solution, water, brine, dried with sodium sulfate, filtered, and followed by the removal of the solvent under vacuum. The pure intermediate could be obtained by column chromatography using the eluent (PE:EA, V:V = 5:1).

### 1.3.2 General synthetic procedures for the target compounds 2a-2p

Generally, to a 15 mL pressure-resistant reaction tube, compound **1** (1.12 mmol) were dissolved in 5 mL dry isopropanol, then corresponding amines (2.24 mmol) were added and heated to 40 °C for 6 hours. After that, the removal of the solvent under vacuum. Finally, the crude residue was further purified by column chromatography on a silica gel using $CH_2Cl_2$ and MeOH (200:1-20:1, V/V) as the eluent to afford the desired product **2a**. The synthesis of all compounds (**2b-2p**) was carried out as the synthetic protocol of **2a**.

### 1.4 Table S1. Antibacterial bioactivity of cyclohexanecarboxylic acid derivatives against the phytopathogenic bacteria *Xoo* and *Xac* in vitro.

| Compounds | Inhibition ratio (%) | | | |
|---|---|---|---|---|
| | ***Xoo*** | | ***Xac*** | |
| | **100 μg mL$^{-1}$** | **50 μg mL$^{-1}$** | **100 μg mL$^{-1}$** | **50 μg mL$^{-1}$** |
| 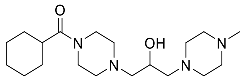 | 13.1±2.5 | 0 | 0 | 0 |
| 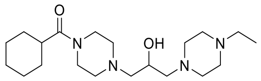 | 0 | 0 | 0 | 0 |
| 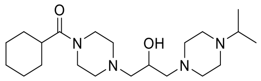 | 16.8±4.9 | 11.5±4.5 | 0 | 0 |
| 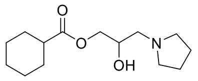 | 0 | 0 | 0 | 0 |

### 1.5 Table S2. The absorbance at 595 nm of compound 2b against bacteria *Xoo* at the concentration of 0 and 5.4 μg mL$^{-1}$.

| Treatment (μg mL$^{-1}$) | $OD_{595}$ |
|---|---|
| 0 | 0.466±0.018 |
| 5.4 | 0.462±0.008 |

### 1.6 Table S3. The logS and logP of resulting compounds.

| Compds | logS | logP |
|---|---|---|

| | | |
|---|---|---|
| **2a** | -4.512 | 4.310 |
| **2b** | -4.649 | 4.577 |
| **2c** | -4.977 | 5.255 |
| **2d** | -5.326 | 5.783 |
| **2e** | -5.465 | 5.943 |
| **2f** | -5.414 | 5.874 |
| **2g** | -5.969 | 6.444 |
| **2h** | -5.097 | 5.431 |
| **2i** | -5.107 | 5.398 |
| **2j** | -5.100 | 5.444 |
| **2k** | -5.291 | 5.618 |
| **2l** | -5.379 | 5.657 |
| **2m** | -5.361 | 5.291 |
| **2n** | -5.367 | 5.285 |
| **2o** | -4.769 | 4.031 |
| **2p** | -4.723 | 4.358 |

logS: log of the aqueous solubility, Optimal: -4~0.5 log mol $L^{-1}$; logP: log of the octanol/water partition coefficient, Optimal: 0~3.

**1.7 Table S4. The MIC value of compound 2b.**

| Compound | MIC |
|---|---|
| 2b | 10.8 μg $mL^{-1}$ |

## 2. $^{1}$H NMR, $^{13}$C NMR, $^{19}$F NMR and HRMS Spectra (Figure S1-S56)

**Dehydroabietic acid (DAA)**

**$^{1}$H NMR (500 MHz, DMSO-*$d_6$*)** δ 12.17 (s, 1H, -COOH), 7.15 (d, *J* = 8.0 Hz, 1H, phenyl-H), 6.97 (d, *J* = 8.0 Hz, 1H, phenyl-H), 6.84 (s, 1H, phenyl-H), 2.85 – 2.75 (m, 2H, phenyl-cyclohexane-C<u>H</u>$_2$), 2.29 (d, *J* = 13.3 Hz, 1H, phenyl-cyclohexane-C<u>H</u>-$CH_2$),

2.02 (dd, *J* = 12.4, 2.0 Hz, 1H, $C\underline{H}(CH_3)_2$), 1.81 – 1.69 (m, 2H, cyclohexane-$C\underline{H_2}$), 1.68 – 1.60 (m, 2H, cyclohexane-$C\underline{H_2}$), 1.56 (m, 1H, phenyl-cyclohexane-1/2$C\underline{H_2}$), 1.39 (m, 1H, phenyl-cyclohexane-1/2$C\underline{H_2}$), 1.33 – 1.21 (m, 2H, cyclohexane-$CH_2C\underline{H_2}CH_2$), 1.16 (d, *J* = 1.0 Hz, 6H, $CH(C\underline{H_3})_2$), 1.14 (s, 3H, $CC\underline{H_3}$), 1.12 (s, 3H, $CC\underline{H_3}COOH$). **$^{13}$C NMR (126 MHz, $CDCl_3$)** δ 185.41, 146.78, 145.75, 134.74, 126.94, 124.16, 123.92, 47.47, 44.59, 40.43, 37.92, 36.89, 36.75, 33.49, 32.55, 30.04, 25.18, 24.02, 21.80, 18.57, 16.23.

**3-propylene oxide-1- dehydroabietic acid ester (1)**

A colorless oil liquid, yield 67.4%; **$^{1}$H NMR (400 MHz, $CDCl_3$)** δ 7.10 (d, *J* = 8.4 Hz, 1H, phenyl-H), 6.93 (d, *J* = 8.0 Hz, 1H, phenyl-H), 6.82 (s, 1H, phenyl-H), 4.32 (ddd, *J* = 31.6, 12.4, 6.4 Hz, 1H, 1/2$COOC\underline{H_2}CHCH_2$), 3.86 (ddd, *J* = 28.0, 12.3, 6.2 Hz, 1H, 1/2$COOC\underline{H_2}CHCH_2$), 3.11 (ddd, *J* = 10.0, 6.4, 3.2 Hz, 1H, $COOCH_2C\underline{H}CH_2$), 2.82 (m, 2H, phenyl-cyclohexane-$C\underline{H_2}$), 2.78 – 2.70 (m, 1H, 1/2$COOCH_2CHC\underline{H_2}$), 2.56 (dd, *J* = 4.8, 2.8 Hz, 1H, 1/2$COOCH_2CHC\underline{H_2}$), 2.22 (t, *J* = 12.4 Hz, 2H, $CC\underline{H}CH_2$+$C\underline{H}(CH_3)_2$), 1.84 – 1.53 (m, 6H, cyclohexane-3$C\underline{H_2}$), 1.48 – 1.31 (m, 2H, phenyl-cyclohexane-$C\underline{H_2}$), 1.22 (s, 3H, $CC\underline{H_3}$), 1.16 (s, 3H, $CC\underline{H_3}COO$), 1.14 (s, 6H, $CH(C\underline{H_3})_2$). **$^{13}$C NMR (101 MHz, $CDCl_3$)** δ 178.31, 146.74, 145.77, 134.68, 126.96, 124.22, 123.99, 65.13, 49.44, 47.83, 44.86, 44.64, 37.95, 36.98, 36.62, 33.49, 30.14, 25.23, 24.01, 21.83, 18.59, 16.53.

**3-((4-*N*-methylpiperazine) amino)-2-hydroxypropyl-1-dehydroabietic acid ester (2a)**

A colorless oil liquid, yield 57.0%; **$^{1}$H NMR (500 MHz, $CDCl_3$)** δ 7.14 (d, *J* = 8.0 Hz, 1H, phenyl-H), 6.97 (d, *J* = 6.0 Hz, 1H, phenyl-H), 6.85 (d, *J* = 5.0 Hz, 1H, phenyl-H),

5.08 – 4.94 (m, $CHO\underline{H}$), 4.19 – 3.93 (m, 1H, 1/2$COOC\underline{H_2}CHCH_2$), 3.93 – 3.84 (m, 1H, $COOCH_2C\underline{H}CH_2$), 3.77 – 3.65 (m, 1H, 1/2$COOC\underline{H_2}CHCH_2$), 2.86 (dd, *J* = 16.0, 3.5 Hz, 2H, phenyl-cyclohexane-$C\underline{H_2}$), 2.79 (dd, *J* = 13.8, 6.9 Hz, 1H, 1/2$COOCH_2CHC\underline{H_2}$), 2.68 – 2.54 (m, 3H, 1/2$COOCH_2CHC\underline{H_2}$ + $CC\underline{H}CH_2$+$C\underline{H}(CH_3)_2$), 2.37 (m, 4H, $\underline{2}(NC\underline{H_2}CH_2N)CH_3$), 2.31 – 2.18 (m, 7H, $\underline{2}(NC\underline{H_2}CH_2N)CH_3$ +$(CH_2)_2NC\underline{H_3}$), 1.80 – 1.59 (m, 6H, cyclohexane-3$C\underline{H_2}$), 1.51 – 1.38 (m, 2H, phenyl-cyclohexane-$C\underline{H_2}$), 1.27 – 1.22 (m, 3H, $CC\underline{H_3}$), 1.20 (s, 3H, $CC\underline{H_3}COO$), 1.19 (s, 6H, $CH(C\underline{H_3})_2$). **$^{13}$C NMR (126 MHz, $CDCl_3$)** δ 178.61, 146.91, 145.77, 134.74, 126.97, 124.28, 124.01, 69.28, 66.73, 66.60, 65.12, 60.61, 60.38, 55.17, 55.11, 47.88, 46.06, 44.92, 37.99, 37.03, 36.71, 33.53, 30.19, 25.35, 24.09, 21.84, 18.66, 16.64. **HRMS**: *m/z* $[M+H]^+$ calcd for $C_{28}H_{45}N_2O_3$:457.3425; found:457.3420.

**3-((4-*N*-ethylpiperazine) amino)-2-hydroxypropyl-1-dehydroabietic acid ester ester (2b)**

A colorless oil liquid, yield 75.1%; **$^1$H NMR (400 MHz, $CDCl_3$)** δ 7.16 (d, *J* = 8.4 Hz, 1H, phenyl-H), 7.00 (d, *J* = 8.4 Hz, 1H, phenyl-H), 6.87 (s, 1H, phenyl-H), 4.18 (dd, *J* = 11.4, 4.0 Hz, 1H, 1/4$COOC\underline{H_2}CHCH_2$), 4.13 – 4.04 (m, 1H, 1/2$COOC\underline{H_2}CHCH_2$), 4.00 (dd, *J* = 11.4, 5.6 Hz, 1H, 1/2$COOC\underline{H_2}CHCH_2$), 3.96 – 3.87 (m, 1H, $COOCH_2C\underline{H}CH_2$), 2.96 – 2.85 (m, 2H, phenyl-cyclohexane-$C\underline{H_2}$), 2.81 (dd, *J* = 14.0, 7.2 Hz, 1H, $COOCH_2CHC\underline{H_2}$), 2.74 – 2.68 (m, 1H, $COOCH_2CHC\underline{H_2}$), 2.73 – 2.56 (m, 2H, piperzine-$NC\underline{H_2}CH_3$), 2.53 – 2.35 (m, 8H, piperzine-8H), 2.34 – 2.20 (m, 2H, $CC\underline{H}CH_2$+$C\underline{H}(CH_3)_2$), 1.90 – 1.63 (m, 6H, cyclohexane-$C\underline{H_2}C\underline{H_2}C\underline{H_2}$), 1.55 – 1.40 (m, 2H, phenyl-cyclohexane-$C\underline{H_2}$), 1.30 – 1.25 (m, 3H, $CC\underline{H_3}$), 1.23 (s, 3H, $CC\underline{H_3}COO$),

1.21 (s, 6H, $CH(C\underline{H_3})_2$), 1.08 (t, *J* = 7.2 Hz, 3H, piperzine-$NCH_2C\underline{H_3}$). **$^{13}$C NMR (101 MHz, $CDCl_3$)** δ 178.51, 146.84, 145.69, 134.67, 126.89, 124.22, 123.94, 66.68, 66.55, 65.07, 60.39, 60.30, 52.81, 52.26, 47.80, 44.81, 37.94, 36.97, 36.65, 33.46, 30.14, 25.26, 24.02, 21.77, 18.60, 16.57, 11.98. **HRMS**: *m/z* $[M+H]^+$ calcd for $C_{29}H_{47}N_2O_3$:471.3581; found:471.3578.

**3-((4-*N*-tert butyl piperazine) amino-2-hydroxypropyl-1-dehydroabietic acid ester (2c)**

A white solid, yield 61.7%, m. p. 96.3-97.5 °C; **$^1$H NMR (500 MHz, $CDCl_3$)** δ 7.14 (d, *J* = 8.0 Hz, 1H, phenyl-H), 6.97 (d, *J* = 7.5 Hz, 1H, phenyl-H), 6.85 (s, 1H, phenyl-H), 4.19 – 3.94 (m, 2H, $COOC\underline{H_2}CHCH_2$), 3.89 (d, *J* = 4.0 Hz, 2H, $COOCH_2C\underline{H}CH_2$), 2.89 (d, *J* = 35.9 Hz, 2H, phenyl-cyclohexane-$C\underline{H_2}CH_2$), 2.79 (dt, *J* = 13.3, 6.8 Hz, 1H, 1/2$COOCH_2CHC\underline{H_2}$), 2.68 (s, 1H, 1/2$COOCH_2CHC\underline{H_2}$), 2.60 (d, *J* = 16.5 Hz, 4H, 2$C\underline{H_2}NC(CH_3)_3$), 2.49 – 2.33 (m, 4H, 2$C\underline{H_2}CH_2NC(CH_3)_3$), 2.25 (dd, *J* = 21.0, 12.0 Hz, 2H, $CC\underline{H}CH_2$ + $C\underline{H}(CH_3)_2$), 1.85 – 1.61 (m, 6H, cyclohexane-$C\underline{H_2}C\underline{H_2}C\underline{H_2}$), 1.45 (m, 2H, phenyl-cyclohexane-$CH_2C\underline{H_2}$), 1.26 (s, 3H, $CC\underline{H_3}CH_2$), 1.20 (s, 3H, $CC\underline{H_3}COO$), 1.19 (s, 6H, $CH(C\underline{H_3})_2$), 1.07 (s, 9H, $C(C\underline{H_3})_3$). **$^{13}$C NMR (126 MHz, $CDCl_3$)** δ 178.62, 146.91, 145.77, 134.75, 126.99, 124.28, 124.01, 66.76, 66.63, 65.12, 60.29, 60.22, 54.50, 53.64, 47.88, 45.77, 44.91, 44.86, 37.99, 37.03, 36.72, 33.53, 30.21, 30.18, 25.82, 25.34, 24.09, 21.84, 18.66, 16.63. **HRMS**: *m/z* $[M+H]^+$ calcd for $C_{31}H_{51}N_2O_3$:499.3894; found:499.3891.

**3-((4-*N*-phenylperazine) amino)-2-hydroxypropyl-1-dehydroabietic acid ester (2d)**

A white solid, yield 55.0%, m. p. 102.1-103.6 °C; **$^1$H NMR (400 MHz, $CDCl_3$)** δ 7.27

(dd, $J$ = 8.4, 7.6 Hz, 2H, piperazine-$NC(CH)_2\underline{(CH)}_2CH$), 7.17 (d, $J$ = 8.4 Hz, 1H, DAA-phenyl-H), 7.00 (d, $J$ = 8.4 Hz, 1H, DAA-phenyl-H), 6.93 (d, $J$ = 8.5 Hz, 2H, piperazine-$NC\underline{(CH)}_2(CH)_2CH$), 6.88 (t, $J$ = 6.0 Hz, 2H, DAA-phenyl-1H + piperazine-$NC(CH)_2(CH)_2\underline{CH}$), 4.21 (dd, $J$ = 11.2, 4.0 Hz, 1/2H, 1/4 $COOC\underline{H_2}CHCH_2$), 4.12 (m, 1H, 1/2H, 1/2$COOC\underline{H_2}CHCH_2$), 4.05 (dd, $J$ = 12.4, 6.8 Hz, 1/2H, 1/4$COOC\underline{H_2}CHCH_2$), 4.03 – 3.97 (m, 1H, $COOCH_2C\underline{H}CH_2$), 3.28– 3.19 (m, 4H, phenyl-$N(C\underline{H_2)_2}$), 2.93 – 2.78 (m, 4H, phenyl-$N(CH_2)_2(C\underline{H_2)_2}$), 2.68 – 2.59 (m, 2H, phenyl-cyclohexane-$C\underline{H_2}CH_2$), 2.54 – 2.44 (m, 2H, $COOCH_2CHC\underline{H_2}$), 2.35 – 2.20 (m, 2H, $CC\underline{H}CH_2$ + $C\underline{H}(CH_3)_2$), 1.85 – 1.64 (m, 6H, cyclohexane-$C\underline{H_2}C\underline{H_2}C\underline{H_2}$), 1.52 – 1.41 (m, 2H, phenyl-cyclohexane-$CH_2C\underline{H_2}$), 1.30 (s, 3H, $CC\underline{H_3}CH_2$), 1.23 (s, 3H, $CC\underline{H_3}COO$), 1.22 (d, $J$ = 1.6 Hz, 6H, $CH(C\underline{H_3)_2}$. **$^{13}$C NMR (101 MHz, $CDCl_3$)** δ 178.56, 151.03, 146.85, 145.77, 134.67, 129.19, 126.92, 124.22, 123.97, 120.08, 116.25, 77.36, 77.04, 76.73, 66.59, 66.47, 65.13, 60.50, 60.42, 53.30, 49.15, 47.85, 44.90, 37.94, 36.99, 36.69, 33.47, 30.14, 29.73, 25.26, 24.01, 21.80, 18.60, 16.58. **HRMS**: *m*/*z* $[M+H]^+$ calcd for $C_{33}H_{47}N_2O_3$:519.3581; found:519.3578.

**3-((4-*N*-2-fluorophenylperazine) amino)-2-hydroxypropyl-1-dehydroabietic acid ester (2e)**

A yellow solid, yield 60.9%, m. p. 58.5-59.8 °C; **$^1$H NMR (500 MHz, $CDCl_3$)** δ 7.19 (d, $J$ = 8.0 Hz, 1H, DAA-phenyl-H), 7.04 (m, 3H, DAA-phenyl-H + phenyl-NCCF$\underline{CH}$CH$\underline{CH}$CH), 6.98 – 6.92 (m, 2H, phenyl-NCCFCH$\underline{CH}$CH$\underline{CH}$), 6.90 (s, 1H, DAA-phenyl-H), 5.27 (s, 1H, OH), 4.23 (dd, $J$ = 11.4, 3.8 Hz, 1/2H, 1/4$COOC\underline{H_2}CHCH_2$), 4.17 – 4.10 (ddd, $J$ = 11.5, 6.0, 4.5 Hz, 1H, 1/2$COOC\underline{H_2}CHCH_2$),

4.05 (dd, $J$ = 11.0, 5.5 Hz, 1/2H, $1/4COOC\underline{H_2}CHCH_2$), 3.98 (ddd, $J$ = 13.0, 9.5, 5.0 Hz, 1H, $COOCH_2C\underline{H}CH_2$), 3.20 – 3.05 (m, 4H, phenyl-piperazine-$CFCN(C\underline{H_2})_2$), 2.96 – 2.86 (m, 2H, phenyl-cyclohexane-$C\underline{H_2}$), 2.85 – 2.76 (m, 2H, $COOCH_2CHC\underline{H_2}$), 2.66 – 2.56 (m, 2H, phenyl-piperazine-$CFCN(CH_2)_2C\underline{H_2}$), 2.52 – 2.44 (m, 2H, phenyl-piperazine-$CFCN(CH_2)_2C\underline{H_2}$), 2.35 – 2.25 (m, 2H, $CC\underline{H}CH_2$ + $C\underline{H}(CH_3)_2$), 1.97 – 1.58 (m, 6H, cyclohexane-$C\underline{H_2}C\underline{H_2}C\underline{H_2}$ ), 1.52 – 1.43 (m, 2H, phenyl-cyclohexane-$C\underline{H_2}$), 1.30 (d, $J$ = 8.6 Hz, 3H, $CC\underline{H_3}CH_2$), 1.24 (s, 3H, $CC\underline{H_3}COO$), 1.23 (s, 6H, $CH(C\underline{H_3})_2$). **$^{13}$C NMR (126 MHz, $CDCl_3$)** δ 178.65, 156.79, 154.83, 146.87, 145.83, 140.05 (d, $^1J_{C-F}$ = 8.6 Hz), 134.78, 127.03, 124.62, (d, $^2J_{C-F}$ = 3.2 Hz), 124.33 (d, $^3J_{C-F}$ = 32.0 Hz), 122.77 (d, $^4J_{C-F}$ = 7.9 Hz), 119.06 (d, $^5J_{C-F}$ = 2.4 Hz), 116.34 (d, $^6J_{C-F}$ = 20.5 Hz), 66.75, 66.63, 65.23, 60.53, 60.45, 53.43, 50.68, 47.93, 44.98, 38.04, 37.08, 36.77, 33.58, 30.24, 25.39, 24.14, 21.90, 18.71, 16.70. **$^{19}$F NMR (376 MHz, DMSO-*$d_6$*)** δ -122.78. **HRMS**: *m/z* $[M+H]^+$ calcd for $C_{33}H_{46}N_2O_3F$:537.3487; found:537.3485.

**3-((4-*N*-4-fluorophenylperazine) amino)-2-hydroxypropyl-1-dehydroabietic acid ester (2f)**

A pink solid, yield 83.5%, m. p. 90.4-91.3 °C; **$^1$H NMR (400 MHz, DMSO-*$d_6$*)** δ 7.15 (d, $J$ = 8.4 Hz, 1H, DAA-phenyl-H), 7.02 (t, $J$ = 8.8 Hz, 2H, piperazine-$NC(CH\underline{CH})_2CF$), 6.96 (d, $J$ = 8.1 Hz, 1H, DAA-phenyl-H), 6.94 – 6.87 (m, 2H, piperazine-$NC\underline{(CH)}_2(CH)_2CF$), 6.83 (s, 1H, DAA-phenyl-H), 4.85 (s, 1H, OH), 4.09 (dd, $J$ = 10.8, 4.0 Hz, 1/2H, $1/4COOC\underline{H_2}CHCH_2$), 4.04 – 3.96 (m, 1H, $1/2COOC\underline{H_2}CHCH_2$), 3.92 (dd, $J$ = 10.8, 6.0 Hz, 1/2H, $1/4COOC\underline{H_2}CHCH_2$), 3.86 (s, 1H, $COOCH_2C\underline{H}CH_2$), 3.15 – 2.95 (m, 4H, phenyl-piperazine-$N(C\underline{H_2})_2(CH_2)_2$), 2.83 –

2.70 (m, 2H, $COOCH_2CHC\underline{H_2}$), 2.63 – 2.51 (m, 4H, phenyl-piperazine-$N(CH_2)_2(C\underline{H_2})_2$), 2.43 – 2.33 (m, 2H, phenyl-cyclohexane-$C\underline{H_2}$), 2.26 (d, $J$ = 12.4 Hz, 1H, $C\underline{H}(CH_3)_2$), 2.11 (d, $J$ = 12.4 Hz, 1H, $CC\underline{H}CH_2$), 1.84 – 1.47 (m, 6H, cyclohexane-$C\underline{H_2}C\underline{H_2}C\underline{H_2}$), 1.40 – 1.26 (m, 2H, phenyl-cyclohexane-$C\underline{H_2}$), 1.20 (s, 3H, $CC\underline{H_3}CH_2$), 1.15 (s, 3H, $CC\underline{H_3}COO$), 1.13 (d, $J$ = 3.9 Hz, 6H, $CH(C\underline{H_3})_2$). **$^{13}$C NMR (101 MHz, DMSO-*d$_6$*)** δ 177.98, 177.97, 157.60, 155.26, 148.38, 147.09, 145.54, 134.62, 126.91, 124.56, 124.20, 117.51 ($^1J_{C\text{-}F}$ = 7.5 Hz), 115.78, 115.56, 66.38, 61.49, 53.86, 49.45, 47.54, 45.19, 45.07, 38.13, 36.97, 36.57, 33.36, 30.01, 25.38, 24.41, 24.36, 21.63, 18.59, 16.82, 16.80. **$^{19}$F NMR (376 MHz, DMSO-*d$_6$*)** δ -125.70. **HRMS**: *m/z* $[M+H]^+$ calcd for $C_{33}H_{46}N_2O_3F$:537.3487; found:537.3485.

**3-((4-*N*-3-chloro-5-trifluoro-2-pyridinylperazine) amino)-2-hydroxypropyl-1-dehydroabietic acid ester (2g)**

A yellow oil liquid, yield 63.9%; **$^1$H NMR (500 MHz, $CDCl_3$)** δ 8.38 (s, 1H, pyridine-H), 7.75 (d, $J$ = 2.0 Hz, 1H, pyridine-H), 7.17 (d, $J$ = 8.0 Hz, 1H, phenyl-H), 6.99 (d, $J$ = 8.0 Hz, 1H, phenyl-H), 6.88 (s, 1H, phenyl-H), 5.28 (s, 1H, OH), 4.23 – 4.02 (m, 2H, $COOC\underline{H_2}CHCH_2$), 3.97 (ddd, $J$ = 13.0, 9.0, 4.5 Hz, 1H, $COOCH_2C\underline{H}CH_2$), 3.52 (s, 4H, pyridine-2$NC\underline{H_2}CH_2$), 2.93 – 2.85 (m, 2H, phenyl-cyclohexane-$C\underline{H_2}CH_2$), 2.84 – 2.76 (m, 2H, $COOCH_2CHC\underline{H_2}$), 2.62 – 2.53 (m, 2H, pyridine-NCH2$C\underline{H_2}N$), 2.52 – 2.42 (m, 2H, pyridine-NCH2$C\underline{H_2}N$), 2.35 – 2.22 (m, 2H, $CC\underline{H}CH_2$ + $C\underline{H}(CH_3)_2$), 1.87 – 1.63 (m, 6H, cyclohexane-3$C\underline{H_2}$), 1.55 – 1.41 (m, phenyl-cyclohexane-$CH_2C\underline{H_2}$), 1.29 (s, 3H, $CC\underline{H_3}CH_2$), 1.22 (s, 3H, $CC\underline{H_3}COO$), 1.21 (d, $J$ = 2.5 Hz, 6H, $CH(C\underline{H_3})_2$). **$^{13}$C NMR (126 MHz, $CDCl_3$)** δ 178.63, 159.79, 146.93, 145.83, 143.09, 143.06, 136.12, 136.10,

134.74, 127.00, 124.30, 124.06, 120.93, 66.70, 66.57, 65.26, 60.57, 60.49, 53.12, 48.61, 47.92, 44.97, 44.92, 38.01, 37.06, 36.74, 33.55, 30.23, 25.34, 24.10, 21.87, 18.68, 16.66. **$^{19}$F NMR (376 MHz, CDCl$_3$)** δ -61.39. **HRMS**: *m/z* $[M+H]^+$ calcd for $C_{33}H_{44}N_3O_3F_3Cl$:622.3018; found:622.3013.

**3-((4-*N*-4-fluorobenzylperazine) amino)-2-hydroxypropyl-1-dehydroabietic acid ester (2h)**

A colorless oil liquid, yield 75.3%; **$^1$H NMR (400 MHz, CDCl$_3$)** δ 7.11 (dd, *J* = 8.4, 5.6 Hz, 2H, piperazine-NCH$_2$C($\underline{CH}$)$_2$(CH)$_2$CF), 7.01 (d, *J* = 8.4 Hz, 1H, DAA-phenyl-H), 6.87 – 6.80 (m, 3H, piperazine-NCH$_2$C(CH)$_2$($\underline{CH}$)$_2$CF + DAA-phenyl-1H), 6.72 (s, 1H, DAA-phenyl-H), 5.12 (s, 1H, OH), 4.03 (dd, *J* = 11.2, 3.6 Hz, 1/2H, 1/4COOC$\underline{H_2}$CHCH$_2$), 3.97 – 3.88 (m, 1H, 1/2COOC$\underline{H_2}$CHCH$_2$), 3.84 (dd, *J* = 11.4, 5.7 Hz, 1/2H, 1/4COOC$\underline{H_2}$CHCH$_2$), 3.74 (m, 1H, COOCH$_2$C$\underline{H}$CH$_2$), 2.78 – 2.69 (m, 2H, phenyl-cyclohexane-C$\underline{H_2}$CH$_2$), 2.68 – 2.60 (m, 1H, 1/2COOCH$_2$CHC$\underline{H_2}$), 2.51 (s, 1H, 1/2COOCH$_2$CHC$\underline{H_2}$), 2.35 – 2.18 (m, 8H, piperazine-8H), 2.17 – 2.06 (m, 2H, CC$\underline{H}$CH$_2$ + C$\underline{H}$(CH$_3$)$_2$), 1.38 – 1.25 (m, 6H, cyclohexane-C$\underline{H_2}$C$\underline{H_2}$C$\underline{H_2}$), 1.38 – 1.25 (m, 2H, phenyl-cyclohexane-C$\underline{H_2}$), 1.12 (d, *J* = 9.2 Hz, 3H, CC$\underline{H_3}$CH$_2$), 1.07 (s, 3H, CC$\underline{H_3}$COO), 1.05 (s, 6H, CH(C$\underline{H_3}$)$_2$). **$^{13}$C NMR (101 MHz, CDCl$_3$)** δ 178.55, 163.25, 160.82, 146.88, 145.73, 134.70, 133.70, 130.68 (*d*, $^1J_{C\text{-}F}$ = 7.8), 126.93 (*d*, $^2J_{C\text{-}F}$ = 2.3), 124.22, 123.97, 115.17, 114.96, 66.69, 66.55, 65.05, 62.16, 60.29, 60.22, 53.02, 47.82, 44.83, 37.95, 36.99, 36.67, 33.48, 30.15, 25.28, 24.03, 21.79, 18.61, 16.59. **$^{19}$F NMR (376 MHz, CDCl$_3$)** δ -115.67. **HRMS**: *m/z* $[M+H]^+$ calcd for $C_{34}H_{48}N_2O_3F$:551.3646; found:551.3639.

**3-piperidine-2-hydroxypropyl-1-dehydroabietic acid ester (2i)**

A yellowish solid, yield 81.6%, m. p. 61.7-62.9 °C; **$^1$H NMR (500 MHz, $CDCl_3$)** δ 7.16 (d, *J* = 8.1 Hz, 1H, phenyl-H), 6.99 (d, *J* = 7.6 Hz, 1H, phenyl-H), 6.88 (s, 1H, phenyl-H), 4.19 – 3.97 (m, 2H, $COOC\underline{H_2}CHCH_2$), 3.92 (s, 1H, $COOCH_2C\underline{H}CH_2$), 3.00 – 2.85 (m, 2H, phenyl-cyclohexane-$C\underline{H_2}$), 2.82 (m, 1H, 1/2 $COOCH_2CHC\underline{H_2}$), 2.60 (s, 1H, 1/2 $COOCH_2CHC\underline{H_2}$), 2.42 – 2.30 (m, 4H, piperidine-$CH_2N(C\underline{H_2)_2}$), 2.30 – 2.19 (m, 2H, $CC\underline{H}CH_2$ + $C\underline{H}(CH_3)_2$), 1.89 – 1.63 (m, 6H, cyclohexane-$C\underline{H_2}C\underline{H_2}C\underline{H_2}$), 1.55 (d, *J* = 22.5 Hz, 4H, piperidine-$CH_2N(CH_2C\underline{H_2)_2}$), 1.47 (d, *J* = 29.6 Hz, 4H = 2H + 2H, phenyl-cyclohexane-$C\underline{H_2}$ + piperidine-$N(CH_2CH_2)_2C\underline{H_2}$), 1.27 (d, *J* = 13.8 Hz, 3H, $C\underline{H_3}CH_2$), 1.22 (s, 3H, $C\underline{H_3}COO$), 1.21 (s, 6H, $CH(C\underline{H_3)_2}$). **$^{13}$C NMR (126 MHz, $CDCl_3$)** δ 178.59, 146.96, 145.76, 134.81, 126.99, 124.29, 124.00, 66.87, 66.72, 64.93, 61.10, 61.03, 54.78, 47.87, 44.95, 44.90, 38.03, 37.06, 36.73, 33.56, 30.21, 26.02, 25.36, 24.18, 24.12, 21.86, 18.70, 16.66. **HRMS**: *m/z* $[M+H]^+$ calcd for $C_{28}H_{44}NO_3$:442.3316; found:442.3313.

**3-(2-methyl) piperidine-2-hydroxypropyl-1-dehydroabietic acid ester (2j)**

A white oil liquid, yield 49.6%; **$^1$H NMR (500 MHz, $CDCl_3$)** δ 7.15 (d, *J* = 8.0 Hz, 1H, phenyl-H), 6.98 (d, *J* = 8.0 Hz, 1H, phenyl-H), 6.87 (s, 1H, phenyl-H), 4.17 – 3.97 (m, 2H, $COOC\underline{H_2}CHCH_2$), 3.94 – 3.86 (m, 1H, $COOCH_2C\underline{H}CH_2$), 3.86 – 3.67 (m, 1H, piperidine-$NC\underline{H}CH_3$), 3.00 – 2.85 (m, 2H, phenyl-cyclohexane-$C\underline{H_2}$), 2.84 – 2.64 (m, 2H, $COOCH_2CHC\underline{H_2}$), 2.50 – 2.34 (m, 1H, piperidine-1/2$C\underline{H_2}$), 2.35 – 2.22 (m, 2H, $CC\underline{H}CH_2$ + $C\underline{H}(CH_3)_2$ ), 2.20 – 2.04 (m, 1H, piperidine-1/2$C\underline{H_2}$), 1.87 – 1.72 (m, 4H, piperidine-2$C\underline{H_2}$), 1.70 – 1.55 (m, 6H, cyclohexane-$C\underline{H_2}C\underline{H_2}C\underline{H_2}$), 1.53 – 1.43 (m, 2H,

phenyl-cyclohexane-$C\underline{H_2}$), 1.28 (s, 3H, $CC\underline{H_3}CH_2$), 1.21 (s, 3H, $CC\underline{H_3}COO$), 1.20 (s, 6H, $CH(C\underline{H_3})_2$), 1.05 – 0.99 (m, 3H, piperidine-$NCHC\underline{H_3}$). **$^{13}$C NMR (126 MHz, $CDCl_3$)** δ 178.58, 146.94, 145.75, 134.79, 126.99, 124.25, 123.99, 66.69, 66.01, 64.65, 57.00, 56.26, 55.24, 52.58, 47.86, 44.94, 38.05, 37.03, 36.76, 34.65, 33.56, 32.95, 30.18, 25.99, 25.30, 24.10, 23.79, 21.84, 18.68, 16.64. **HRMS**: *m/z* $[M+H]^+$ calcd for $C_{29}H_{46}NO_3$:456.3472; found:456.3466.

**3-(3-methyl) piperidine-2-hydroxypropyl-1-dehydroabietic acid ester (2k)**

A colorless oil liquid, yield 68.4%; **$^1$H NMR (500 MHz, $CDCl_3$)** δ 7.16 (d, *J* = 8.0 Hz, 1H, phenyl-H), 6.99 (d, *J* = 7.5 Hz, 1H, phenyl-H), 6.87 (s, 1H, phenyl-H), 4.19 – 3.97 (m, 2H, $COOC\underline{H_2}CHCH_2$), 3.94 (m, 1H, $COOCH_2C\underline{H}CH_2$), 3.88 – 3.67 (m, 1H, piperidine-$(CH_2)_2C\underline{H}CH_3$), 2.99 – 2.86 (m, 2H, phenyl-cyclohexane-$C\underline{H_2}$), 2.85 – 2.56 (m, 2H, $COOCH_2CHC\underline{H_2}$), 2.49 – 2.32 (m, 2H, piperidine-$C\underline{H_2}$), 2.30 – 2.17 (m, 2H, $CC\underline{H}CH_2$ + $C\underline{H}(CH_3)_2$), 1.95 – 1.70 (m, 6H, piperidine-3$C\underline{H_2}$), 1.69 – 1.51 (m, 6H, cyclohexane-$C\underline{H_2}C\underline{H_2}C\underline{H_2}$), 1.51 – 1.37 (m, 2H, phenyl-cyclohexane-$C\underline{H_2}$), 1.27 (d, *J* = 13.6 Hz, 3H, $CC\underline{H_3}CH_2$), 1.22 (s, 3H, $CC\underline{H_3}COO$), 1.21 (d, *J* = 15.0 Hz, 6H, $CH(C\underline{H_3})_2$), 0.85 (d, *J* = 6.5 Hz, 3H, piperidine-$(CH_2)_2CHC\underline{H_3}$). **$^{13}$C NMR (126 MHz, $CDCl_3$)** δ 178.61, 147.10, 145.78, 134.80, 127.00, 124.30, 124.01, 66.73, 64.97, 64.91, 60.57, 55.65, 52.83, 47.88, 44.90, 38.01, 37.06, 36.73, 33.56, 32.69, 31.09, 30.21, 25.37, 24.11, 21.86, 19.63, 19.58, 18.69, 16.65. **HRMS**: *m/z* $[M+H]^+$ calcd for $C_{29}H_{46}NO_3$:456.3472; found:456.3468.

**3-(4-methyl) piperidine-2-hydroxypropyl-1-dehydroabietic acid ester (2l)**

A yellowish solid, yield 94.9%, m. p. 58.9-60.2 °C; **$^1$H NMR (500 MHz, $CDCl_3$)** δ

7.16 (d, *J* = 8.0 Hz, 1H, phenyl-H), 6.99 (d, *J* = 8.0 Hz, 1H, phenyl-H), 6.87 (s, 1H, phenyl-H), 5.05 – 4.97 (m, 1H, OH), 4.20 – 3.96 (m, 2H, $COOC\underline{H_2}CHCH_2$), 3.91 (td, *J* = 9.5, 4.9 Hz, 1H, $COOCH_2C\underline{H}CH_2$), 2.96 – 2.86 (m, 2H, phenyl-cyclohexane-$C\underline{H_2}CH_2$), 2.85 – 2.73 (m, 2H, $COOCH_2CHC\underline{H_2}$), 2.40 – 2.31 (m, 2H, piperidine-$C\underline{H_2}$), 2.30 – 2.23 (m, 2H, $CC\underline{H}CH_2$ + $C\underline{H}(CH_3)_2$), 2.04 – 1.90 (m, 1H, piperidine-$(CH_2)_2C\underline{H}CH_3$), 1.85 – 1.58 (m, 8H = 6H+2H, cyclohexane-$C\underline{H_2}C\underline{H_2}C\underline{H_2}$ + piperidine-$C\underline{H_2}$), 1.55 – 1.40 (m, 2H, phenyl-cyclohexane-$C\underline{H_2}$), 1.28 (d, *J* = 14.0 Hz, 3H, $CC\underline{H_3}CH_2$), 1.22 (s, 3H, $CC\underline{H_3}COO$), 1.21 (s, 6H, $CH(C\underline{H_3})_2$), 0.91 (d, *J* = 6.4 Hz, 3H, piperidine-$(CH_2)_2CHC\underline{H_3}$). **$^{13}$C NMR (126 MHz, $CDCl_3$)** δ 178.60, 146.95, 145.76, 134.81, 127.00, 124.29, 124.00, 67.10, 66.88, 66.73, 65.04, 60.71, 55.74, 52.65, 47.87, 44.90, 38.02, 37.05, 36.72, 34.51, 34.17, 33.56, 30.66, 30.21, 25.36, 24.11, 21.92, 18.69, 16.65. **HRMS**: *m/z* $[M+H]^+$ calcd for $C_{29}H_{46}NO_3$:456.3472; found:456.3467.

**3-(4-Bromi) piperidine-2-hydroxypropyl-1-dehydroabietic acid ester (2m)**

A white solid, yield 40.1%, m. p. 83.6-85.1 °C; **$^1$H NMR (500 MHz, $CDCl_3$)** δ 7.17 (d, *J* = 8.0 Hz, 1H, phenyl-H), 7.00 (d, *J* = 8.0 Hz, 1H, phenyl-H), 6.88 (s, 1H, phenyl-H), 4.27 – 3.97 (m, 2H, $COOC\underline{H_2}CHCH_2$), 3.90 (m, 1H, $COOCH_2C\underline{H}CH_2$), 3.36 (s, 1H, $C\underline{H}$-Br), 2.95 – 2.77 (m, 4H, phenyl-cyclohexane-$C\underline{H_2}$ + $COOCH_2CHC\underline{H_2}$), 2.73 – 2.44 (m, 2H, piperidine-$CH_2NC\underline{H_2}CH_2$), 2.43 – 2.33 (m, 2H, piperidine-$CH_2NC\underline{H_2}CH_2$), 2.32– 2.24 (m, 2H, $CC\underline{H}CH_2$ + $C\underline{H}(CH_3)_2$), 2.20 – 2.10 (m, 2H, piperidine-$CH_2NCH_2C\underline{H_2}$), 2.07– 1.95 (m, 2H, piperidine-$CH_2NCH_2C\underline{H}$), 1.90 – 1.58 (m, 6H, cyclohexane-$C\underline{H_2}C\underline{H_2}C\underline{H_2}$), 1.55 – 1.37 (m, 2H, phenyl-cyclohexane-$C\underline{H_2}$), 1.29 – 1.25 (s, 3H, $CC\underline{H_3}CH_2$), 1.22 (s, 3H, $CC\underline{H_3}COO$), 1.21 (s, 6H, $CH(C\underline{H_3})_2$). **$^{13}$C NMR (126**

**MHz, $CDCl_3$)** δ 178.62, 146.93, 145.82, 134.75, 127.01, 124.31, 124.05, 66.70, 66.57, 65.31, 60.37, 60.30, 47.90, 44.96, 44.91, 38.02, 37.06, 36.74, 36.20, 33.56, 30.22, 25.36, 24.13, 21.87, 18.69, 16.67. **HRMS**: *m/z* $[M+H]^+$ calcd for $C_{28}H_{43}NO_3Br$:520.2421; found:520.2419.

**3-(4-chloro) piperidine-2-hydroxypropyl-1-dehydroabietic acid ester (2n)**

A white solid, yield 44.7%, m. p. 99.2-100.5 °C; **$^1$H NMR (500 MHz, $CDCl_3$)** δ 7.16 (d, *J* = 8.5 Hz, 1H, phenyl-H), 6.99 (d, *J* = 8.0 Hz, 1H, phenyl-H), 6.87 (s, 1H, phenyl-H), 4.16 (dd, *J* = 11.5, 4.0 Hz, 1/2H, 1/2C<u>H</u>-Br), 4.07 (ddd, *J* = 19.5, 11.5, 4.0 Hz, 2H, COOC<u>$H_2$</u>CHC$H_2$), 3.99 (dd, *J* = 11.5, 6.0 Hz, 1/2H, 1/2C<u>H</u>-Br), 3.91 – 3.86 (m, 1H, COOC$H_2$C<u>H</u>C$H_2$), 2.94 – 2.77 (m, 4H, phenyl-cyclohexane-C<u>$H_2$</u> + COOC$H_2$CHC<u>$H_2$</u>), 2.73 – 2.62 (m, 1H, piperidine-1/2C$H_2$), 2.54 – 2.45 (m, 1H, piperidine-1/2C$H_2$), 2.41 – 2.34 (m, 2H, cyclohexane-C<u>$H_2$</u>), 2.32 – 2.20 (m, 2H, CC<u>H</u>C$H_2$ + C<u>H</u>($CH_3$)$_2$), 2.15 – 2.03 (m, 2H, piperidine-C$H_2$NC$H_2$C<u>$H_2$</u>), 1.95 – 1.85 (m, 2H, piperidine-C$H_2$NC$H_2$C<u>$H_2$</u>), 1.80 – 1.55 (m, 6H, cyclohexane-C<u>$H_2$</u>C<u>$H_2$</u>C<u>$H_2$</u>), 1.55 – 1.40 (m, 2H, phenyl-cyclohexane-C<u>$H_2$</u>), 1.28 – 1.24 (m, 3H, CC<u>$H_3$</u>C$H_2$), 1.21 (s, 3H, CC<u>$H_3$</u>COO), 1.20 (s, 6H, CH(C<u>$H_3$</u>)$_2$). **$^{13}$C NMR (126 MHz, $CDCl_3$)** δ 178.63, 146.98, 145.81, 134.75, 126.98, 124.29, 124.04, 66.69, 66.56, 65.30, 60.29, 60.22, 56.85, 50.98, 47.90, 44.95, 44.89, 38.00, 37.05, 36.72, 35.43, 33.54, 30.21, 25.34, 24.10, 21.85, 18.67, 16.64. **HRMS**: *m/z* $[M+H]^+$ calcd for $C_{28}H_{43}NO_3Cl$:476.2926; found:476.2925.

**3-(4-hydroxyl) piperidine-2-hydroxypropyl-1-dehydroabietic acid ester (2o)**

A colorless oil liquid, yield 41.7%; **$^1$H NMR (500 MHz, $CDCl_3$)** δ 7.15 (d, *J* = 8.0 Hz, 1H, phenyl-H), 6.98 (d, *J* = 8.0 Hz, 1H, phenyl-H), 6.86 (s, 1H, phenyl-H), 4.16 (dd,

1/2H, 1/4COOC$\underline{H}_2$CHCH$_2$), 4.07 (dd, 1H, 1/2COOC$\underline{H}_2$CHCH$_2$), 4.01 – 3.95 (dd, 1/2H, 1/4COOC$\underline{H}_2$CHCH$_2$), 3.93 – 3.85 (m, 1H, COOCH$_2$C$\underline{H}$CH$_2$), 3.71 (s, 1H, piperidine-C$\underline{H}$OH), 2.94 – 2.77 (m, 4H, phenyl-cyclohexane-C$\underline{H}_2$ + COOCH$_2$CHC$\underline{H}_2$), 2.68 – 2.58 (m, 1H, piperidine-1/2CH$_2$), 2.44 – 2.35 (m, 2H, phenyl-cyclohexane-C$\underline{H}_2$), 2.32 – 2.20 (m, 2H, CC$\underline{H}$CH$_2$ + C$\underline{H}$(CH$_3$)$_2$), 2.16 (s, 1H, piperidine-1/2CH$_2$), 1.92 – 1.75 (m, 4H, piperidine-2CH$_2$), 1.74 – 1.50 (m, 6H, cyclohexane-C$\underline{H}_2$C$\underline{H}_2$C$\underline{H}_2$), 1.48 – 1.38 (m, 2H, phenyl-cyclohexane-C$\underline{H}_2$), 1.26 (d, *J* = 12.2 Hz, 3H, CC$\underline{H}_3$CH$_2$), 1.21 (s, 3H, CC$\underline{H}_3$COO), 1.20 (s, 6H, CH(C$\underline{H}_3$)$_2$). **$^{13}$C NMR (126 MHz, CDCl$_3$)** δ 178.68, 146.93, 145.81, 134.77, 126.99, 124.29, 124.02, 66.75, 66.62, 65.29, 60.28, 60.21, 50.48, 47.89, 44.94, 44.89, 37.99, 37.04, 36.72, 34.51, 34.34, 33.54, 30.18, 25.34, 24.10, 21.84, 18.67, 16.64. **HRMS**: *m*/*z* [M+H]$^+$ calcd for $C_{28}H_{44}NO_4$:458.3265; found:458.3260.

**3-*N*-morpholine-2-hydroxypropyl-1-dehydroabietic acid ester (2p)**

A yellowish solid, yield 60.7%, m. p.74.8-75.9 °C; **$^1$H NMR (500 MHz, CDCl$_3$)** δ 7.16 (d, *J* = 8.0 Hz, phenyl-H), 6.99 (d, *J* = 8.0 Hz, phenyl-H), 6.87 (s, 1H, phenyl-H), 4.18 (dd, *J* = 11.5, 3.5 Hz, 1/2H, 1/4COOC$\underline{H}_2$CHCH$_2$), 4.13 – 4.04 (m, 1H, 1/2COOC$\underline{H}_2$CHCH$_2$), 4.00 (dd, *J* = 11.4, 5.7 Hz, 1/2H, 1/4COOC$\underline{H}_2$CHCH$_2$), 3.96 – 3.89 (m, 1H, COOCH$_2$C$\underline{H}$CH$_2$), 3.75 – 3.65 (m, 4H, morpholine-N(CH$_2$)$_2$(C$\underline{H}_2$)$_2$), 2.90 – 2.85 (m, 2H, phenyl-cyclohexane-C$\underline{H}_2$), 2.81 (dt, *J* = 13.8, 7.0 Hz, 1H, 1/2COOCH$_2$CHC$\underline{H}_2$), 2.62 (dd, *J* = 10.4, 5.5 Hz, 1H, 1/2COOCH$_2$CHC$\underline{H}_2$), 2.45 – 2.35 (m, 4H, morpholine-N(CH$_2$)$_2$(C$\underline{H}_2$)$_2$), 2.27 (dd, *J* = 23.5, 12.0 Hz, 2H, CC$\underline{H}$CH$_2$ + C$\underline{H}$(CH$_3$)$_2$), 1.87 – 1.63 ((m, 6H, cyclohexane-C$\underline{H}_2$C$\underline{H}_2$C$\underline{H}_2$), 1.53 – 1.39 (m, 2H, phenyl-cyclohexane-C$\underline{H}_2$), 1.27 (s, 3H, CC$\underline{H}_3$CH$_2$), 1.21 (s, 3H, CC$\underline{H}_3$COO), 1.20 (s,

6H, CH(C$\underline{H_3}$)$_2$). **$^{13}$C NMR (126 MHz, CDCl$_3$)** δ 178.63, 146.91, 145.83, 134.73, 126.98, 124.30, 124.05, 67.03, 66.66, 66.54, 65.04, 60.99, 60.91, 53.75, 47.90, 44.94, 38.00, 37.05, 36.73, 33.55, 30.19, 25.34, 24.10, 21.85, 18.67, 16.65. **HRMS**: *m*/*z* [M+H]$^+$ calcd for $C_{27}H_{42}NO_4$:444.3108; found:444.3104.

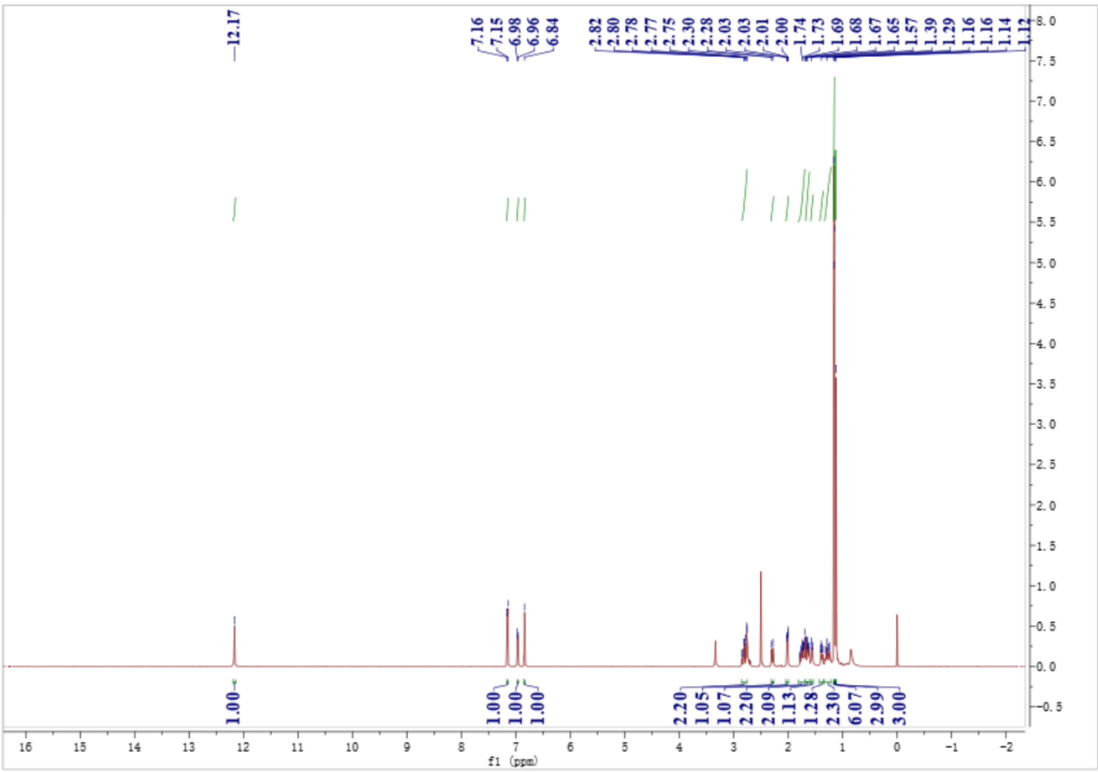

Figure S1. $^1$H NMR Spectrum (DMSO-$d_6$, 500 MHz) of DAA.

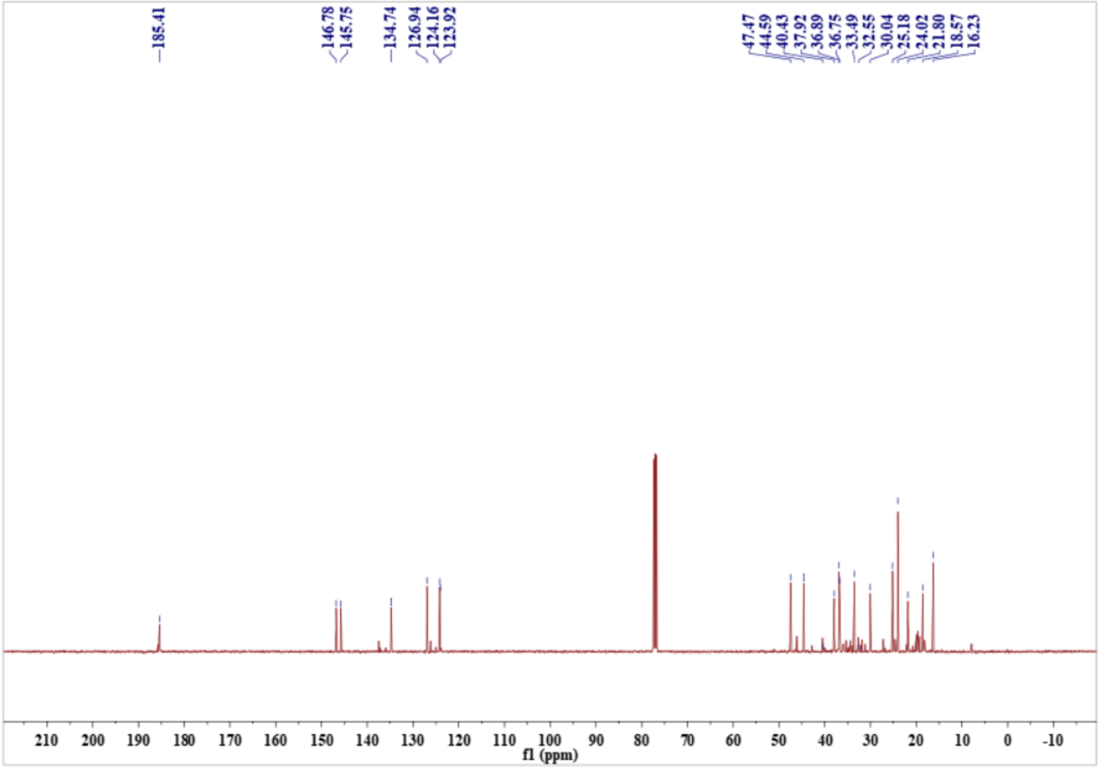

Figure S2. $^{13}$C NMR Spectrum ($CDCl_3$, 126 MHz) of DAA.

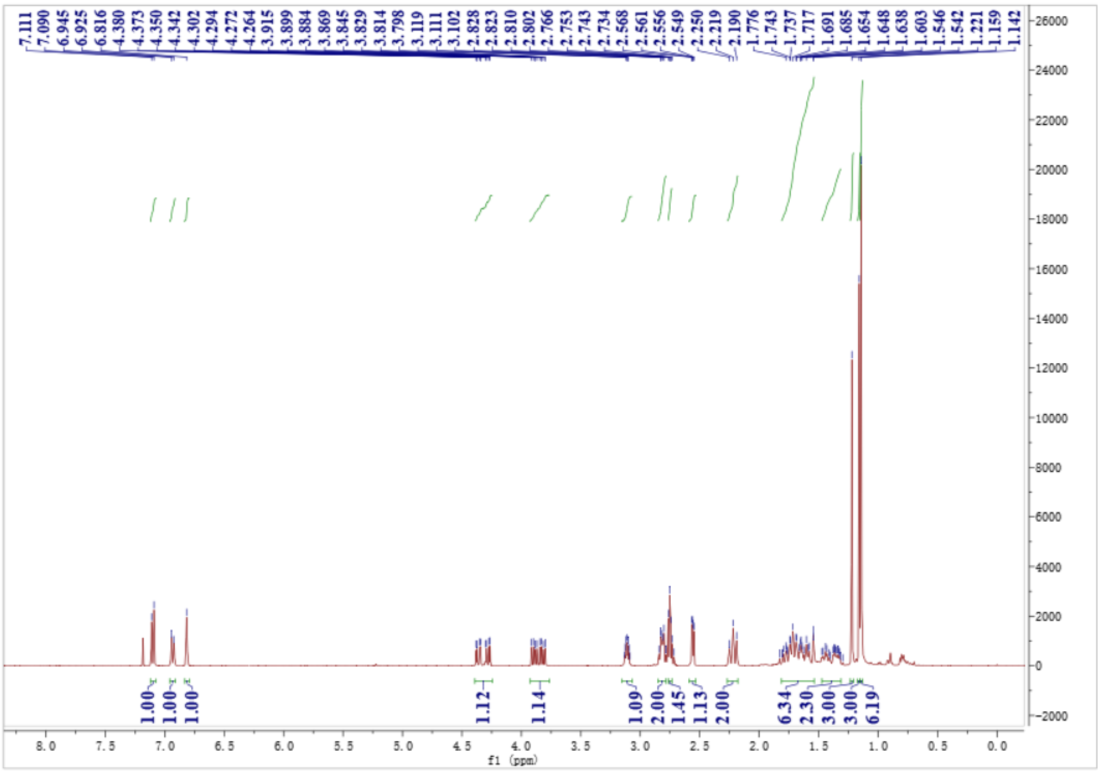

Figure S3. $^{1}$H NMR Spectrum ($CDCl_3$, 400 MHz) of 1.

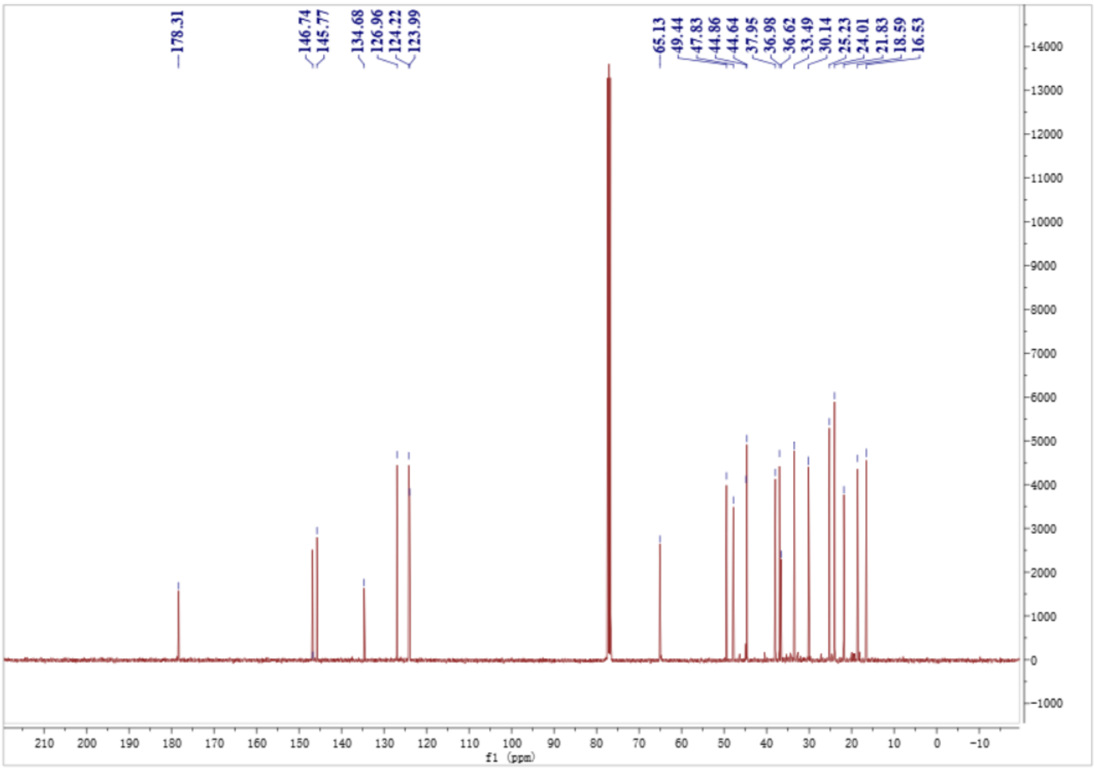

Figure S4. $^{13}$C NMR Spectrum ($CDCl_3$, 101 MHz) of 1.

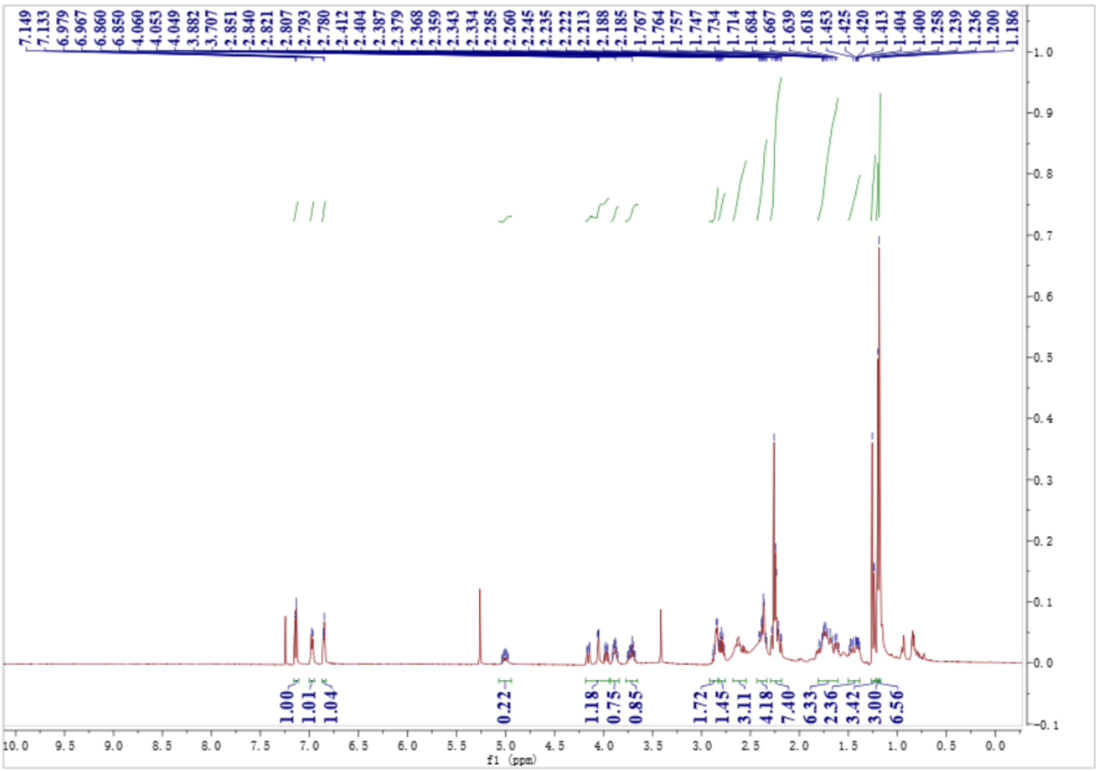

Figure S5. $^{1}$H NMR Spectrum ($CDCl_3$, 500 MHz) of 2a.

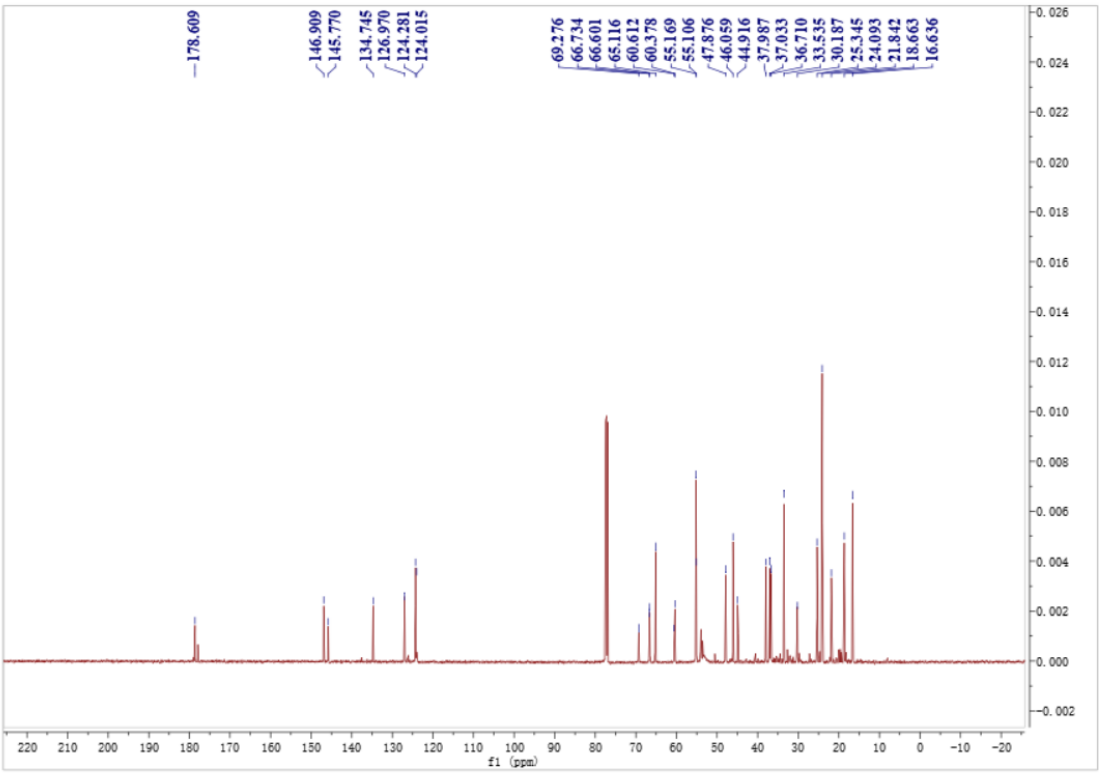


Figure S6. $^{13}$C NMR Spectrum ($CDCl_3$, 126 MHz) of 2a.

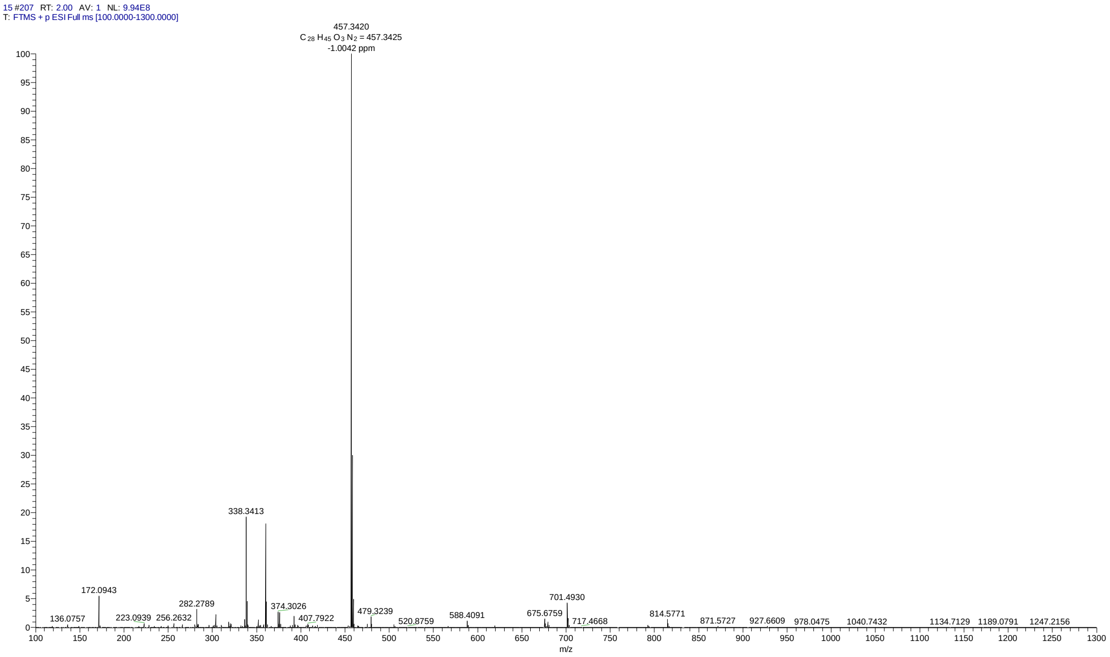


Figure S7. HRMS Spectrum of 2a.

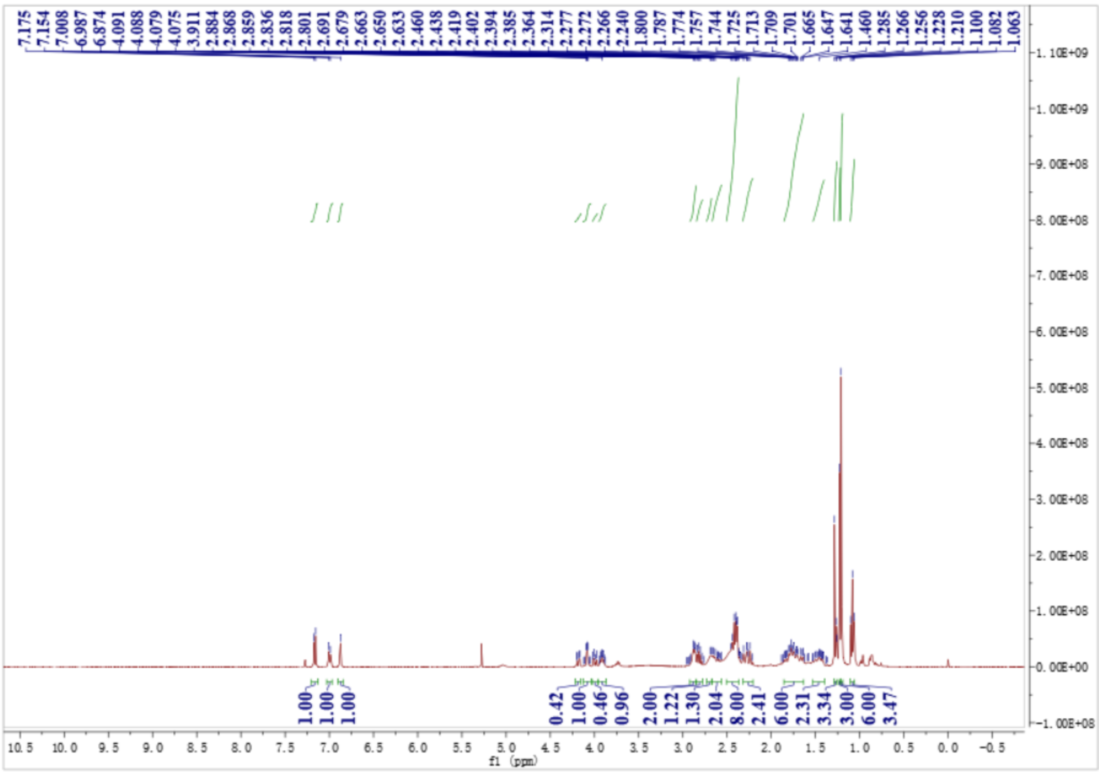


Figure S8. $^{1}H$ NMR Spectrum ($CDCl_3$, 400 MHz) of 2b.

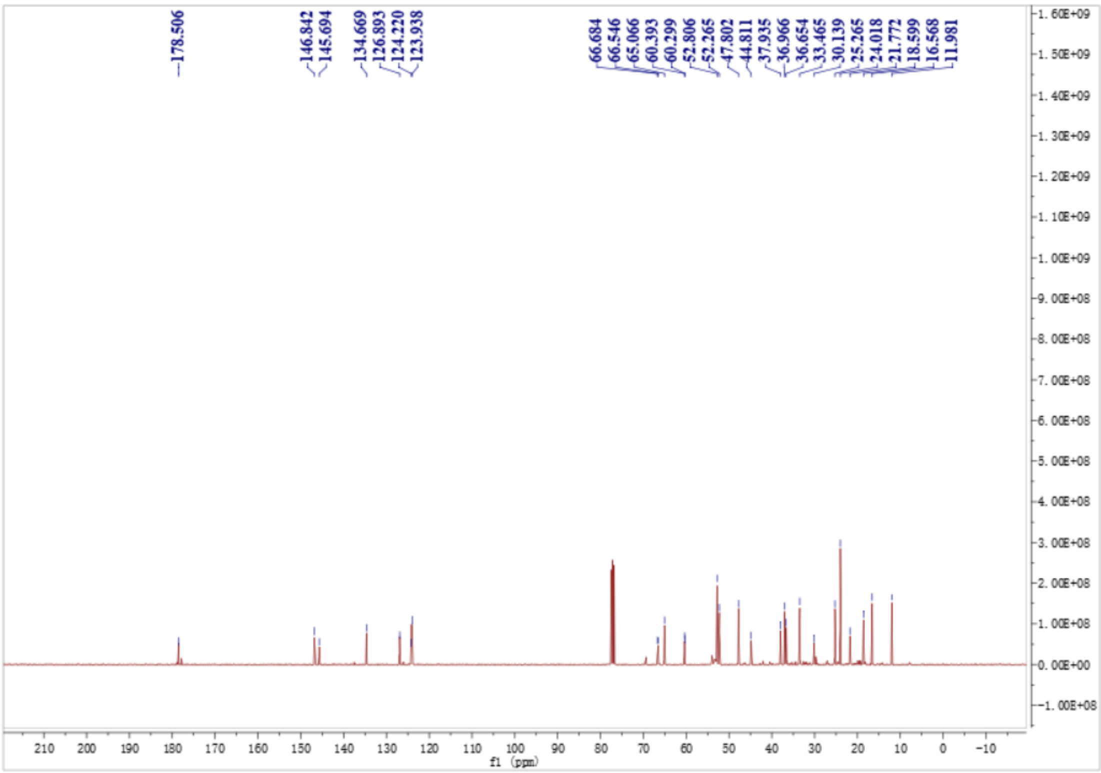


Figure S9. $^{13}C$ NMR Spectrum ($CDCl_3$, 101 MHz) of 2b.

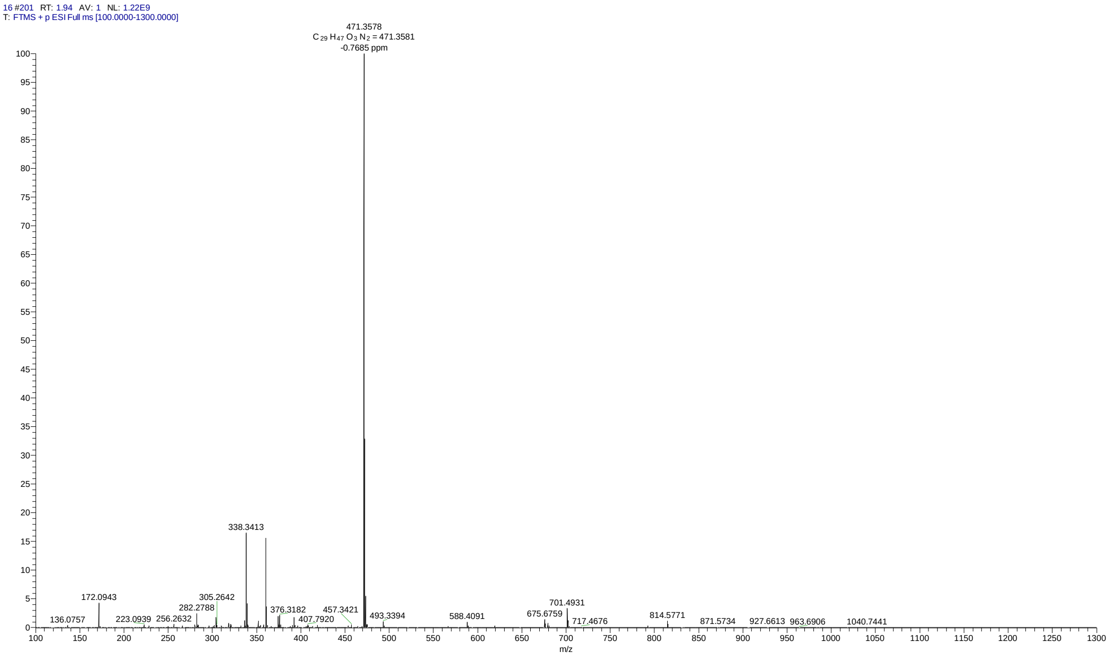


Figure S10. HRMS Spectrum of 2b.

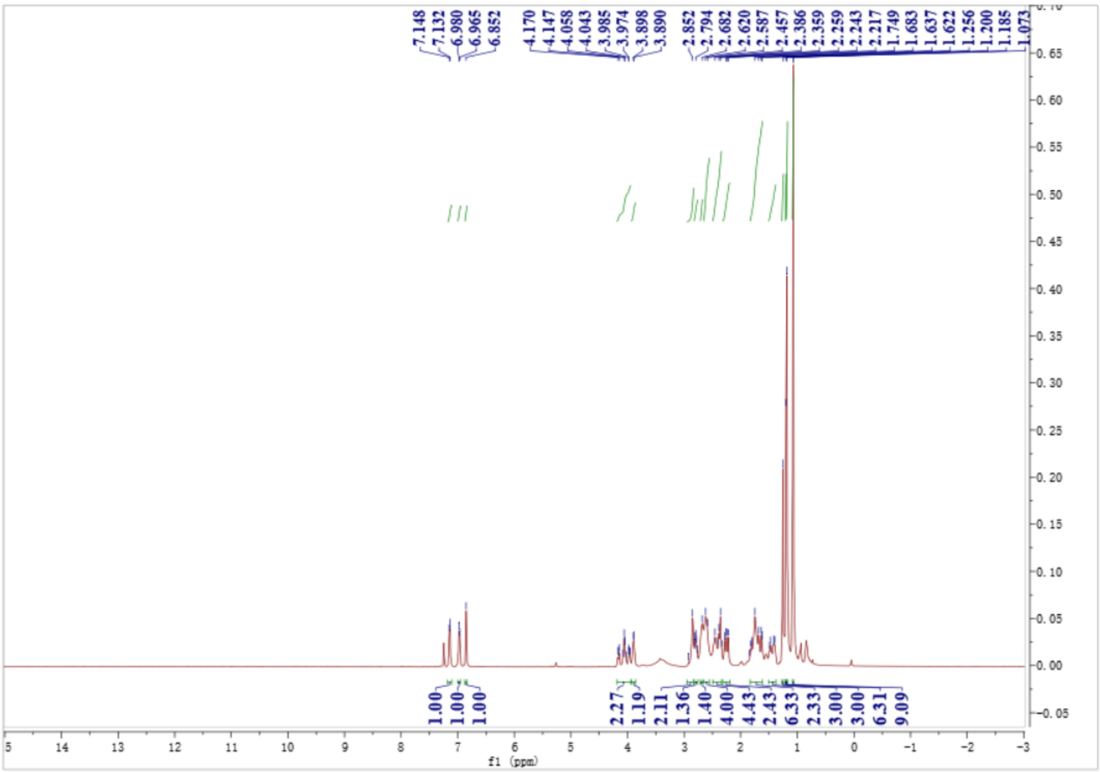


Figure S11. $^{1}$H NMR Spectrum ($CDCl_3$, 500 MHz) of 2c.

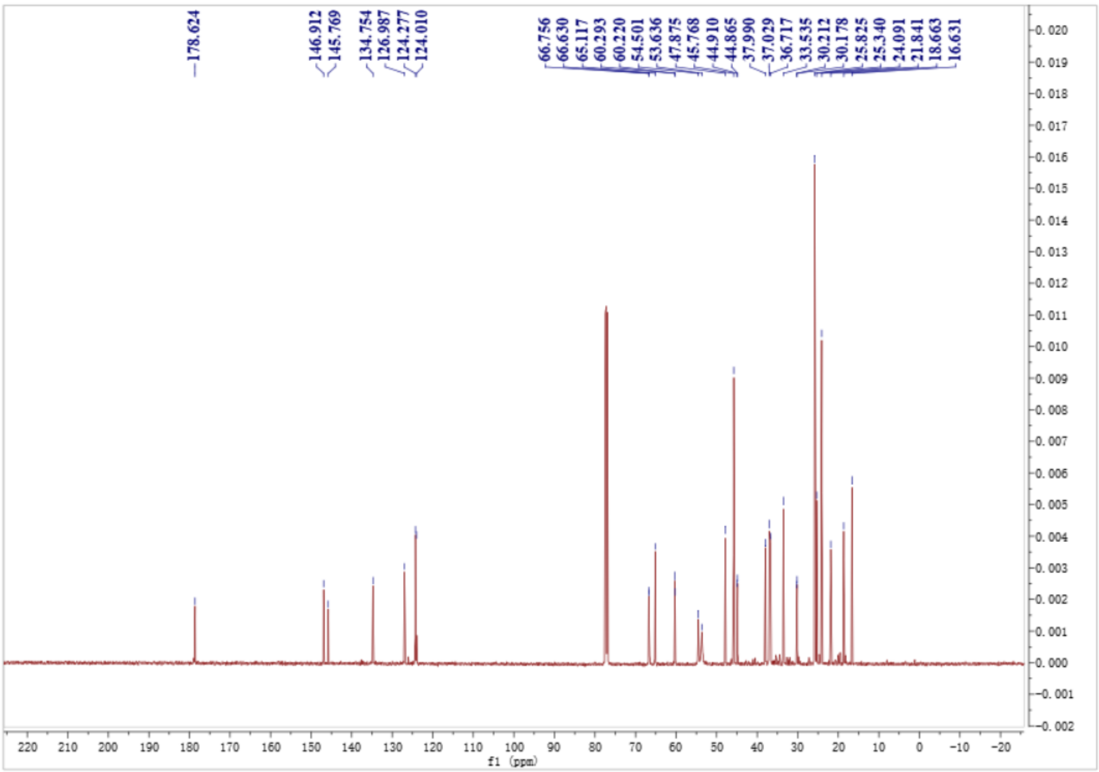


Figure S12. $^{13}$C NMR Spectrum ($CDCl_3$, 126 MHz) of 2c.

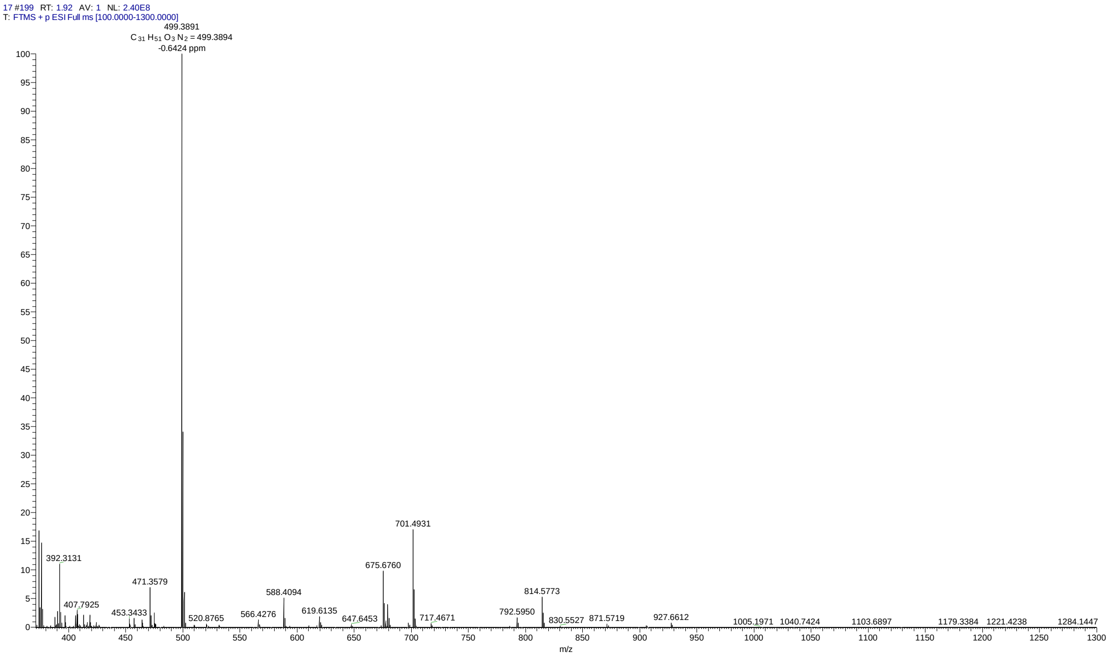


Figure S13. HRMS Spectrum of 2c.

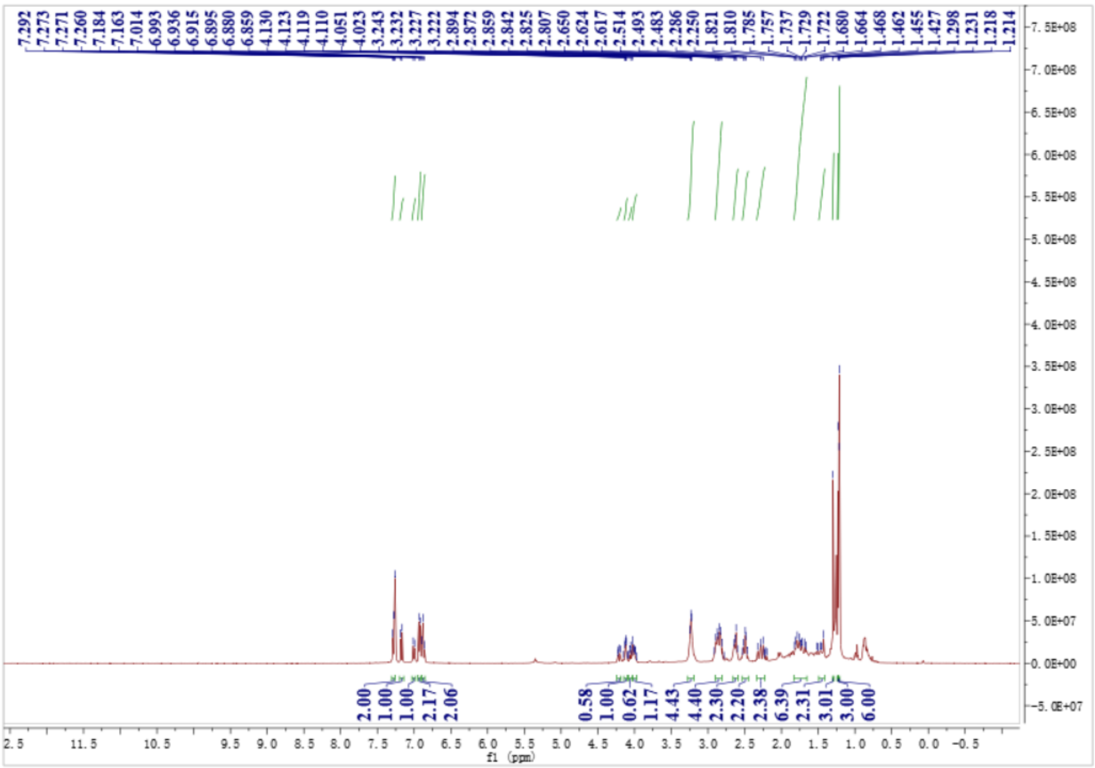


Figure S14. $^{1}$H NMR Spectrum (CDCl3, 400 MHz) of 2d.

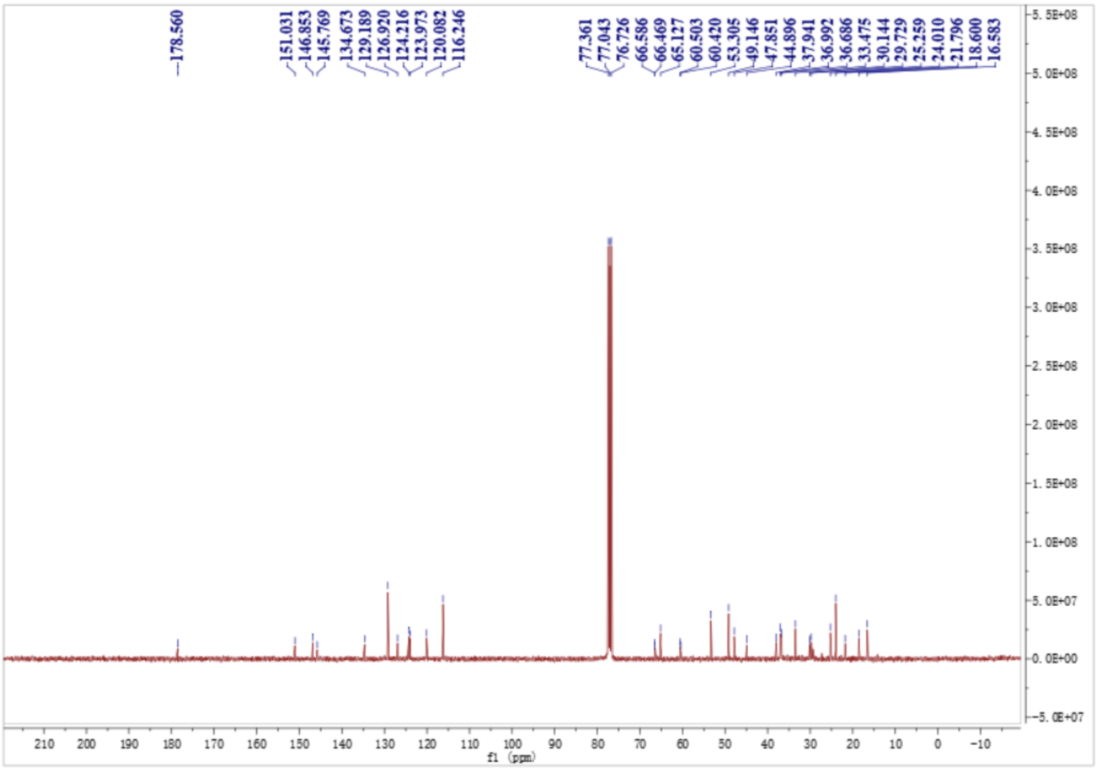


Figure S15. $^{13}$C NMR Spectrum (CDCl3, 101 MHz) of 2d.

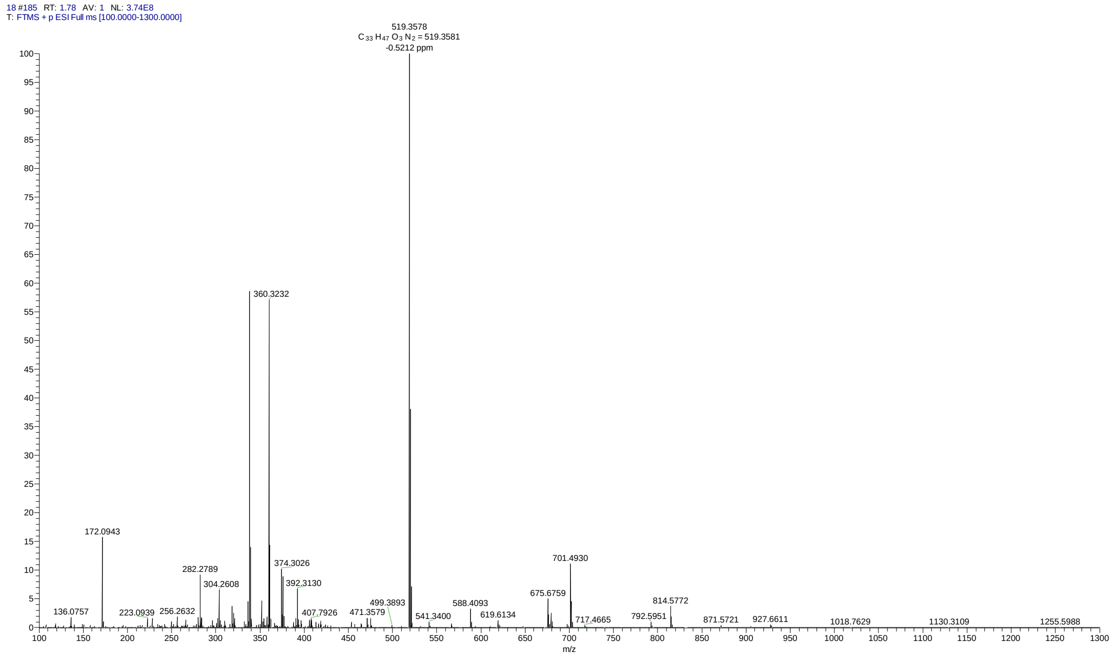


Figure S16. HRMS Spectrum of 2d.

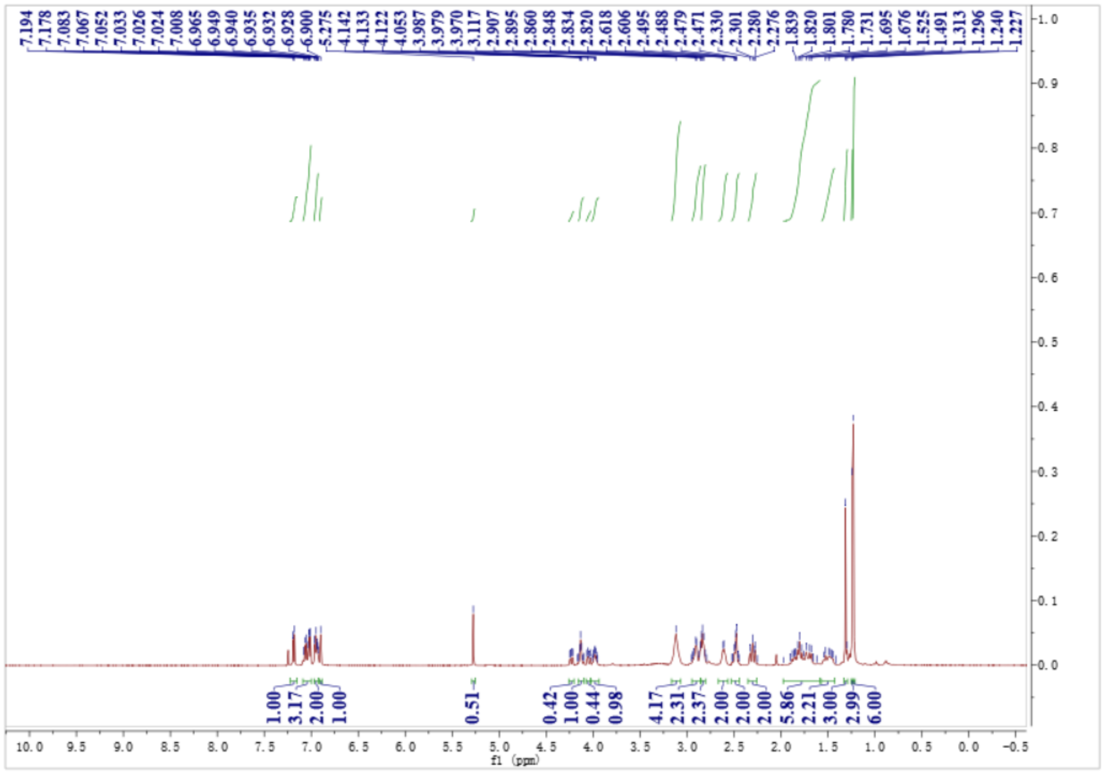


Figure S17. $^{1}$H NMR Spectrum ($CDCl_3$, 500 MHz) of 2e.

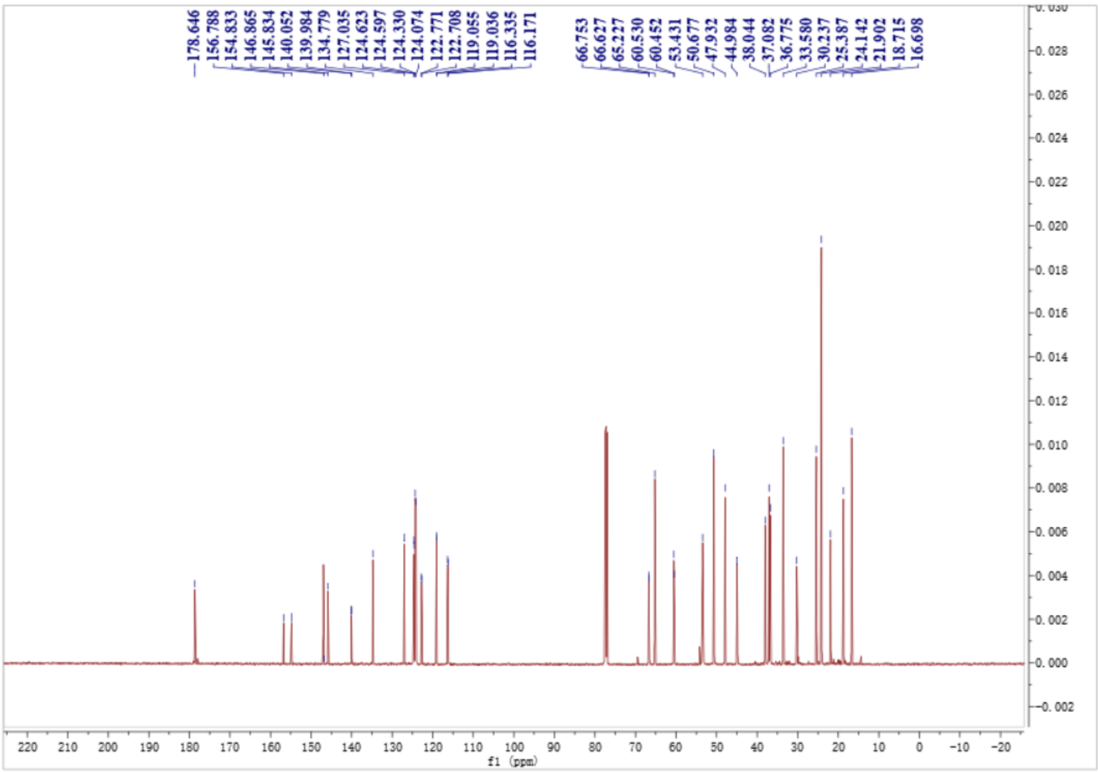


Figure S18. $^{13}$C NMR Spectrum (CDCl₃, 126 MHz) of 2e.

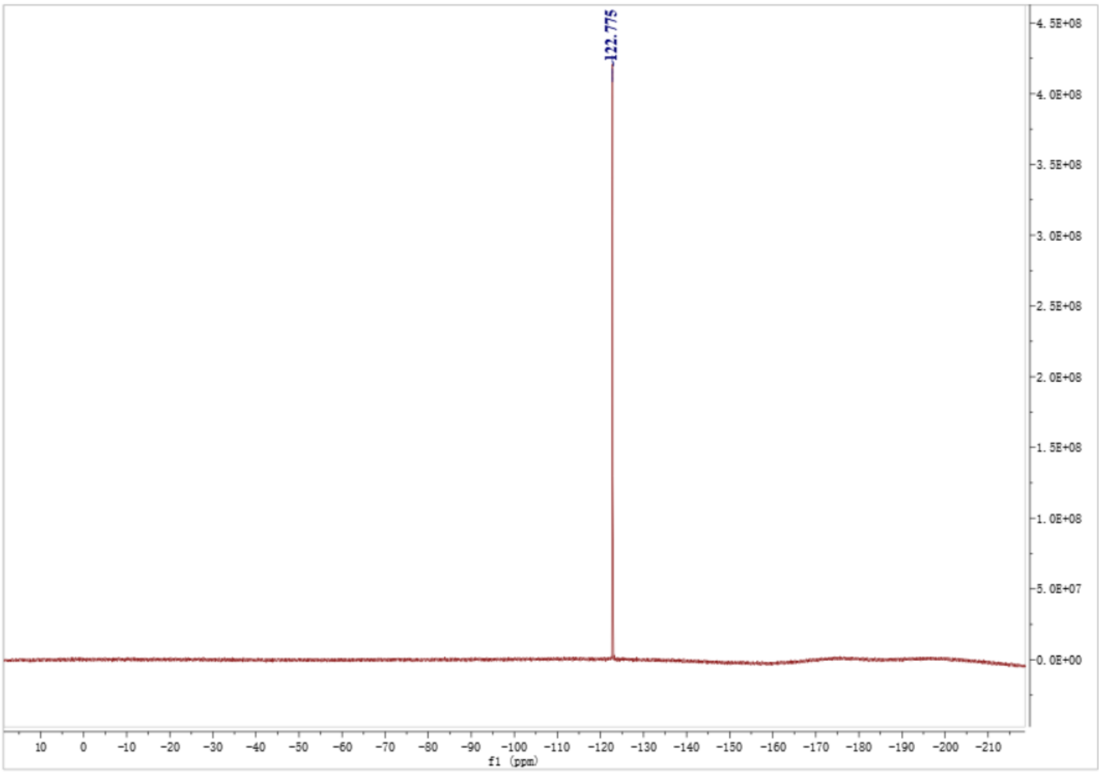


Figure S19. $^{19}$F NMR Spectrum (DMSO-$d_6$, 376 MHz) of 2e.

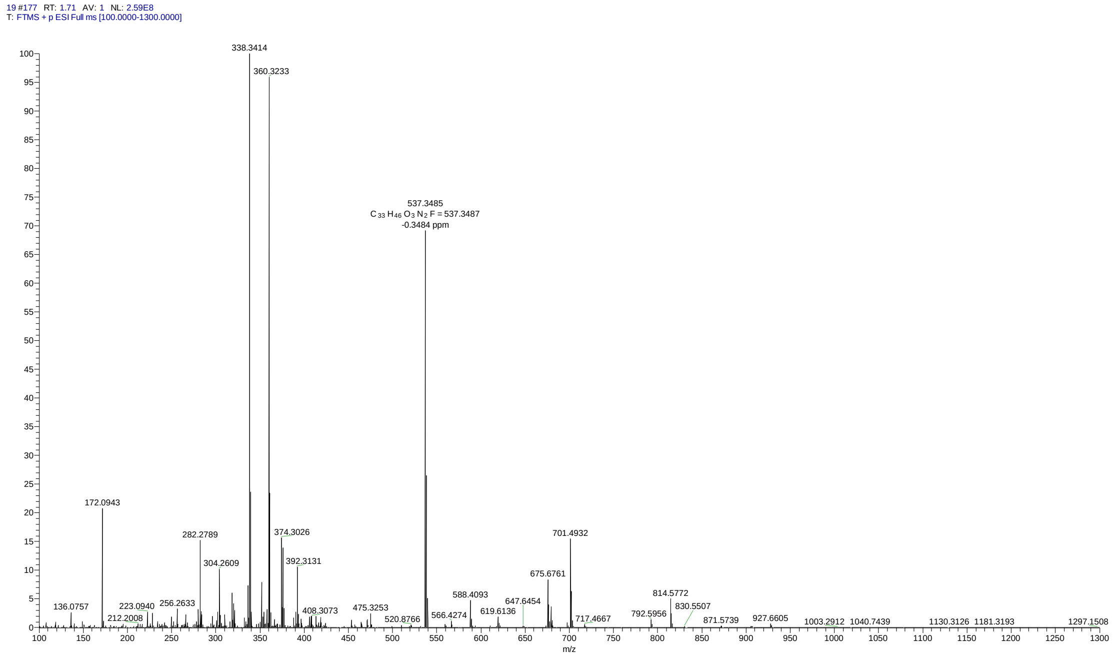


Figure S20. HRMS Spectrum of 2e.

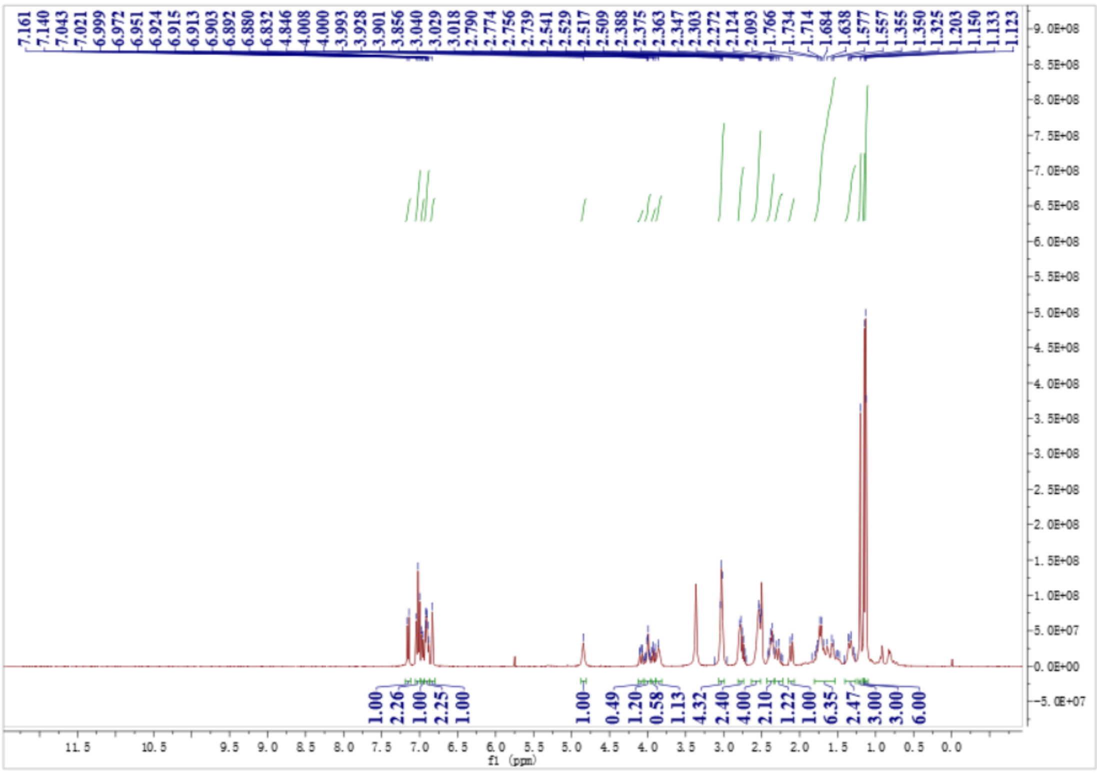


Figure S21. $^{1}$H NMR Spectrum (DMSO-$d_6$, 400 MHz) of 2f.

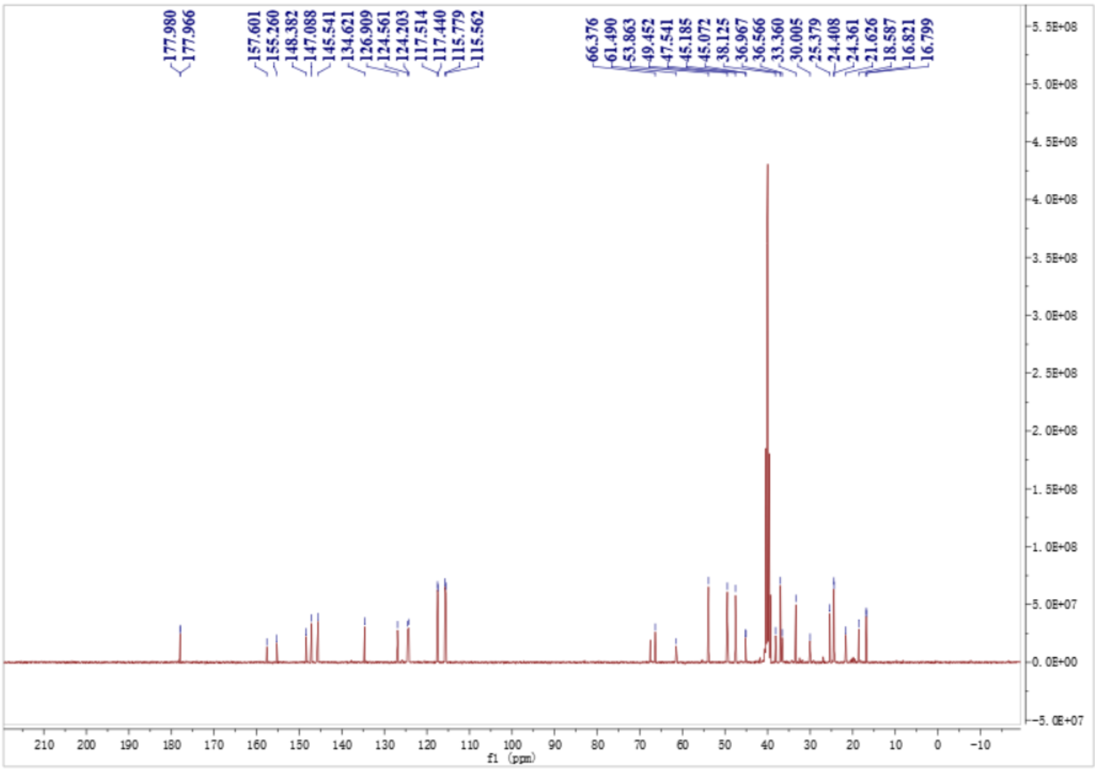


Figure S22. $^{13}$C NMR Spectrum (DMSO-$d_6$, 101 MHz) of 2f.

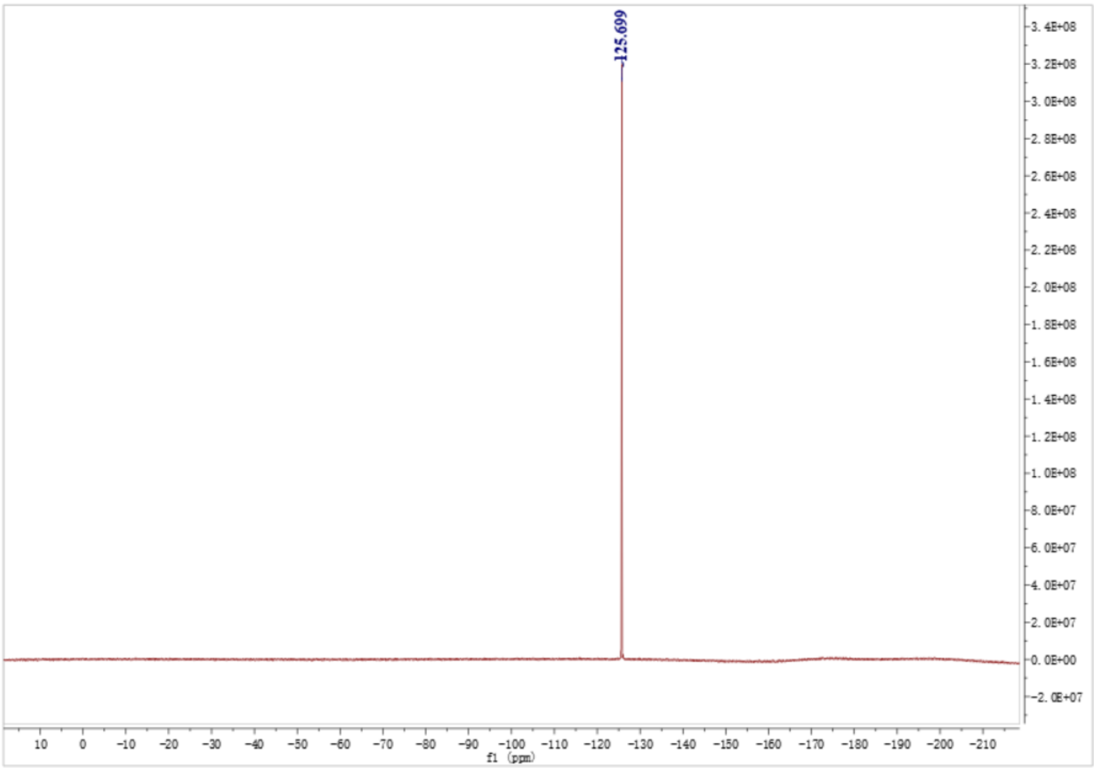


Figure S23. $^{19}$F NMR Spectrum (DMSO-$d_6$, 376 MHz) of 2f.

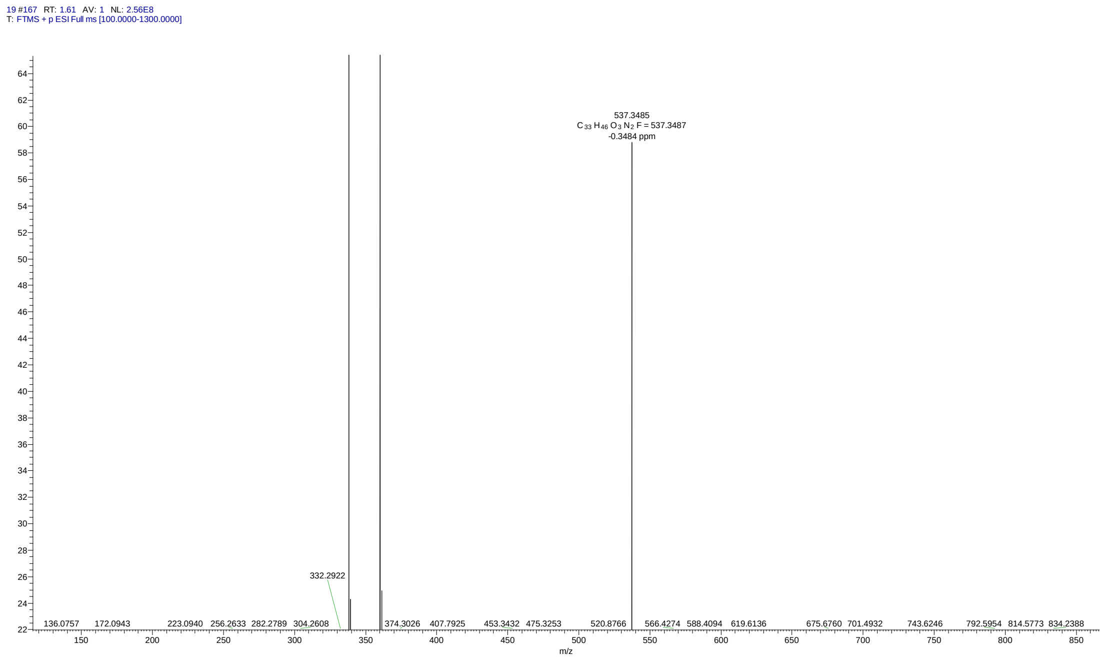


Figure S24. HRMS Spectrum of 2f.

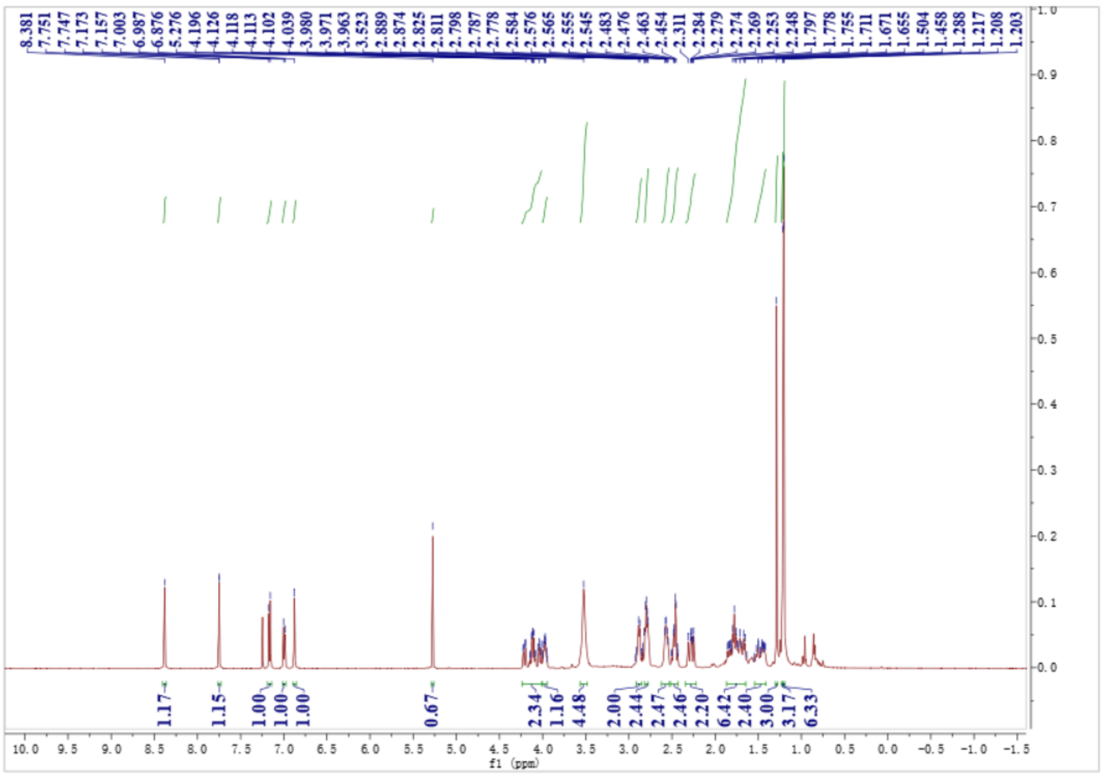


Figure S25. $^1$H NMR Spectrum ($CDCl_3$, 500 MHz) of 2g.

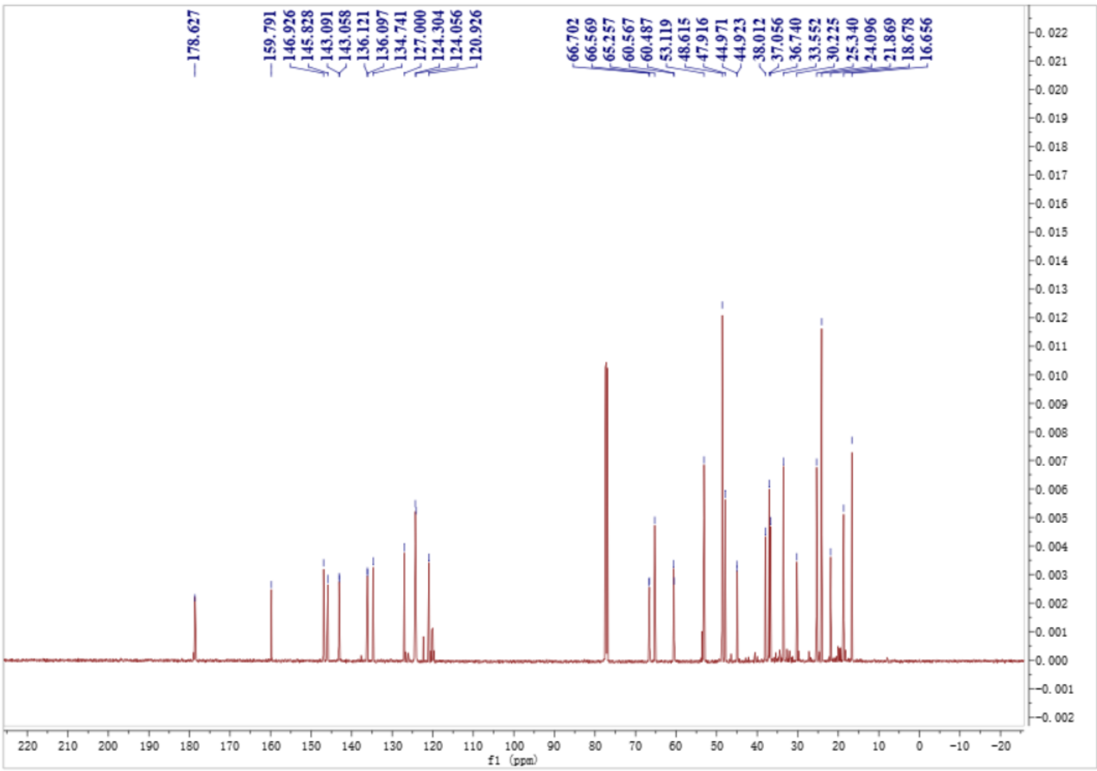


Figure S26. $^{13}$C NMR Spectrum ($CDCl_3$, 126 MHz) of 2g.

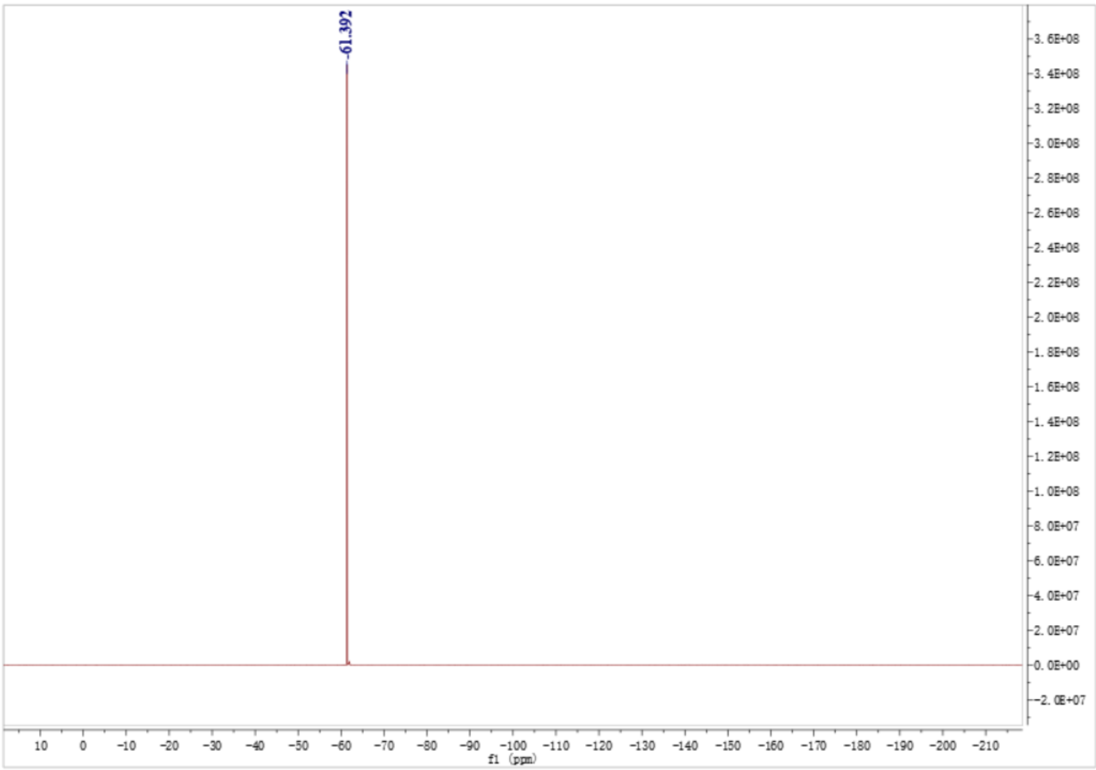


Figure S27. $^{19}$F NMR Spectrum ($CDCl_3$, 376 MHz) of 2g.

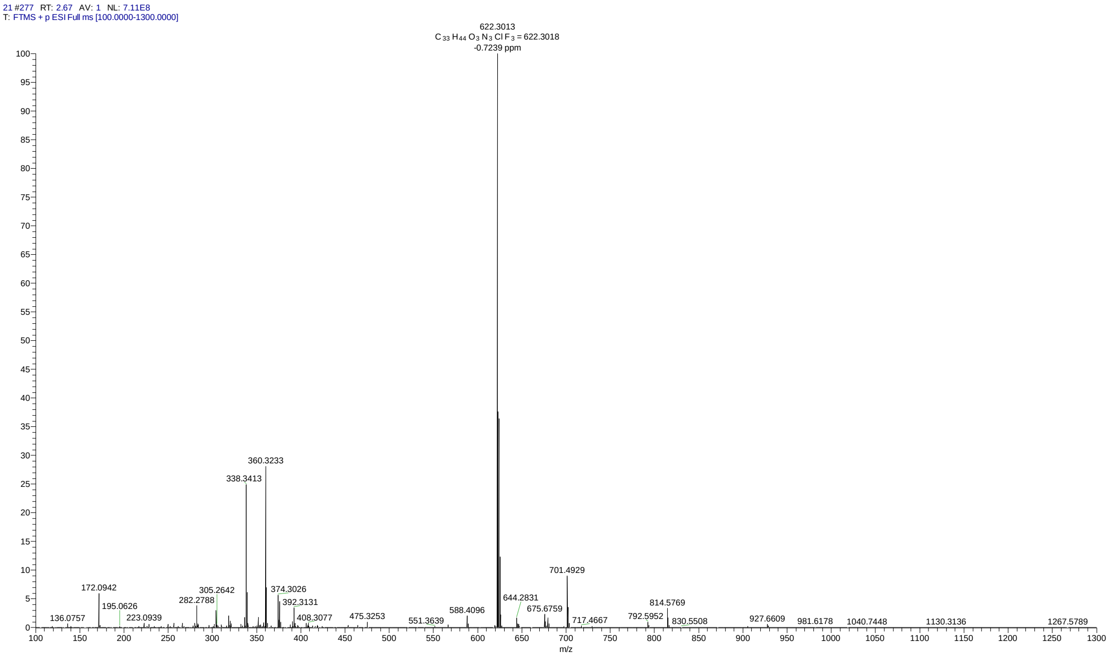


Figure S28. HRMS Spectrum of 2g.

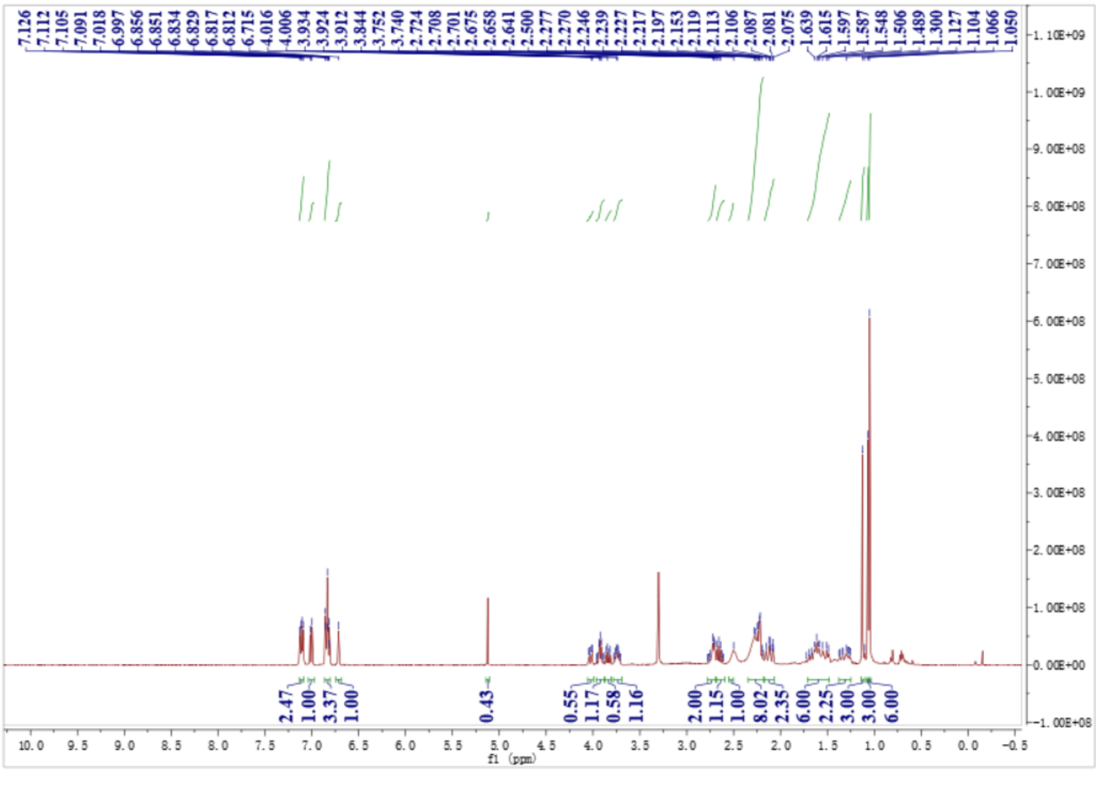


Figure S29. $^{1}$H NMR Spectrum ($CDCl_3$, 400 MHz) of 2h.

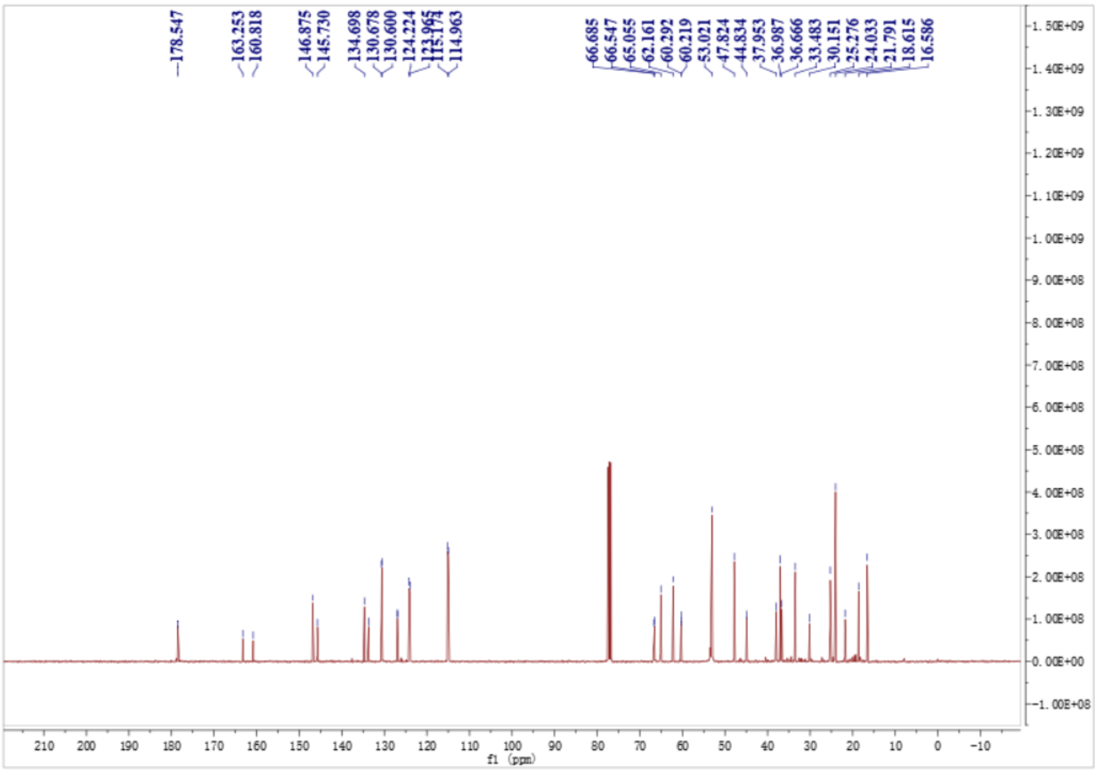


Figure S30. $^{13}C$ NMR Spectrum ($CDCl_3$, 101 MHz) of 2h.

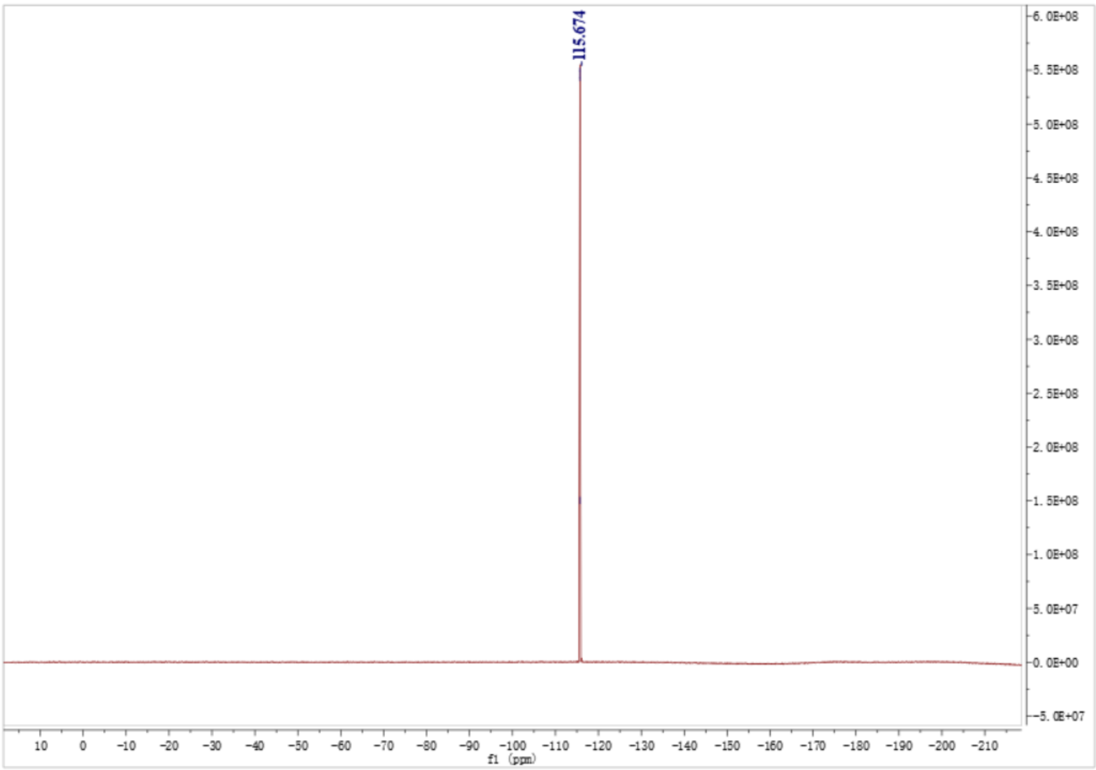


Figure S31. $^{19}F$ NMR Spectrum ($CDCl_3$, 376 MHz) of 2h.

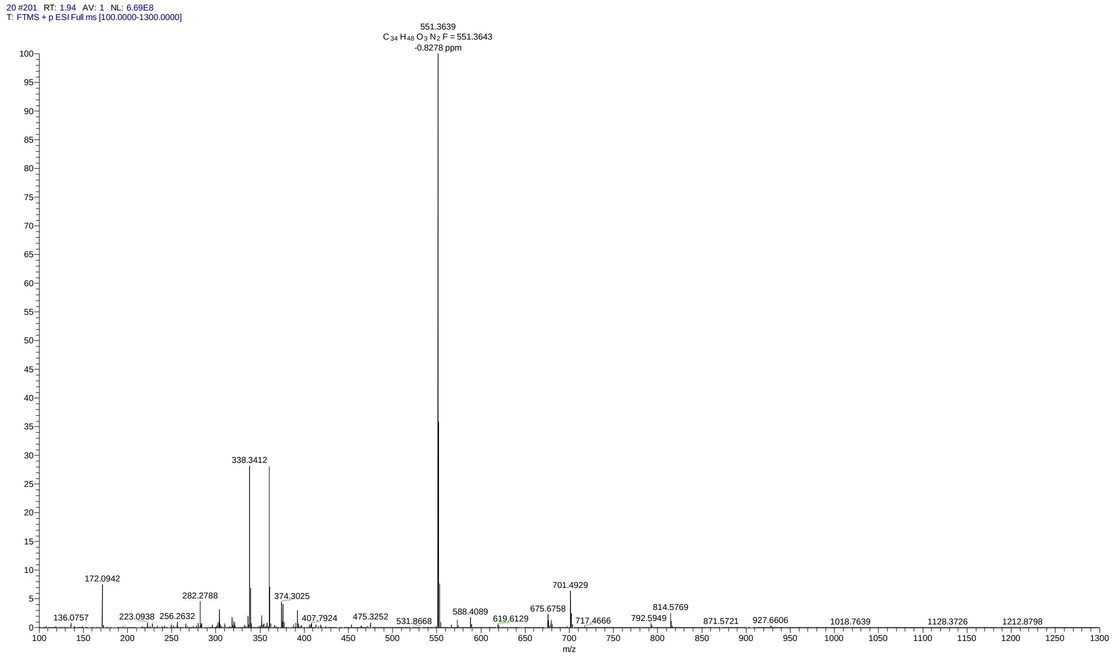


Figure S32. HRMS Spectrum of 2h.

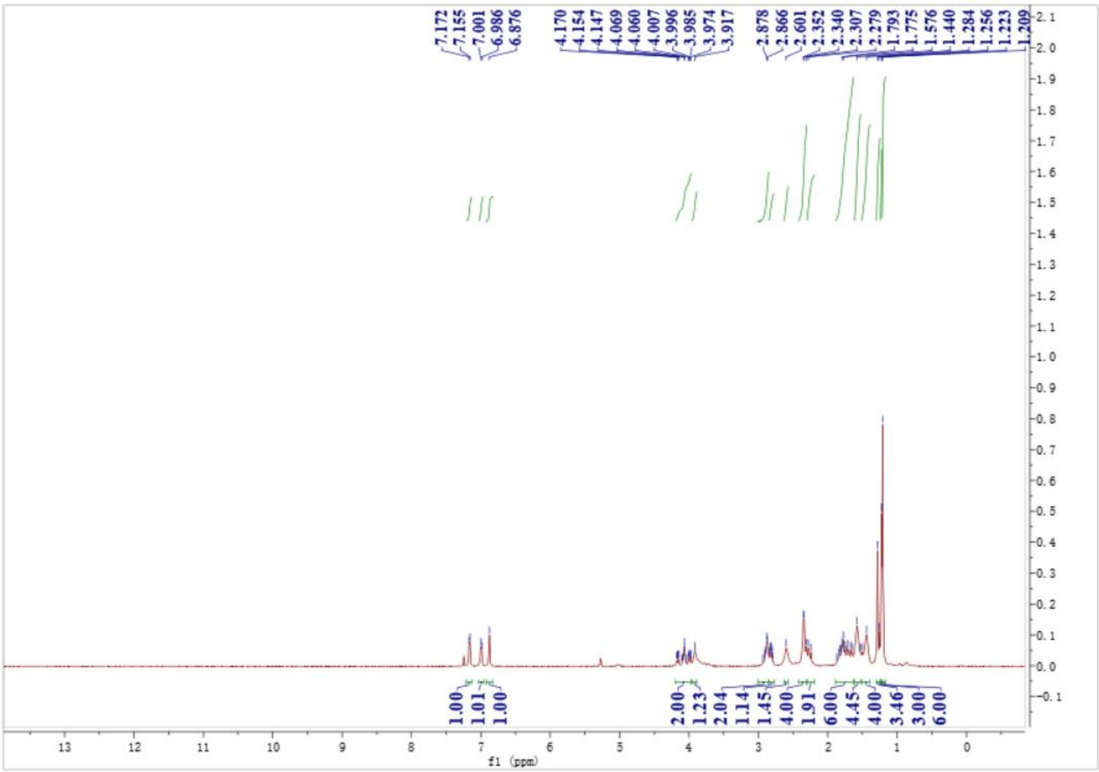


Figure S33. $^{1}$H NMR Spectrum ($CDCl_3$, 500 MHz) of 2i.

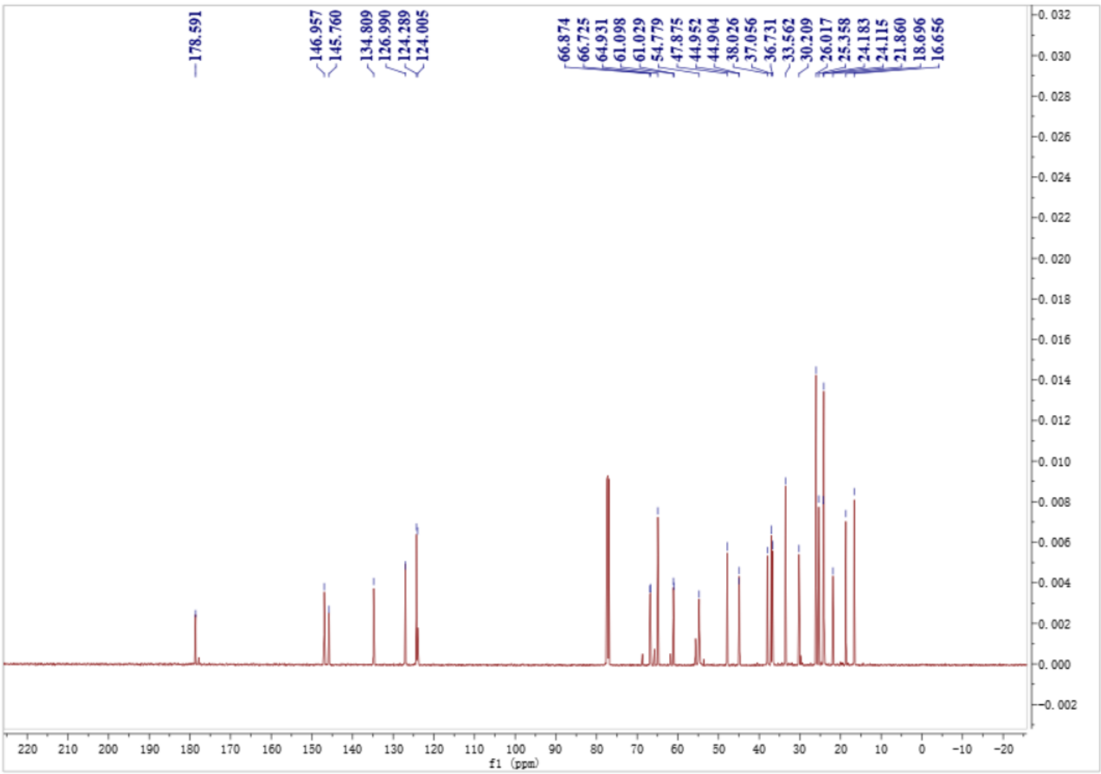

Figure S34. $^{13}$C NMR Spectrum ($CDCl_3$, 126 MHz) of 2i.

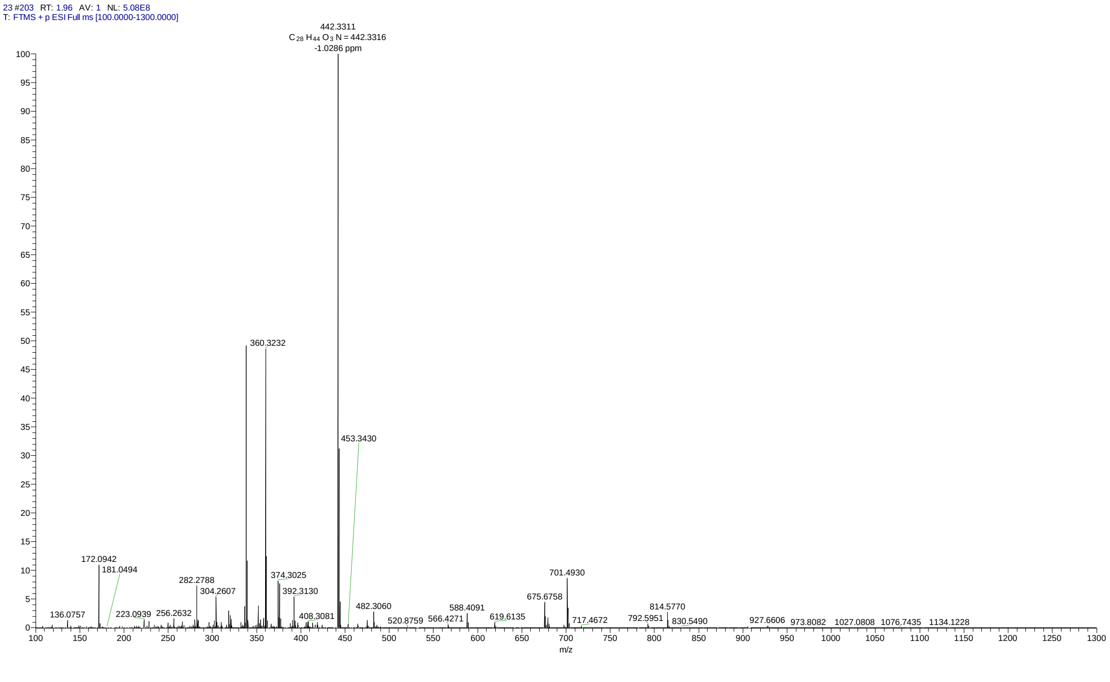

Figure S35. HRMS Spectrum of 2i.

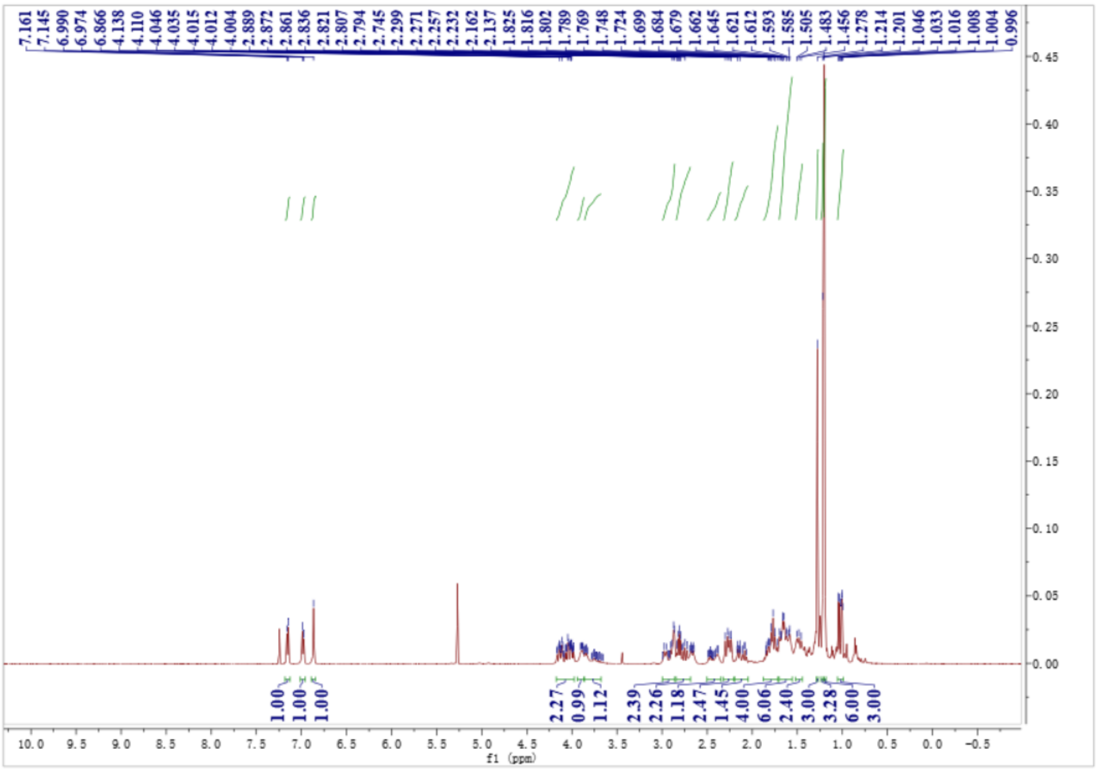


Figure S36. $^{1}$H NMR Spectrum (CDCl₃, 500 MHz) of 2j.

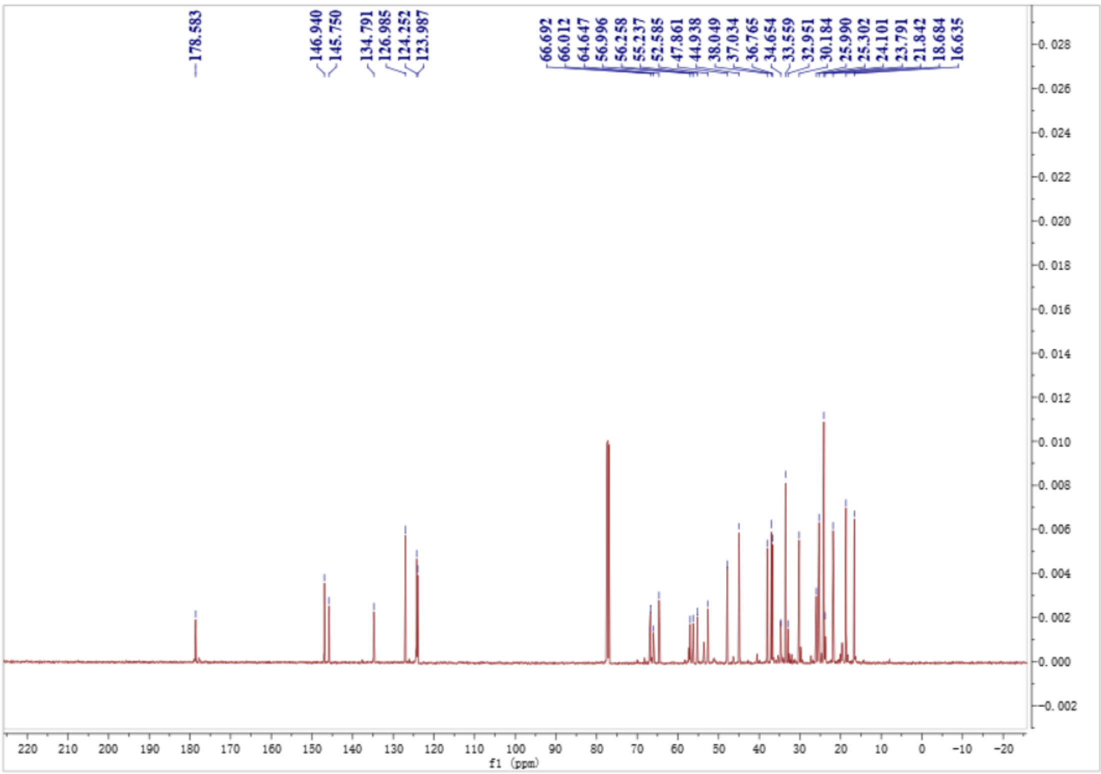


Figure S37. $^{13}$C NMR Spectrum (CDCl₃, 126 MHz) of 2j.

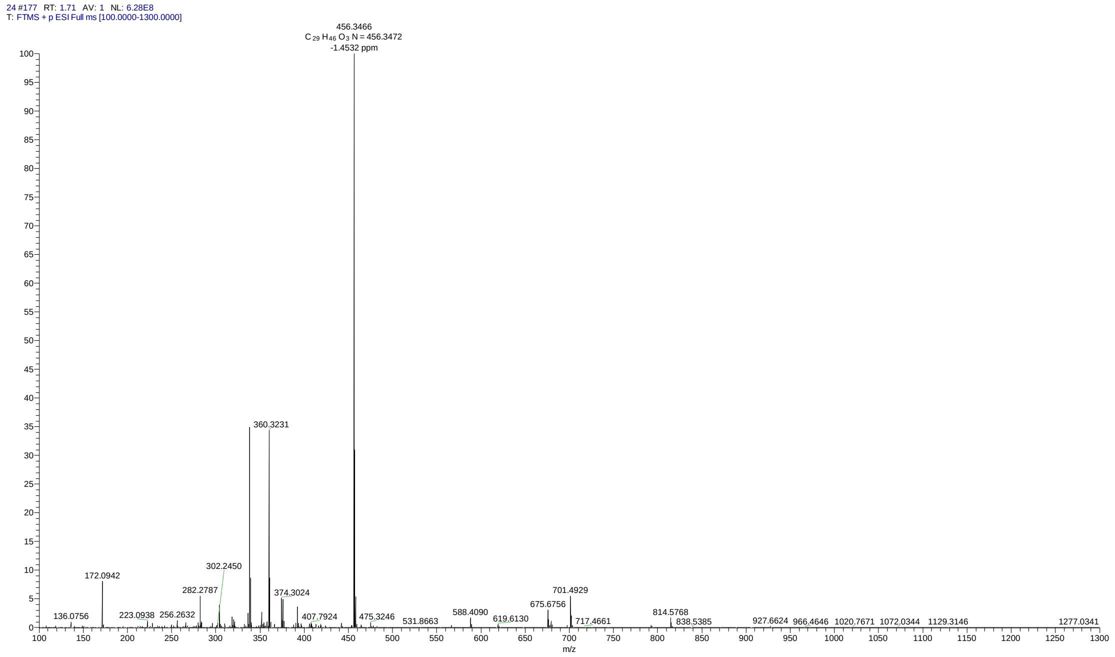


Figure S38. HRMS Spectrum of 2j.

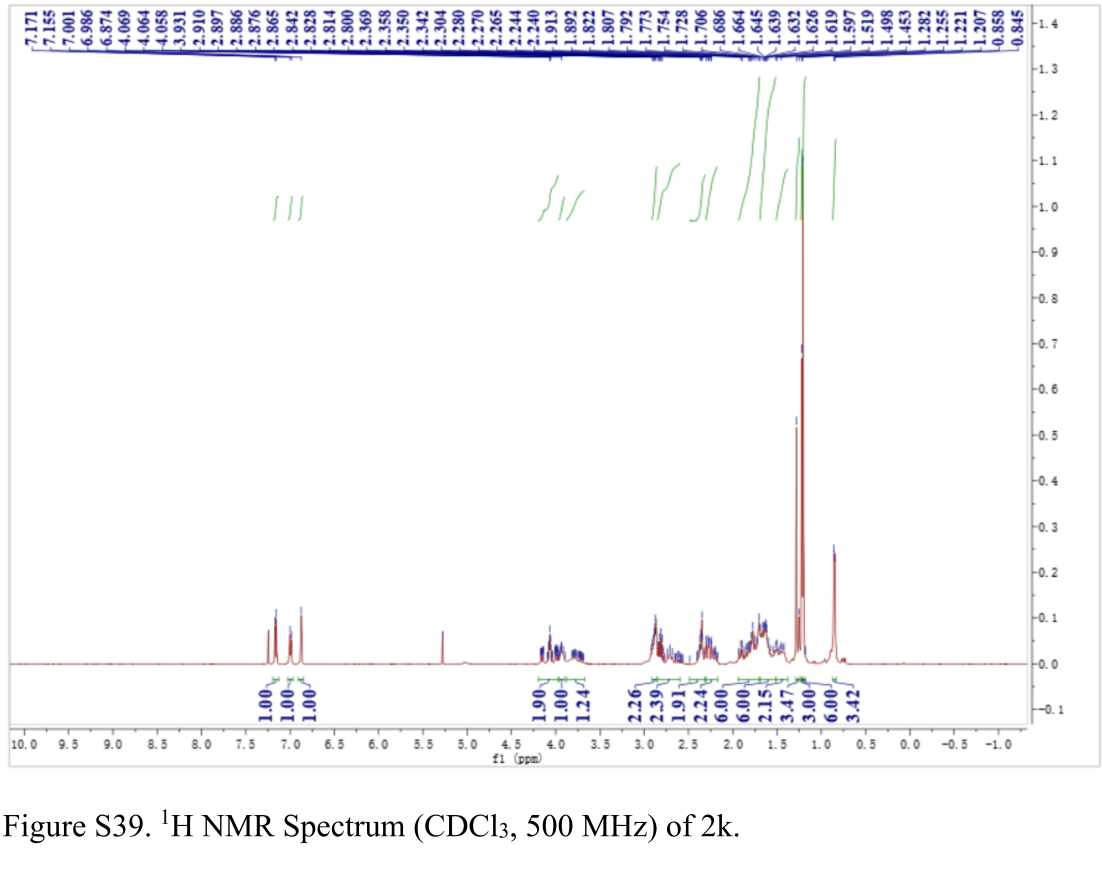


Figure S39. $^{1}$H NMR Spectrum ($CDCl_3$, 500 MHz) of 2k.

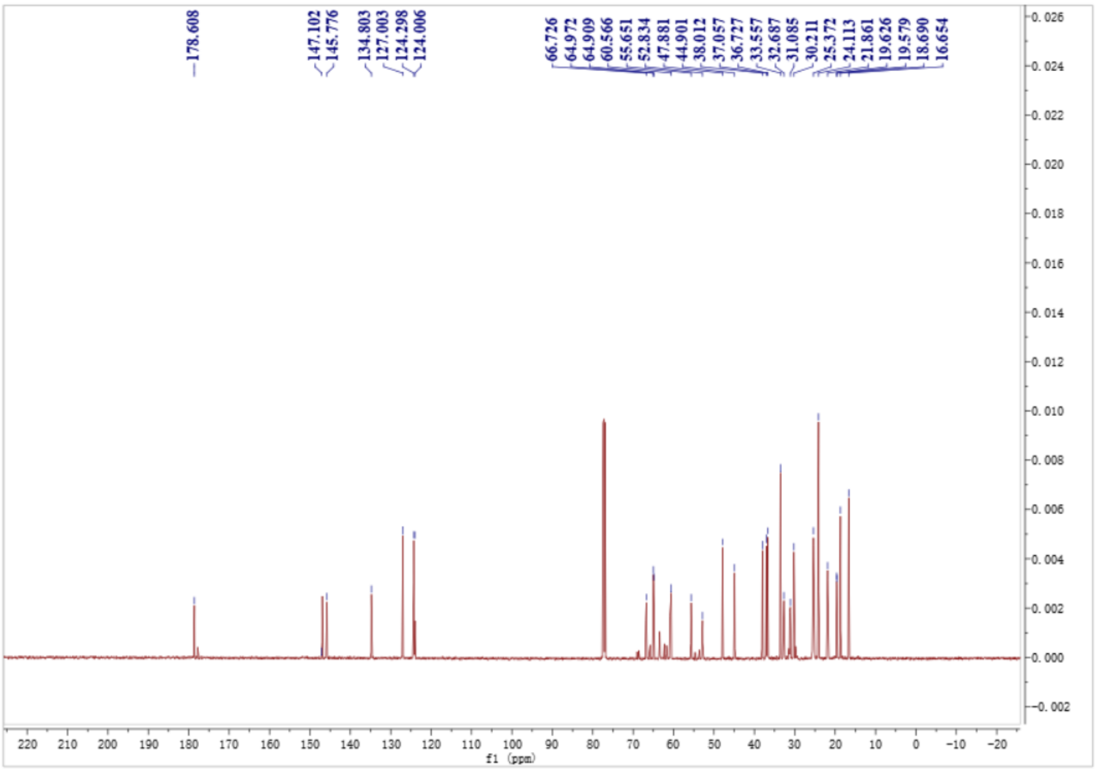


Figure S40. $^{13}$C NMR Spectrum ($CDCl_3$, 126 MHz) of 2k.

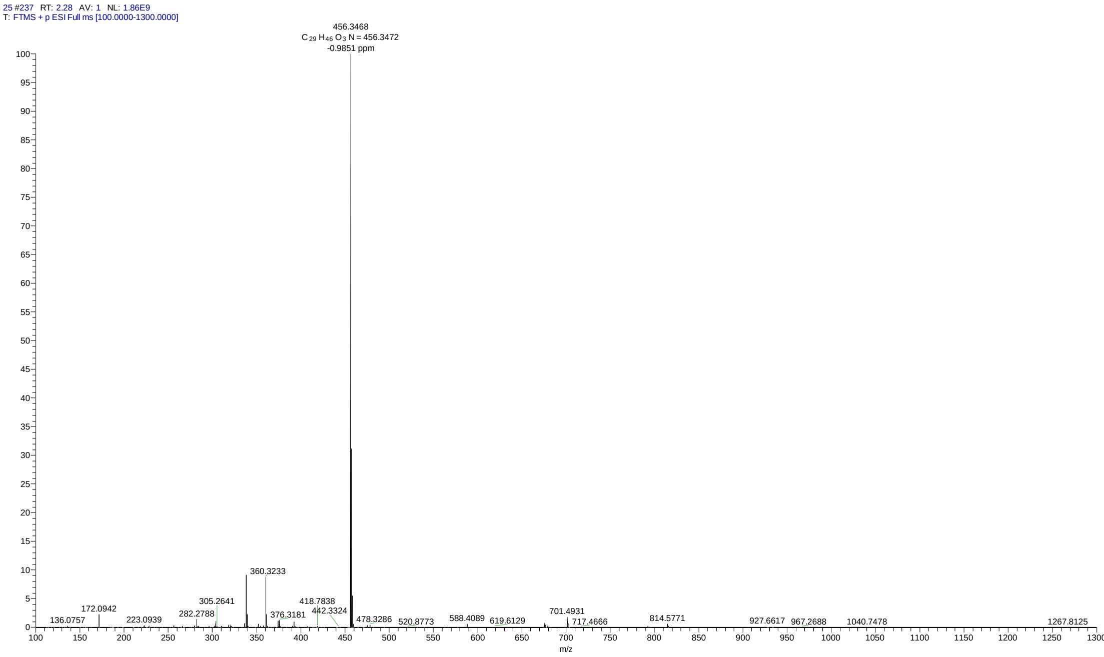


Figure S41. HRMS Spectrum of 2k.

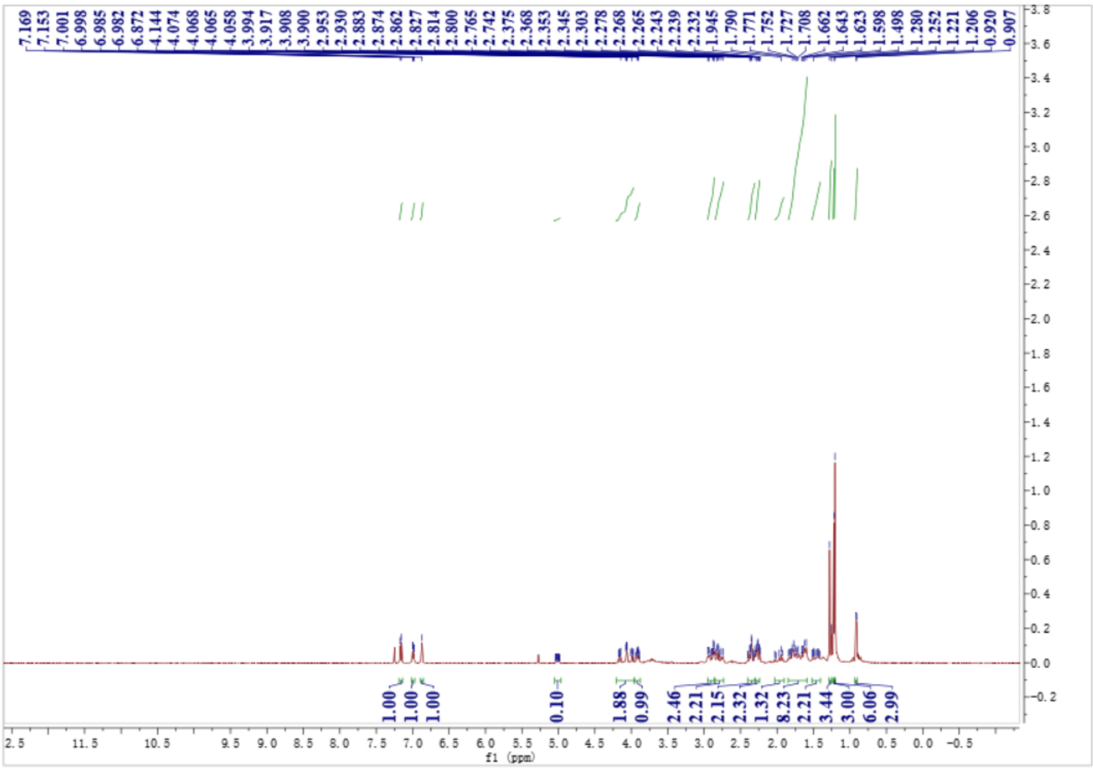


Figure S42. $^{1}$H NMR Spectrum ($CDCl_3$, 500 MHz) of 2l.

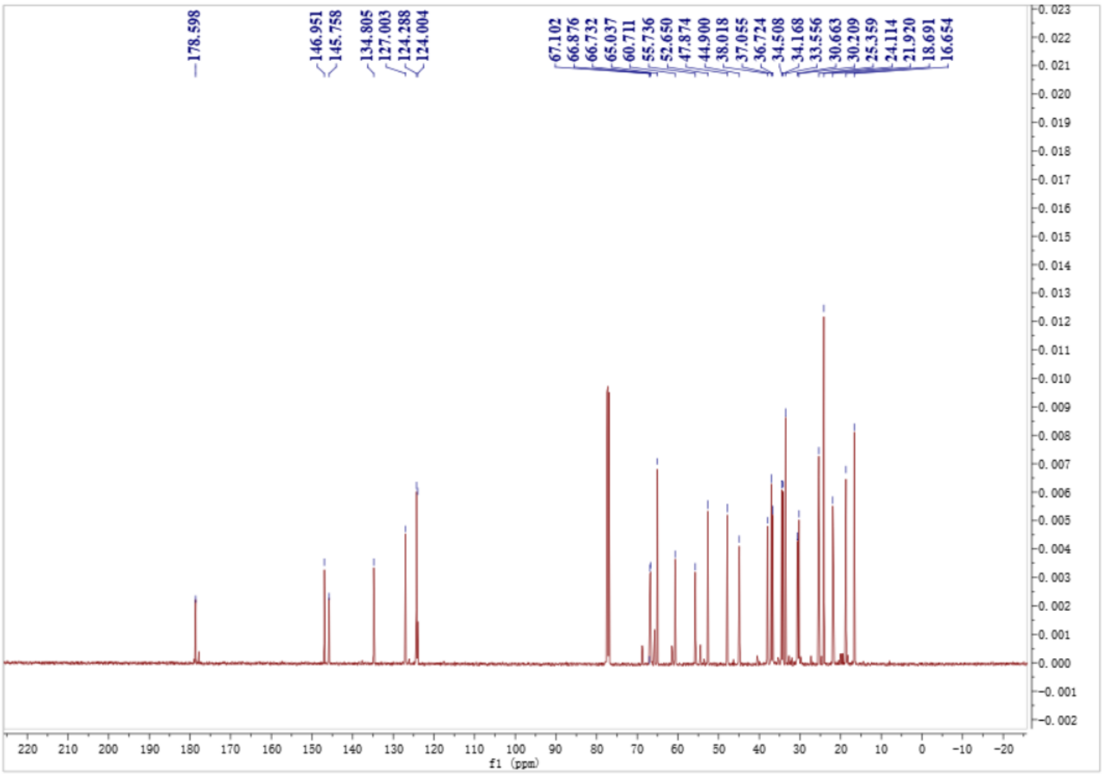


Figure S43. $^{13}$C NMR Spectrum ($CDCl_3$, 126 MHz) of 2l.

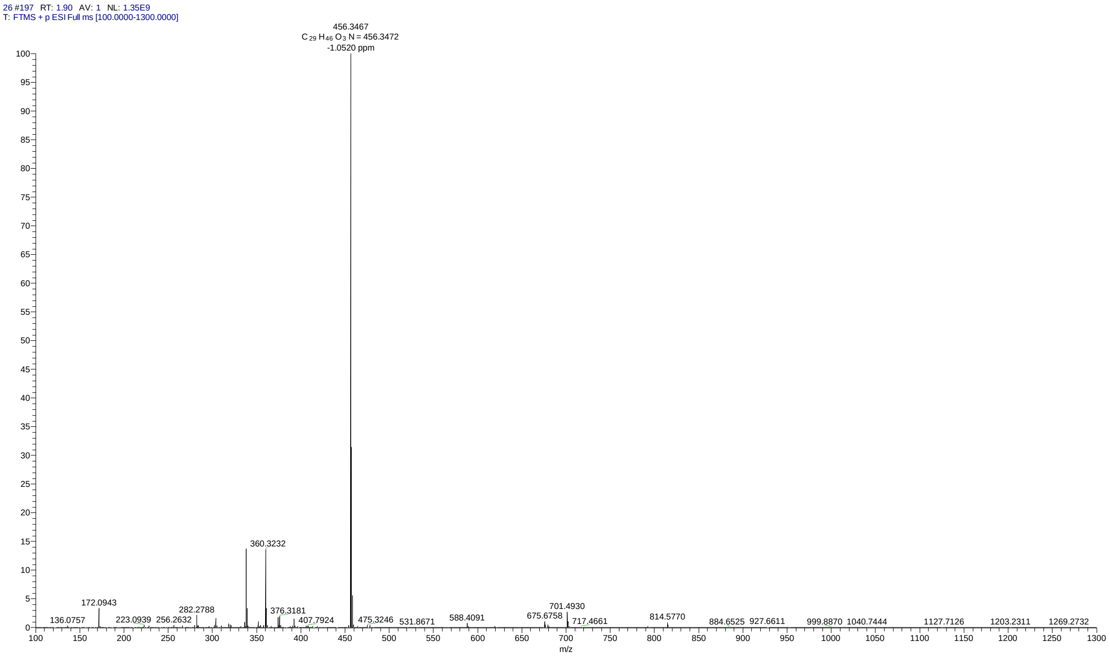


Figure S44. HRMS Spectrum of 2l.

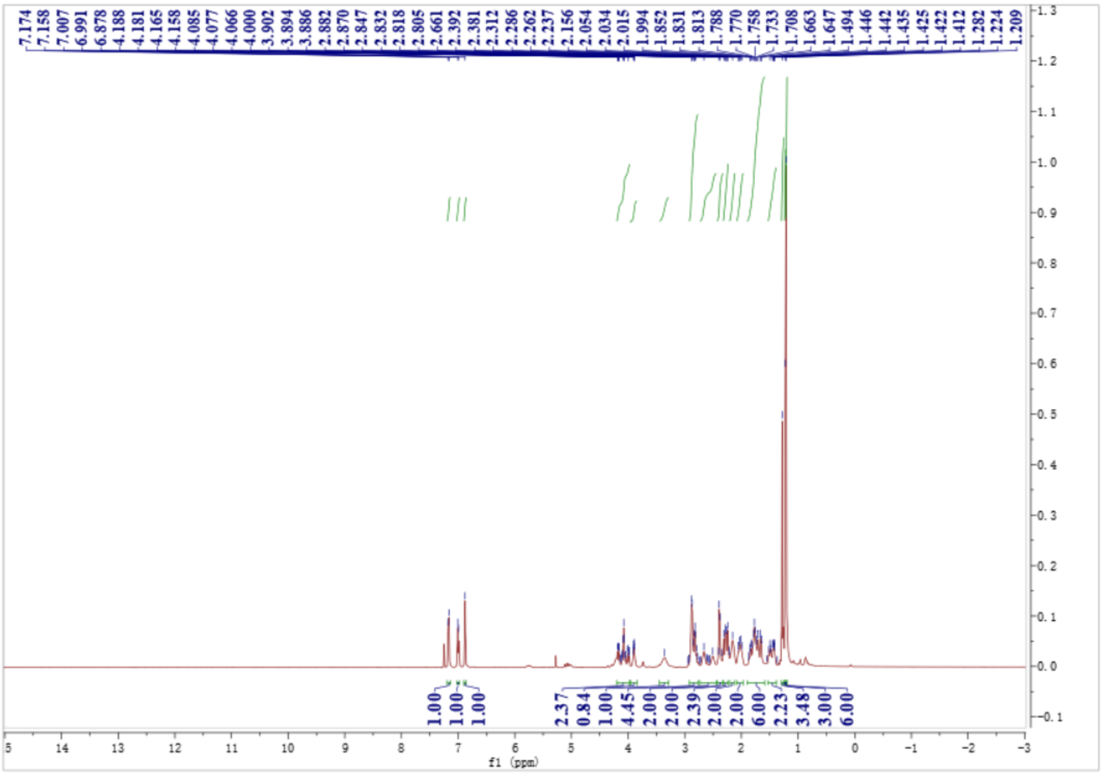


Figure S45. $^{1}$H NMR Spectrum ($CDCl_3$, 500 MHz) of 2m.

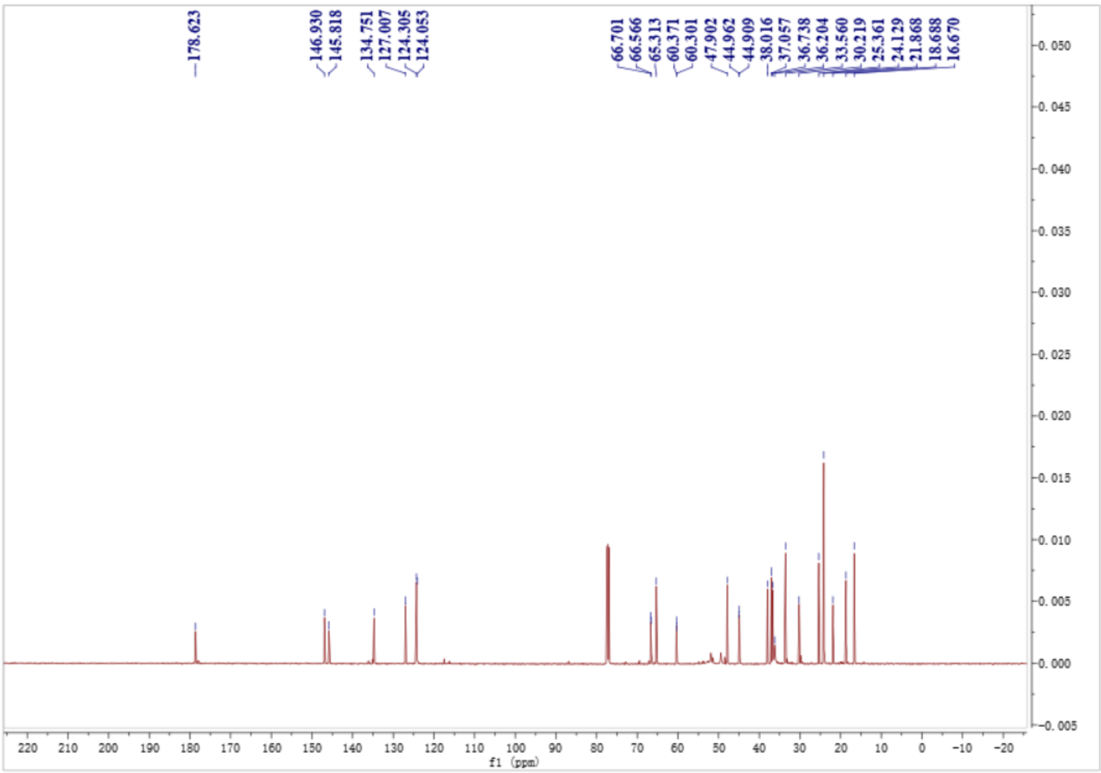


Figure S46. $^{13}$C NMR Spectrum ($CDCl_3$, 126 MHz) of 2m.

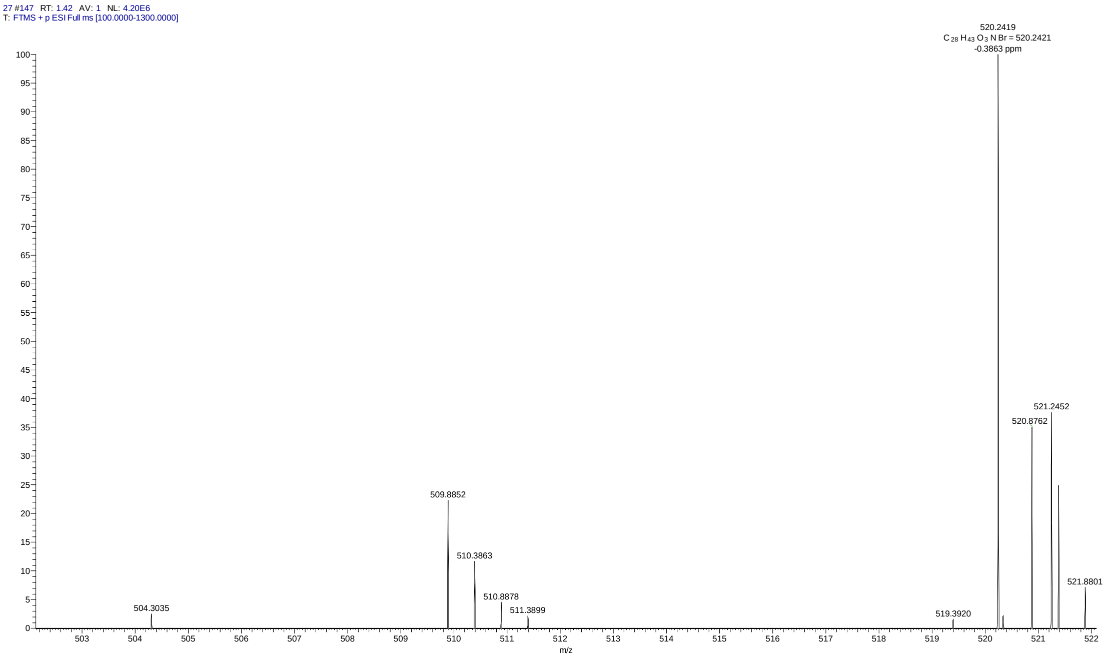


Figure S47. HRMS Spectrum of 2m.

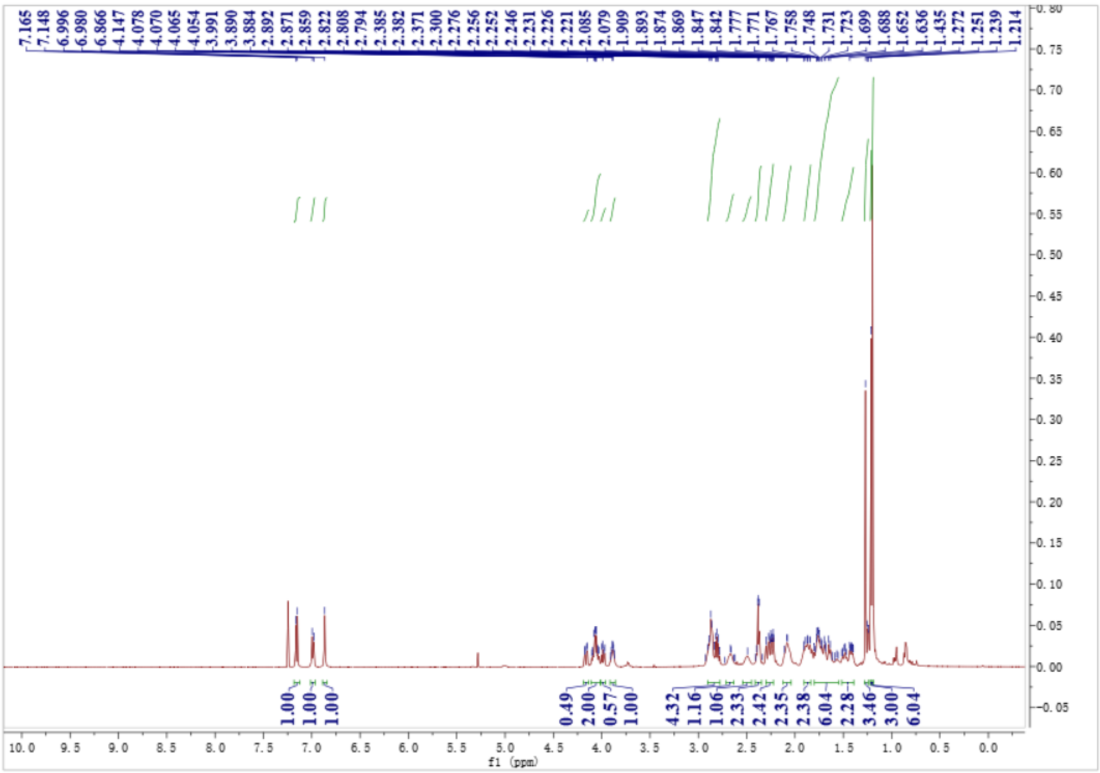

Figure S48. $^{1}$H NMR Spectrum ($CDCl_3$, 500 MHz) of 2n.

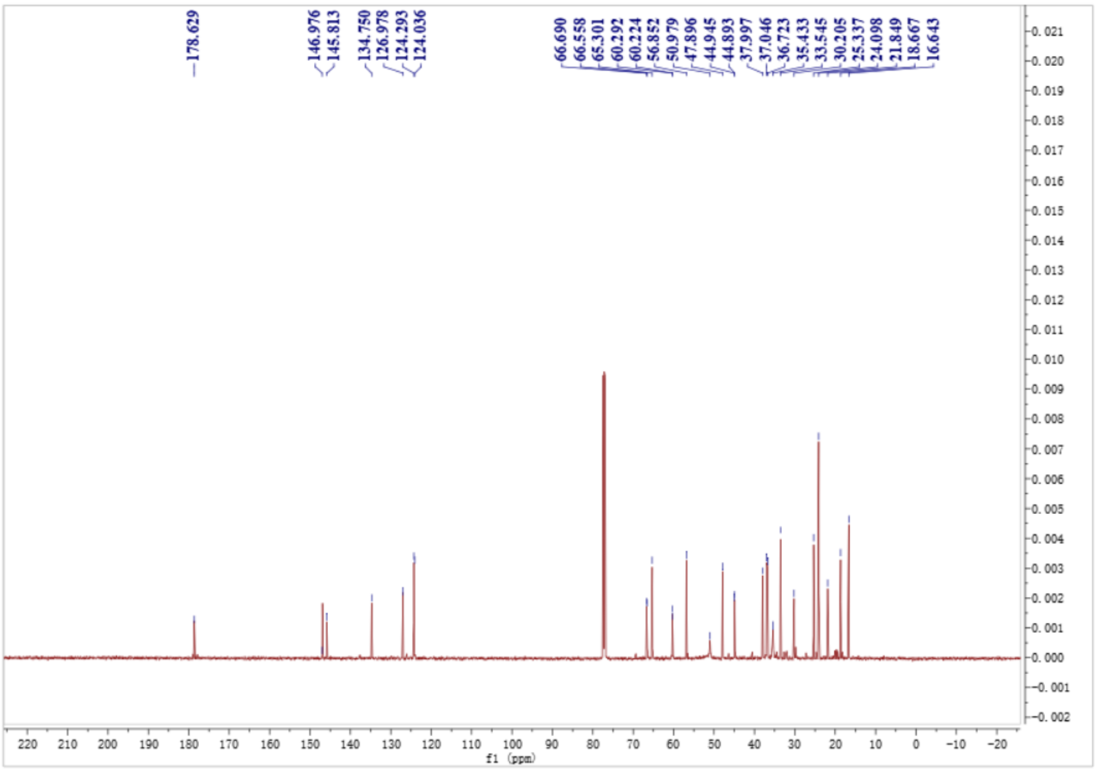

Figure S49. $^{13}$C NMR Spectrum ($CDCl_3$, 126 MHz) of 2n.

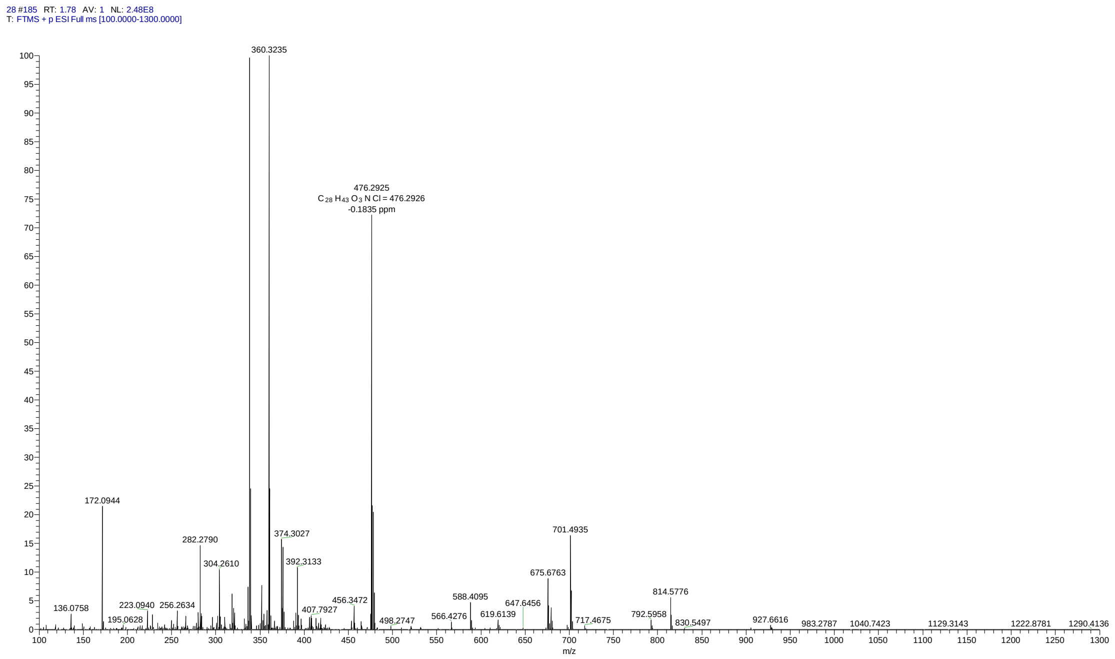

Figure S50. HRMS Spectrum of 2n.

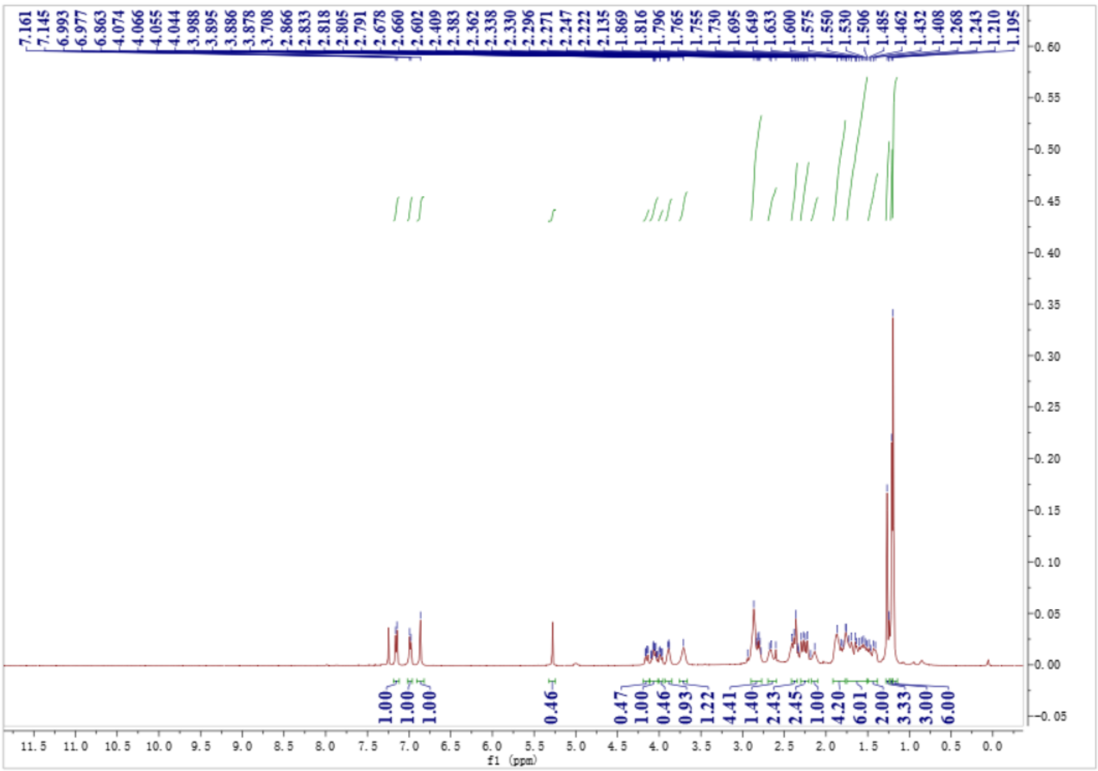

Figure S51. $^{1}H$ NMR Spectrum ($CDCl_3$, 500 MHz) of 2o.

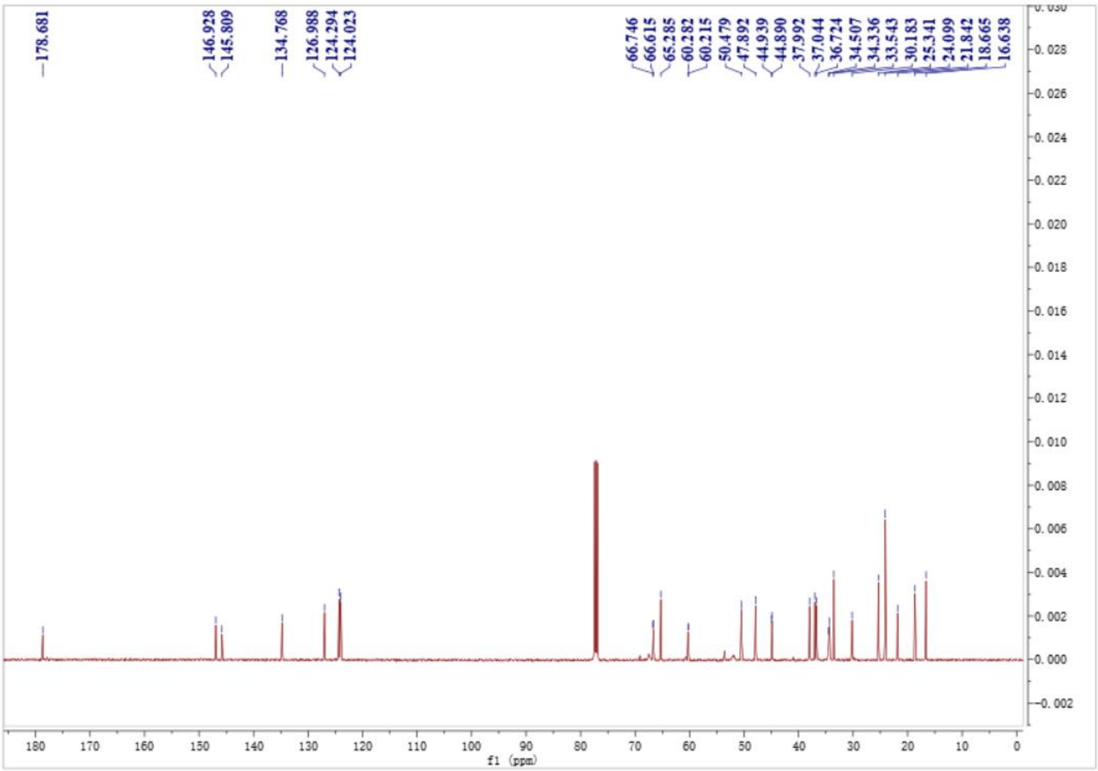


Figure S52. $^{13}$C NMR Spectrum ($CDCl_3$, 126 MHz) of 2o.

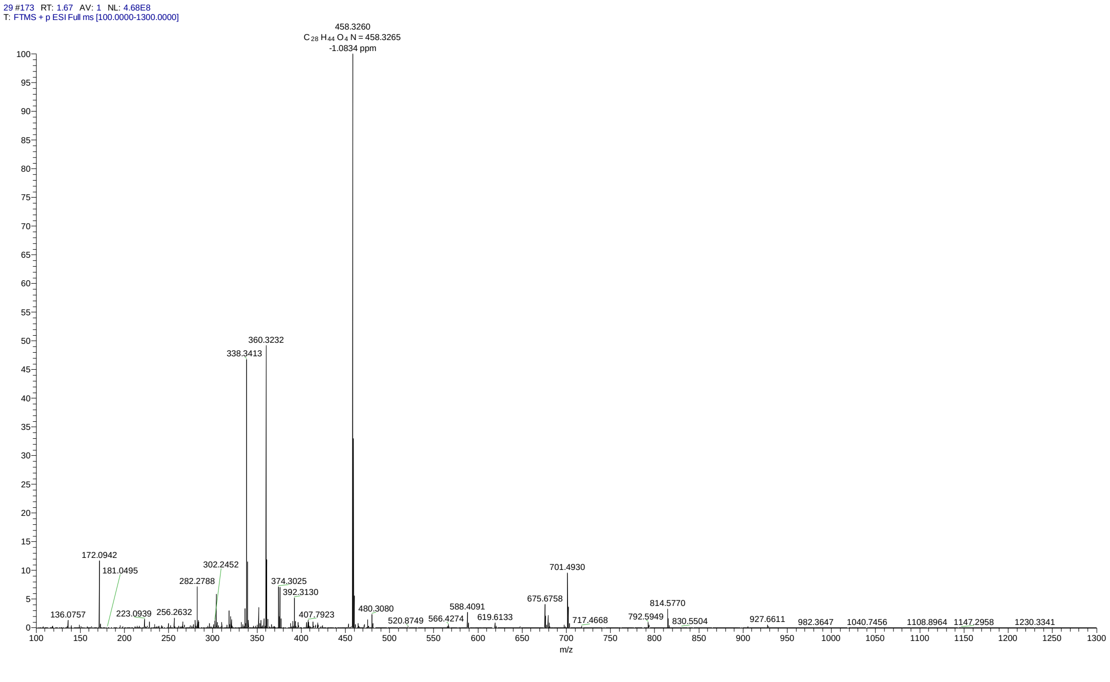


Figure S53. HRMS Spectrum of 2o.

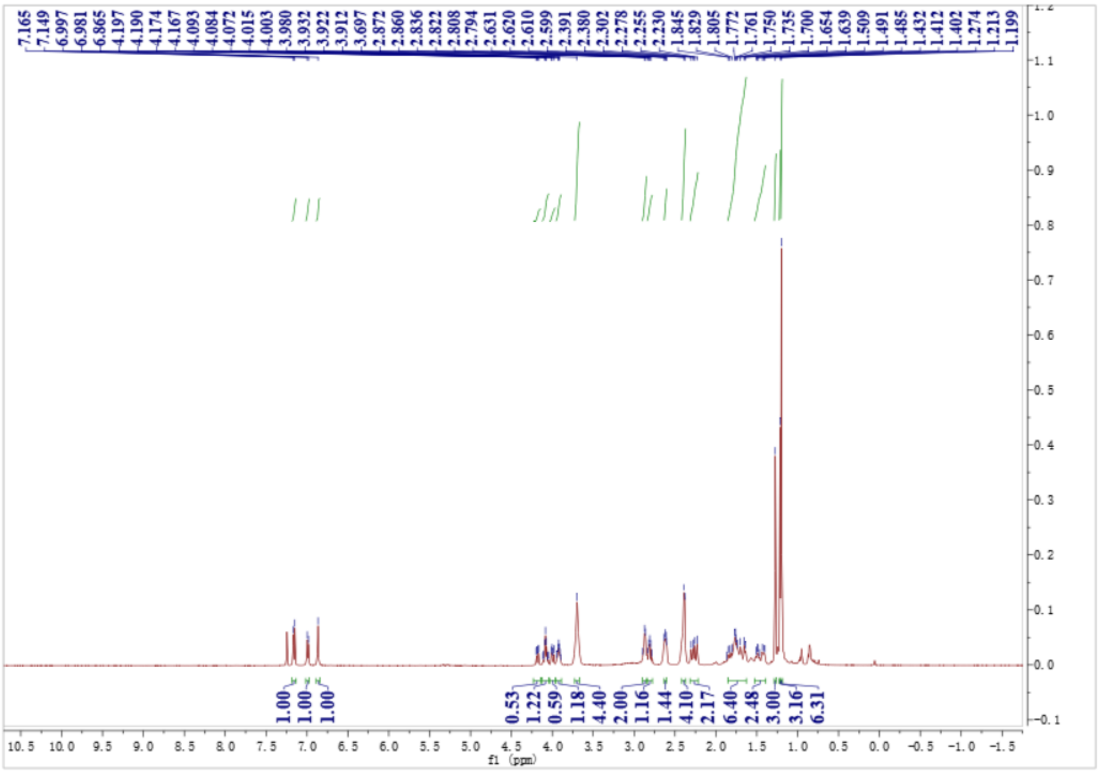

Figure S54. $^{1}$H NMR Spectrum ($CDCl_3$, 500 MHz) of 2p.

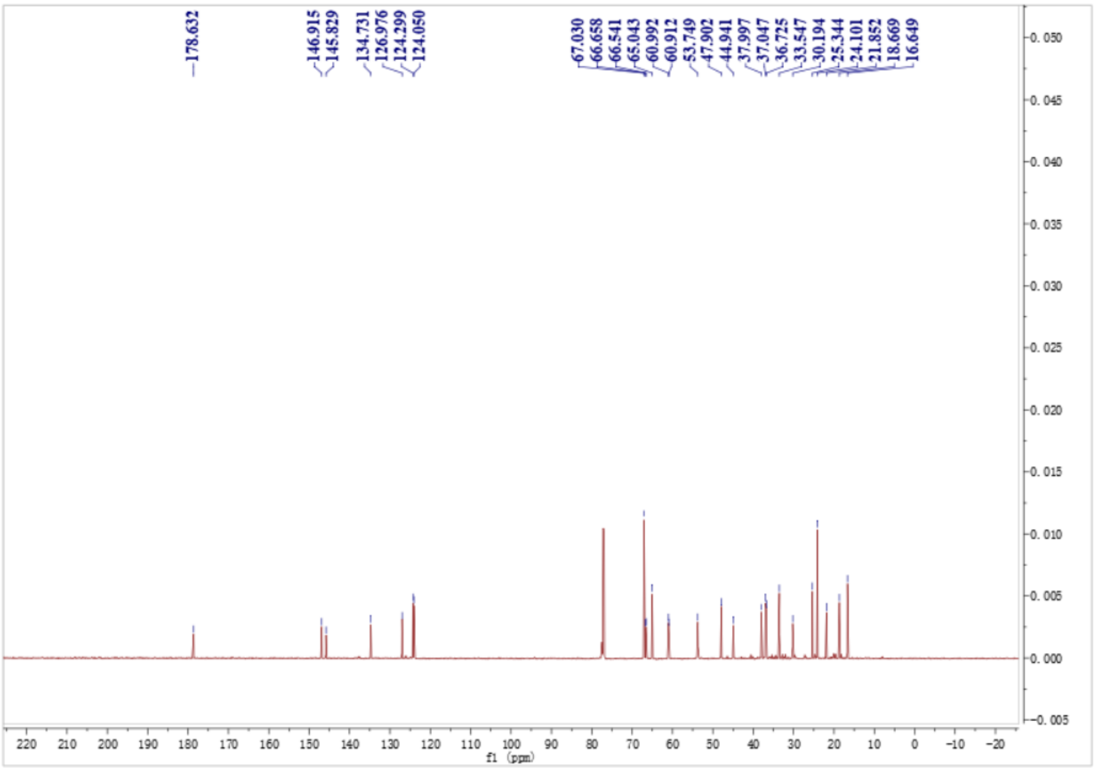

Figure S55. $^{13}$C NMR Spectrum ($CDCl_3$, 126 MHz) of 2p.

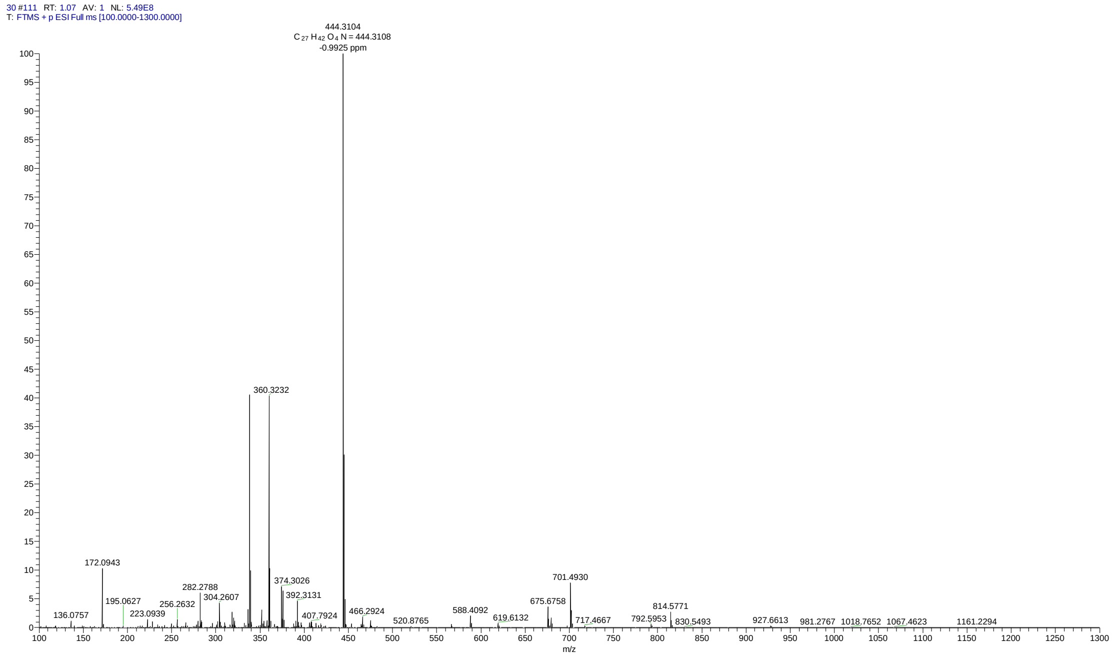


Figure S56. HRMS Spectrum of 2p.

# 3. Figure S57. The physicochemical properties of resulting compounds.

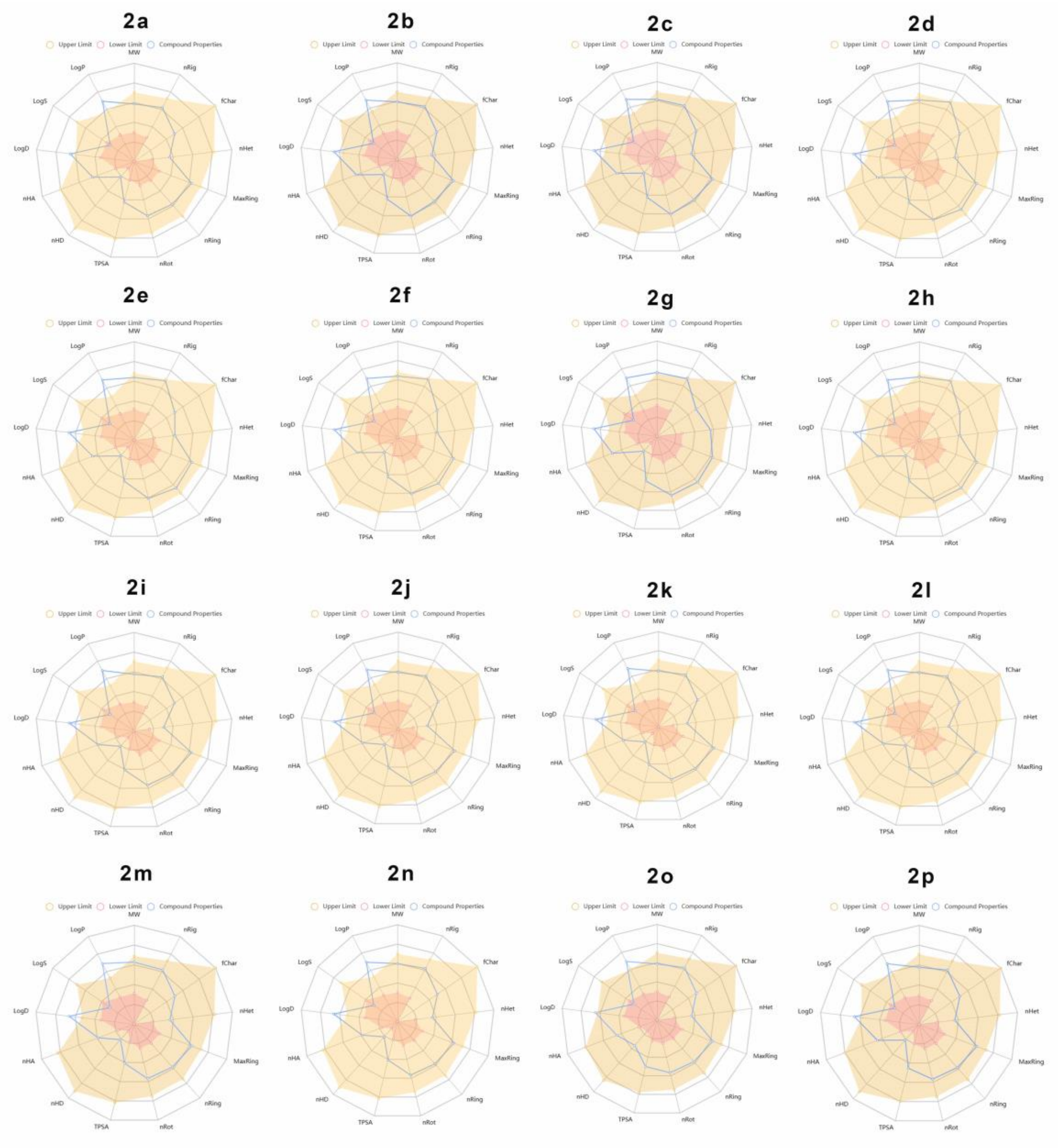
2a
2b
2c
2d
2e
2f
2g
2h
2i
2j
2k
2l
2m
2n
2o
2p
Upper Limit
Lower Limit
Compound Properties
MW
nRig
fChar
nHet
MaxRing
nRing
nRot
TPSA
nHD
nHA
LogD
LogS
LogP
